# Novel 3-(6-methylpyridin-2-yl)coumarin-based chalcones as selective inhibitors of cancer-related carbonic anhydrases IX and XII endowed with anti-proliferative activity

**DOI:** 10.1080/14756366.2022.2056734

**Published:** 2022-04-19

**Authors:** Haytham O. Tawfik, Moataz A. Shaldam, Alessio Nocentini, Rofaida Salem, Hadia Almahli, Sara T. Al-Rashood, Claudiu T. Supuran, Wagdy M. Eldehna

**Affiliations:** a Department of Pharmaceutical Chemistry, Faculty of Pharmacy, Tanta University, Tanta, Egypt; b Department of Pharmaceutical Chemistry, Faculty of Pharmacy, Kafrelsheikh University, Kafrelsheikh, Egypt; c Section of Pharmaceutical and Nutraceutical Sciences, Department of NEUROFARBA, University of Florence, Polo Scientifico, Firenze, Italy; d Department of Chemistry, University of Cambridge, Cambridge, UK; e Department of Pharmaceutical Chemistry, College of Pharmacy, King Saud University, Riyadh, Saudi Arabia

**Keywords:** Anticancer, coumarins, carbonic anhydrase inhibitors, synthesis, metalloenzymes

## Abstract

Carbonic anhydrases (CAs) are one of the promising targets for the development of anticancer agents. CA isoforms are implicated in various physiological processes and are expressed in both normal and cancerous cells. Thus, non-isoform selective inhibitors are associated with several side effects. Consequently, designing selective inhibitors towards cancer-related *h*CA IX/XII rather than the ubiquitous cytosolic isozymes *h*CA I and II is the main research objective in the field. Herein, a new series of 3-(6-methylpyridin-2-yl)coumarin derivatives **3** and **5a–o** was designed and synthesised. The CA inhibition activities for the synthesised coumarins were analysed on isoforms *h*CA I, II, IX, and XII. Interestingly, both cancer-linked isoforms *h*CA IX/XII were inhibited by the prepared coumarins with inhibition constants ranging from sub- to low-micromolar range, whereas *h*CA I and II isoforms haven’t been inhibited up to 100 µM. Furthermore, the target coumarins were assessed for their antitumor activity on NCI-59 human cancer types.

## 1. Introduction

Carbonic anhydrases (CAs) are vital for the processes of CO_2_ hydration and HCO_3_^-^ dehydration^1–3^. The α-CAs are one of the seven known CAs families which are predominantly found in vertebrates, green plants cytoplasm, bacteria, and algae^4^^,^^5^. Among the sixteen human carbonic anhydrases (*h*CAs) isozymes found, the *h*CA IX and XII play a crucial role in the cancer cell persistence by controlling the intracellular pH; thus, their inhibitors are deemed to be an efficient antitumor approach^4^^,^^6^. *h*CA IX expression is associated with a bad prognosis in cancer, whereas *h*CA XII isozyme is expressed in normal tissues and overexpressed in a variety of malignancies^7–10^. Furthermore, non-selective inhibition of *h*CAs leads to some side effects while treating cancer^11^. Consequently, designing selective inhibitors of *h*CA IX/XII rather than the ubiquitous cytosolic isozymes *h*CA I and II is the main target.

Classical CA inhibitors (CAIs) are mostly based on a sulphonamide moiety as a zinc-binding group (ZBG) among which the clinically used CAIs; such as acetazolamide and methazolamide. On the other hand, the non-classical CAIs do not rely on ZBG^11^^,^^12^. Among the non-classical CAIs; coumarins, carboxylic acids, phenols, and polyamines can inhibit the catalytic activity of CA by different mechanisms rather than coordinating to the zinc^13^^,^^14^.

**Figure 1.**
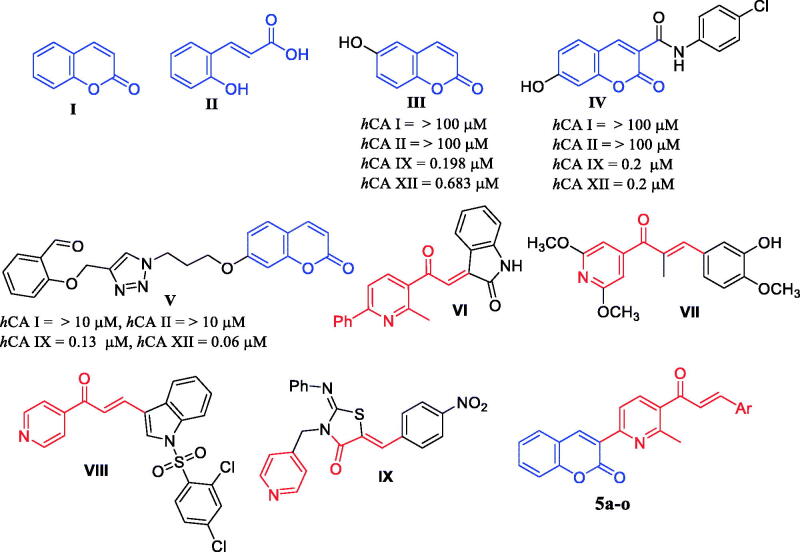
Structure of coumarin **I**, its hydrolysed form **II**, some reported coumarin-based CAIs **III–V**, some reported pyridine derivatives bearing chalcone functionality **VI–IX**, and target compounds **5a–o**.

Coumarin ring, as a privileged scaffold, exerted exceptional anticancer profile acting through various mechanisms of action^15^^,^^16^. Coumarin (**I**, Figure 1) derivatives were introduced by Supuran’ group as a non-classical type of CAIs^17^. Coumarin was shown to undergo hydrolysis to form cis-2-hydroxy-cinnamic acid (**II**, Figure 1), instead of binding the CA active site with its intact coumarin moiety. The substantial selective inhibitory effect towards *h*CA IX and XII is attributable to the binding of the hydrolysis product **II** to the amino acid residues constituting the rim of the active site cavity, which differed significantly between different *h*CA isoforms^12^^,^^17^^,^^18^. These findings grasped the attention for developing a variety of coumarin-based CAIs, such as compounds **III–V** (Figure 1), which exerted efficient and selective inhibition activity towards the cancer-related isozymes IX and XII over the constitutional isozymes CA I and II^18–20^.

On the other hand, pyridine ring is identified as a valuable scaffold for the development of a wide range of approved drugs especially the anticancer ones such imatinib^21^, sorafenib^22^, and acalabrutinib^23^. The pyridine-based small molecules bearing chalcone functionality **VI**-**VIII** (Figure 1) have been described for their *in vitro* anticancer activity against different cancer cell lines^24–27^. In addition, the pyridine derivatives **VIII** and **IX** were able to inhibit the cancer-related CA IX isoform selectively^24–27^.

In this work, the design and synthesis of a series of small molecules based on 3-(6-methylpyridin-2-yl)-coumarin (**MPC**) scaffold as potential selective cancer-associated CA isoform IX/XII inhibitors was achieved (Figure 1). The design of target **MPCs** relies on the incorporation of the coumarin moiety which can exert the CA inhibitory action through obstructing the entry of the active site cavity. Thereafter, the acetyl-bearing pyridine motif was embedded on the coumarin ring as a privileged scaffold in cancer drug discovery to provide **MPC** ketone **3**, which utilised to prepare the target **MPC** chalcones (**5a–o**, Figure 1). The newly prepared series included different lipophilic aromatic rings spanning various ring sizes and different substituents on the aromatic ring, that anticipated to afford lipophilic interactions with the amino acid residing of the rim of the CA active site. The herein synthesised target **MPCs** were evaluated for their CA inhibition activity as well as for their antiproliferative activity towards different 59 cancer cell lines in the US-NCI.

## 2. Results

### 2.1. Chemistry

**Scheme 1.**
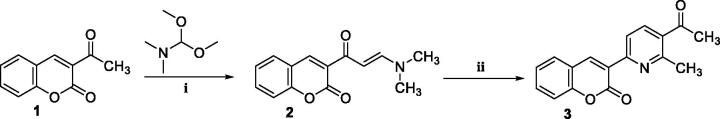
Reagents and conditions: (i) Dry toluene, reflux 7 h.; (ii) Acetylacetone, CH_3_COONH_4_, AcOH, reflux 10 h.

**Scheme 2.**
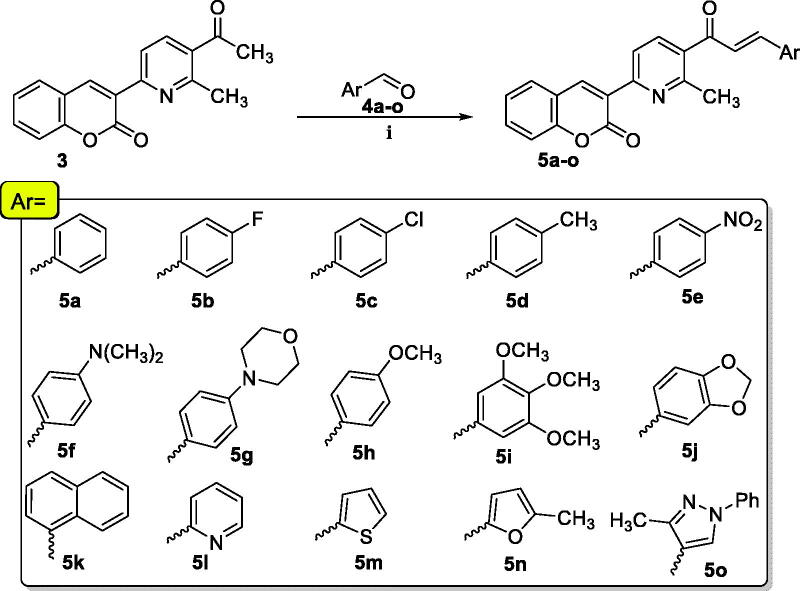
Reagents and conditions: (i) KOH (aq.), dioxane: MeOH stirring at 0 °C 2 h then r.t overnight.

The synthesis strategy for MPC **3** and **5a–o** construction is illustrated in Schemes 1 and 2. 3-Acetylcoumarin **1** was prepared by Knoevenagel condensation through the reaction of salicylaldehyde with ethyl acetoacetate in the presence of piperidine (few drops) as a catalyst according to the reported method^28^. The reaction of 3-acetylcoumarin **1** with dimethyl formamide dimethyl acetal (DMF-DMA) under reflux temperature in dry toluene gave the strategic starting material enaminone **2**.

The condensation of **2** with acetylacetone and ammonium acetate in refluxing acetic acid yielded 3-(5-acetyl-6-methylpyridin-2-yl)-2*H*-chromen-2-one **3**. The chalcones **5a–o** can be readily synthesised *via* the classical base-catalyzed Claisen–Schmidt condensation reaction through the reaction of ketone **3** with various aromatic aldehydes **4a–o** in a mixture of dioxane and methanol as a solvent at 0 °C (Scheme 2).

Sixteen compounds were synthesised in this study, and their structures were confirmed by using IR, ^1^H NMR, and ^13^C NMR (see the Supplementary Material). The elemental analysis results coincide with the molecular formula of target compounds within the accepted range (±0.04%). In the predicted regions of NMR spectra, the methyl (–CH_3_), methylene (–CH_2_–), and methoxy (–OCH_3_) group signals appeared in the aliphatic region for both protons and carbons spectra of the corresponding targets.

### 2.2. Biological evaluation

#### 2.2.1. Carbonic anhydrase isoforms inhibition assay

The newly synthesised **MPCs** (**3** and **5a–o**) were assessed for their CA inhibition activity employing the stopped-flow CO_2_ hydrase assay^29^ for constitutional *h*CA (I/II) isoforms and cancer-linked *h*CA (IX/XII) isoforms. Inhibition values given in Table 1 revealed that the herein-reported **MPC**s have varying degrees of inhibitory action against the examined CA isoforms.

**Table 1.** Inhibition data of **MPC 3** and **5a–o** against *h*CA isoforms I, II, IX, and XII using AAZ as a reference. 
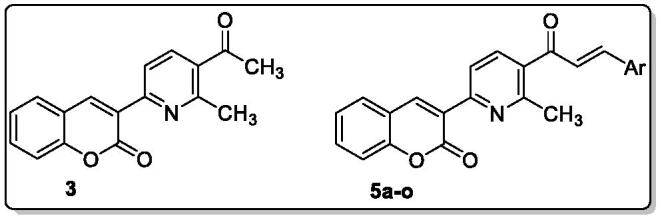

Cpd.	Ar	*KI* (μM)^a,b^			
		CA I	CA II	CA IX	CA XII
**3**	–	>100	>100	0.95	0.68
**5a**	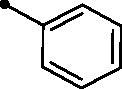	>100	>100	1.5	5.1
**5b**	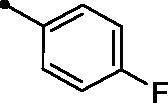	>100	>100	3.4	2.7
**5c**	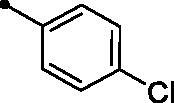	>100	>100	5.8	1.9
**5d**	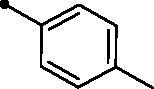	>100	>100	4.3	8.2
**5e**	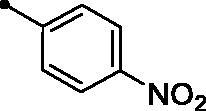	>100	>100	16.4	10.9
**5f**	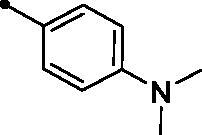	>100	>100	8.5	6.7
**5g**	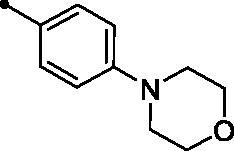	>100	>100	12.0	1.8
**5h**	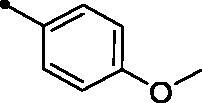	>100	>100	10.7	2.8
**5i**	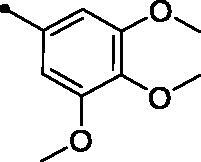	>100	>100	27.4	12.9
**5j**	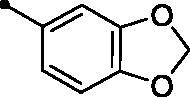	>100	>100	5.3	6.8
**5k**	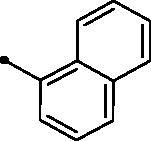	>100	>100	36.9	21.4
**5l**	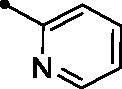	>100	>100	3.8	0.92
**5m**	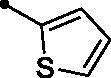	>100	>100	1.1	2.7
**5n**	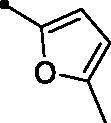	>100	>100	1.5	1.9
**5o**	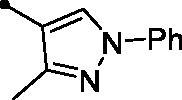	>100	>100	19.4	17.8
**AAZ**		0.25	0.0125	0.025	0.0057

^a^By using a stopped-flow approach, the mean of three different assays was calculated (errors were in the range of 5–10% of the reported values).

^b^Incubation time of 6 h.

The examined **MPCs** displayed one-digit micromolar inhibitory activity against the target cancer-linked isoform IX (*KI*s: 0.95–8.5 µM), except coumarins **5e**, **5g**, **5h**, **5i**, **5k**, and **5o** which displayed two-digit micromolar inhibition activity (*KIs*: 10.7–36.9 µM). It is worth noting that the acetyl derivative **MPC 3** showed the most potent inhibitory action among the tested **MPCs** with sub-micromolar *KI* of 0.95 µM. **MPC 5a** endowed with an unsubstituted phenyl ring displayed low micromolar inhibitory activity (*KI* = 1.5 µM). In addition, the bioisosteric replacement of the phenyl moiety in **5a** with different hetero moieties, such as pyridin-2-yl (**5l**), thiophen-2-yl (**5m**), and 5-methylfuran-2-yl (**5n**) maintained the low micromolar activity towards *h*CA IX isoform (*KIs* = 3.8, 1.1, and 1.5 µM, respectively). On the other hand, replacement of the phenyl ring with fused moieties, such as 1,3-benzodioxol-5-yl and naphtha-1-yl, led to about 3.5- and 23-fold decreased inhibitory activity (compounds **5j** and **5k**; *KIs* = 5.3 and 36.9 µM, respectively).

Moreover, the inhibition potency against *h*CA IX was found to be decreased with varying the size of substituents on the appended phenyl ring in the order of F > CH_3_ > Cl > N(CH_3_)_2_ > OCH_3_ > NO_2_, highlighting that incorporation of small substituents is further valuable for *h*CA IX inhibitory activity over the bulkier ones. In this context, grafting a morpholino or tri-methoxy substituents resulted in the decrease of the activity (compounds **5g** and **5i**; *KIs* = 12.0 and 27.4 µM, respectively) in comparison to the unsubstituted phenyl-bearing analogue **5a** (*KIs* = 1.5 µM).

Further analysis of the inhibition data against *h*CA XII (Table 1) revealed that the target **MPCs 5a–o** were able to affect this isoform with inhibition constants ranging from to sub-micromolar to low micromolar (*KI*s: 0.92–8.2 µM), except **MPCs 5e**, **5i**, **5k**, and **5o** which displayed higher inhibition constant values (*KI*s = 10.9, 12.9, 21.4, and 17.8 µM, respectively). Among the examined **MPC** chalcones **5a–o**, compound **5l** emerged as the unique sub-micromolar *h*CA XII inhibitor (*KI* = 0.92 µM). In addition, **MPCs 5c**, **5g**, and **5n** showed potent inhibitory action with low inhibition constants equal 1.9, 1.8, and 1.9 µM, respectively.

It is worth mentioning that incorporation of an unsubstituted phenyl moiety led to **MPC 5a** with moderate *h*CA XII inhibitory action (*KI* = 5.1 µM), whereas grafting a halogen like *para*-fluoro (**MPC 5b**) and *para*-chloro (**MPC 5c**) improved the inhibitory activity (*KI*s = 2.7 and 1.9 µM, respectively) which highlights that halogens incorporation is beneficial for the *h*CA XII inhibitory effect. Moreover, grafting a *para*-morpholino or *para*-methoxy substituent elicited an enhanced activity (**MPCs 5g** and **5h**; *KI*s = 1.8 and 2.8 µM, respectively) in comparison to the unsubstituted phenyl-bearing counterpart **MPC 5a** (*KI* = 5.1 µM). In addition, the bioisosteric replacement of phenyl motif in **MPC 5a** with different heterocycles, such as the pyridine (**MPC 5l**), thiophene (**MPC 5m**), and furan (**MPC 5n**) moieties boosted the *h*CA XII inhibitory action of the target **MPC** chalcones (*KI*s = 0.92, 2.7, and 1.9 µM, respectively). On the other hand, replacement of the phenyl moiety with the fused naphthyl carbocycle (**MPC 5k**; *KI* = 21.4 µM) or the bulky 3-methyl-1-phenyl-pyrazole heterocycle (**MPC 5o**; *KI* = 17.8 µM) exerted a worsening impact towards the *h*CA XII inhibitory activity.

It is worth stressing that **MPC** ketone **3** established the best inhibitory activity against both *h*CA IX and *h*CA XII isoforms in this study (*KI*s = 0.95 and 0.68 µM, respectively), hinting out the grafting small functionalities within the pyridine ring is more appropriate for the *h*CA inhibitory activity, and should be considered for further optimisation of **MPC** scaffold in the future research.

As expected, both *h*CA I and II isoforms were not inhibited by all newly synthesised **MPC**s which demonstrated inhibition constants more than 100 µM. Accordingly, all the designed **MPC**s showed excellent selectivity towards both cancer-related isoform IX and XII, compared with the cytosolic isoforms (Table 2). Selectivity index (SI) offered obviously presented that **MPC** ketone **3** showed the highest selectivity profile towards *h*CA IX over *h*CA I and II (SI > 105.26) and *h*CA XII over *h*CA I and II (SI > 147.06) followed by **MPC** chalcones **5m**, **5n**, and **5a**, whereas the least selectivity was obtained by the bulky substituted derivatives **5i**, **5k**, and **5o**.

**Table 2.** Selectivity ratios for **MPC 3** and **MPC**s **5a–o** towards cancer-related *h*CA isoforms.

Compounds	Selectivity index (SI)^a,b^ (*K*I off-target CA/*K*I target CA)	
	Towards *h*CA IX	Towards *h*CA XII
**3**	>105.26	>147.06
**5a**	>66.67	>19.61
**5b**	>29.41	>37.04
**5c**	>17.24	>52.63
**5d**	>23.25	>12.19
**5e**	>6.10	>9.17
**5f**	>11.76	>14.92
**5g**	>8.33	>55.56
**5h**	>9.34	>35.71
**5i**	>3.65	>7.75
**5j**	>18.87	>14.71
**5k**	>2.71	>4.67
**5l**	>26.32	>108.70
**5m**	>90.91	>37.04
**5n**	>66.67	>52.63
**5o**	>5.15	>5.62

^a^The *K*I ratios are indicative of isozyme selectivity: a weak selective inhibitor is characterised by a low-value ratio.

^b^Selectivity as determined by the ratio of *K*I for hCA I and II relative to hCA IX and hCA XII.

#### 2.2.2. NCI cancer cell lines screening

Following NCI protocol, sixteen **MPCs** were screened for their potential *in vitro* anticancer effects against human 59 cancer cell panels including prostate, leukaemia melanoma, colon, breast, CNS, renal, NSCLC, and ovarian cancers by National Cancer Institute (USA)^30^.

#### 2.2.3. Preliminary single (10 µM) dose screening

The antiproliferative activities of **MPC 3** and **MPCs 5a–o** were first evaluated in a 10 µM dose assay, with SRB assay used to determine cell survival and proliferation. According to the SRB assay outcomes, most of the newly prepared **MPCs** exerted weak or non-significant anticancer activity towards the majority of examined cells have mean percentages growth inhibition (GI%) range 0–10%, except **MPCs 5g** and **5l** which demonstrated good anti-proliferative activities towards different cancer cell lines (mean% GI = 28 and 50%, respectively). The results of the cell growth inhibitory activities for **MPCs 5g** and **5l** towards the different treated tumour cell lines were presented as GI% and presented in Table 3.

**Table 3.** Cell growth inhibition (GI%) of 59 human tumour cell lines *in vitro* at a dose of 10 µM for **MPCs 5g** and **5l**.

Subpanel cell lines	GI %^a^	
	5g	5l
Leukaemia		
CCRF-CEM	27	93
HL-60(TB)	61	67
K-562	62	92
MOLT-4	33	72
RPMI-8226	–	136
SR	73	112
NSC lung cancer		
A549/ATCC	–	33
EKVX	–	–
HOP-62	–	–
HOP-92	–	–
NCI-H226	–	–
NCI-H23	–	44
NCI-H322M	–	–
NCI-H460	–	58
NCI-H522	38	41
Colon cancer		
COLO 205	–	–
HCC-2998	–	33
HCT-116	–	121
HCT-15	45	133
HT29	–	97
KM 12	31	93
SW-620	–	98
CNS cancer		
SF-268	24	21
SF-295	33	28
SF-539	54	97
SNB-19	20	28
SNB-75	41	43
U251	–	92
Melanoma		
LOX IMVI	68	184
MALME-3M	42	–
M14	54	–
SK-MEL-28	27	–
SK-MEL-5	28	–
MDA-MB-435	121	29
SK-MEL-2	–	–
UACC-62	47	28
UACC-257	–	–
Ovarian cancer		
OVCAR-4	27	–
OVCAR-5	–	39
IGROV1	–	93
OVCAR-3	–	53
SK-OV-3	–	–
OVCAR-8	–	30
NCI/ADR-RES	32	22
Renal cancer		
786-0	24	93
A498	24	–
ACHN	29	41
CAKI-1	34	39
RXF 393	–	67
SN 12 C	–	52
UO-31	–	41
Prostate cancer		
PC-3	–	31
DU-145	–	52
Breast cancer		
MCF7	48	91
MDA-MB-231	45	87
HS 578 T	40	–
BT-549	23	–
T-47D	–	47
MDA-MB-468	54	39
Sensitive cells no.	31	42

^a^Only GI % more than 20% are displayed.

Assessing the obtained GI % values (Table 3) revealed that **MPC 5l** is the most effective anti-proliferative agent among the compounds described here. The NCI screening results revealed anti-proliferative efficacy against 42 human cancer cell lines, indicating that this compound has broad-spectrum activity.

**MPC 5l** showed remarkable growth inhibition properties against Leukaemia (K-562/CCRF-CEM), Colon (HT29, KM 12, and SW-620), CNS (U251 and SF-539), Ovarian (IGROVI), Breast (MDA-MB-231 and MCF7) Renal (786-0) cancer cell lines, with inhibition % 93, 92, 93, 91, and 87%, respectively (Table 3). **MPC 5l** also showed strong efficacy towards leukaemia [MOLT-4/HL-60(TB)] and Renal (RXF 393) tumour cell lines, with inhibition percentages of 67, 72, and 67%, respectively. It is noteworthy that **MPC 5l** was shown to be lethal towards Leukaemia (RPMI-8226 and SR), Colon (HCT-15/HCT-116), and LOX IMVI Melanoma cells (GI % = 136, 112, 121, 133, and 184, respectively).

NCI screening results for **MPC 5g** showed anti-proliferative activity against 31 human cancer cell lines indicating a broad-spectrum activity. Compound **5g** exerted its lethal action towards Melanoma MDAMB-435 cells with GI % = 121. Moreover, compound **5g** exerted good activity towards Leukaemia [K-562, HL-60(TB), and SR] and (LOX IMVI) Melanoma cells (inhibition % 61, 62, 73, and 68, respectively). Additionally, compound **5g** exerted moderate activity towards Colon cancer (HCT-15), CNS cancer (SF-539 and SNB-75), Melanoma (MALME-3M, M14 and UACC-62) and Breast (MDA-MB-468, MCF7, HS 578T, and MDA-MB-231) cancer cells with inhibition % 45, 54, 41, 42, 54, 47, 48, 45, 40, and 54, respectively (Table 3).

**Figure 2.**
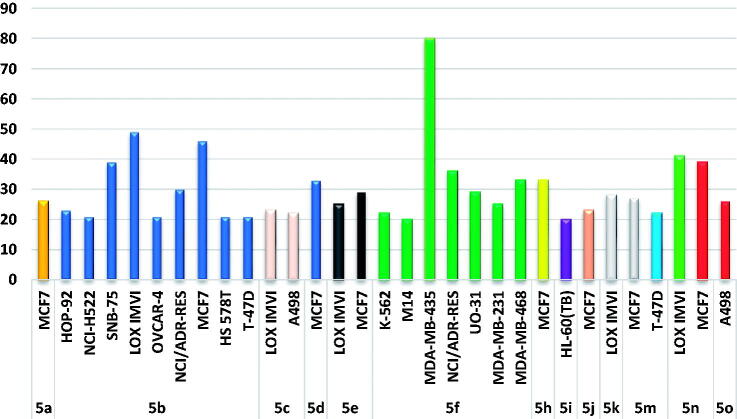
The best anti-proliferative activities exerted by target MPC chalcones **5a–f**, **5h–k**, and **5m–o**.

On the other hand, the obtained results for the remaining MPC chalcones **5a–f**, **5h–k**, and **5m–o** ascribed to these derivatives selective actions towards certain cancer cell lines, as displayed in Figure 2. In particular, compound **5b** showed selective anticancer activity towards CNS cancer (SNB-75), Breast (MCF7), Melanoma (LOX IMVI) cells with inhibition % 39, 49, and 46, respectively. Also, compound **5f** displayed good selectivity towards Melanoma (MDA-MB-435) cells (inhibition % = 80), whereas, compound **5n** has selectivity towards Breast (MCF7) and Melanoma (LOX IMVI) cells (inhibition % 40 and 39, respectively).

#### 2.2.4. *In vitro* full NCI panel five dose assay

The preliminary single-dose assay results show that **MPC 5l** (NSC: 831974/1) is the most effective anticancer drug in this investigation, with promising inhibitory activity against a variety of cancer cell lines from various subpanels (Table 3). **MPC 5l** was then chosen for additional biological evaluation in a five-dose (0.01–100 µM) experiment. **MPC 5l**'s GI_50_, TGI, and LC_50_ response parameters were obtained for each of the cancer cell lines studied. TGI represents cytostatic impact, whereas GI_50_ values reflect the extent of growth inhibitory effect. Furthermore, the LC_50_ parameter is regarded as the cytotoxicity parameter for the hybrid under investigation.

As shown in Table 4, **MPC 5l** had a potent anti-proliferative effect against nine human cancer cell lines tested: leukaemia (K-562, RPMI-8226, and SR), NSCLC (HOP-92), breast cancer (MCF7), colon (SW-620 and HCT-116) cancer, melanoma (LOX IMVI), and CNS (U251) with GI50 values ranging from 3.20 to 8.49 µM. **MPC 5l**, on the other hand, had GI_50_ > 100 µM against remaining cancer cells. Furthermore, **MPC 5l** demonstrated no cytostatic effect on all cancer cell lines (TGI > 100 µM). **MPC 5l** was discovered to be a non-lethal molecule with LC_50_ > 100 µM against all cancer cells.

**Table 4.** Results of the five-dose anticancer assay for **MPC 5l** against all fifty-nine cancer cell lines.

Panel	Cell line	MPC 5l		
		GI_50_ (μM)	TGI (μM)	IC_50_ (μM)
Leukaemia	K-562	6.47	>100	>100
	RPMI-8226	3.41	>100	>100
	SR	5.30	>100	>100
NSC lung cancer	HOP-92	7.25	>100	>100
Colon cancer	HCT-116	6.34	>100	>100
	SW-620	6.77	>100	>100
CNS cancer	U251	8.49	>100	>100
Melanoma	LOX IMVI	3.20	>100	>100
Breast cancer	MCF7	3.72	>100	>100

## 3. Conclusions

In brief, the present study demonstrates the design and synthesis of novel 6-(methylpyridin-2-yl)-coumarins **MPC 3** and **MPC** (**5a–o**) as selective *h*CAIs. The synthesised target compounds selectively inhibited the cancer-related *h*CA isoforms with *KI* ranges: 0.95–36.9 µM (*h*CA IX) and 0.68–21.4 µM (*h*CA XII). All the designed MPCs showed excellent selectivity for *h*CA IX/*h*CA XII, over the cytosolic ones *h*CA I and *h*CA II with **MPC 3** being the highest (SI towards *h*CA IX over *h*CA I and II > 105.26 and SI towards *h*CA XII over *h*CA I and II > 147.06). The SAR results emphasised that e grafting small functionalities within the pyridine ring is more appropriate for the *h*CA inhibitory activity. *In vitro* antitumor effects *vs.* various human cancer cells were also investigated, and **5l** was found to have outstanding growth suppression characteristics against CNS, Colon, Ovarian, Breast, Leukaemia, and Renal cancer. **MPC 5l** was then chosen for further biological testing using a five-dose assay. The results showed that a single-digit micromolar concentration of the compound **5l** had a potent anti-proliferative effect against nine human cancer cell lines, including leukaemia, NSCL cancer, colon cancer, CNS cancer, melanoma, and breast cancer, with GI_50_ values ranging from 3.20 to 8.49 µM.

## 4. Materials and methods

### 4.1. Chemistry

Melting points were measured in open-glass capillaries using a Stuart SMP30 apparatus at Tanta University's Faculty of Pharmacy's Central Research Laboratory in Tanta, Egypt. All organic chemicals and solvents were acquired from Sigma–Aldrich, Alfa Aesar, and Merck, respectively, and utilised without further purification. Analytical thin-layer chromatography (TLC): pre-coated aluminium sheets, 0.2 mm silica gel (Supelco Co., Silica 60 F_254_) used regularly to monitor reaction progress and ensure product purity utilising a developing system: The eluent was chloroform: methanol (2:1), which was visualised using a UV lamp set to 254 nm. The FT-IR spectra were detected on a ThermoFisher Scientific Nicolet-iS10 Spectrometer (MA, USA). ^1^H and ^13^C NMR spectra were carried out utilising the Bruker instrument at 400–500 MHz for ^1^H NMR and at 100–125 MHz for ^13^C NMR spectrophotometer, TMS is being used as an internal standard and chemical shifts were recorded in ppm on the δ scale using CDCl_3_-*d* as a solvent. The values of the coupling constant (*J*) were calculated in Hertz (Hz). The following are the split patterns: s, singlet; d, doublet; t, triplet; q, quartette; m, multiplet. Microanalysis was performed for C, H, and N elements on PerkinElmer 2400 (The regional centre for mycology and biotechnology, Al-Azhar University, Nasr City, Cairo, Egypt).

#### 4.1.1. Synthesis of 3-[(2E)-3-(dimethylamino)prop-2-enoyl]-2H-chromen-2-one (2)

3-Acetyl-2-*H*-chromen-2-one **1** (1.88 g, 0.01 mol) and dimethylformamide-dimethylacetal (DMF-DMA) (1.19 g, 0.01 mol) were heated in dry toluene (10 ml) for 7 h at 110 °C. The cooled reaction mixture was filtered, washed with diethyl ether, dried, and crystallised from ethanol to yield compound **2** as a yellow powder (1.78 g, 73%). Mp: 159–161 °C^31^.

#### 4.1.2. Synthesis of 3-(5-acetyl-6-methylpyridin-2-yl)-2H-chromen-2-one (3)

In *gl.* AcOH (20 ml), an equimolar amount of enaminone **2** (1.70 g, 7 mmol), and acetylacetone (0.7 g, 7 mmol) was heated under reflux for 10 h in the presence of ammonium acetate (0.77 g, 10 mmol). The resultant product was collected, washed twice with water (2 × 10 ml), and recrystallized from acetonitrile to produce MPC ketone **3**^32^.

A yellow powder, yield: 70%. Mp: 208–210 °C. ^1^H NMR (500 MHz, CDCl_3_-*d*) δ: 2.63 (s, 3H, CH_3_), 2.84 (s, 3H, CO CH_3_), 7.34 (t, 1H, Arm. H, *J* = 8.0 Hz), 7.40 (d, 1H, Arm. H, *J* = 8.0 Hz), 7.59 (t, 1H, Arm. H, *J* = 8.0 Hz), 7.70 (d, 1H, Arm. H, *J* = 8.0 Hz), 8.08 (d, 1H, Arm. H, *J* = 8.0 Hz), 8.42 (d, 1H, Arm. H, *J* = 8.0 Hz), 8.93 (s, 1H, 4-H of coumarin ring).

#### 4.1.3. General procedure for preparation of MPCs 5a–o

At 0 °C, a stirred solution of ketone **3** (0.5 mmol) and the suitable aldehyde (0.5 mmol) in a mixture of dioxane: methanol (4:2) (25 ml) was added to aqueous potassium hydroxide solution (0.15 g, in 1.5 ml dist. water).

The resulting mixture was agitated for 2 h at 0 °C before being warmed to room temperature overnight. The solvent was extracted under vacuum after the reaction was neutralised with *gl.* AcOH. **MPCs 5a–o** were produced by filtering the precipitate, washing it with diethyl ether, drying it, and crystallising it from ethanol.

##### 4.1.3.1. 3-(6-Methyl-5-[(2E)-3-phenylprop-2-enoyl]pyridin-2-yl)-2H-chromen-2-one (5a)

A yellow powder, yield: 85%. Mp: 207–209 °C. IR (*ν*_max_/cm^−1^): 3058 (CH-arom.), 2924, 2854 (CH-aliph.), 1724, 1665 (2 C = O). ^1^H NMR (500 MHz, CDCl_3_-*d*) δ: 2.74 (s, 3H, CH_3_), 7.16 (d, 1H, COC**H**=CH, *J* = 16.0 Hz), 7.35 (t, 1H, Arm. H, *J* = 8.0 Hz), 7.40–7.43 (m, 4H, Arm. H), 7.52 (d, 1H, COCH=C**H**, *J* = 16.0 Hz), 7.58–7.61 (m, 3H, Arm. H), 7.71 (d, 1H, Arm. H, *J* = 8.0 Hz), 7.90 (d, 1H, Arm. H, *J* = 8.0 Hz), 8.40 (d, 1H, Arm. H, *J* = 8.0 Hz), 8.91 (s, 1H, 4-H of coumarin ring). ^13^C NMR (125 MHz, CDCl_3_-*d*) δ: 23.77, 116.41, 119.42, 120.66, 124.50, 124.70, 125.98, 128.58 (2 C), 129.07 (2 C), 129.25, 131.08, 132.48, 133.55, 134.17, 136.57, 143.35, 146.88, 151.75, 153.98, 156.75, 160.40, 194.77. Anal. calcd. for C_24_H_17_NO_3_: C, 78.46; H, 4.66; N, 3.81. Found: C, 78.22; H, 4.61; N, 3.80.

##### 4.1.3.2. 3-(5-[(2E)-3-(4-Fluorophenyl)prop-2-enoyl]-6-methylpyridin-2-yl)-2H-chromen-2-one (5b)

A pale-yellow powder, yield: 66%. Mp: 205–207 °C. ^1^H NMR (500 MHz, CDCl_3_-*d*) δ: 2.74 (s, 3H, CH_3_), 7.09 (d, 1H, COC**H**=CH, *J* = 16.0 Hz), 7.13 (d, 2H, Arm. H, *J* = 8.0 Hz), 7.36 (t, 1H, Arm. H, *J* = 8.0 Hz), 7.40 (d, 1H, Arm. H, *J* = 8.0 Hz), 7.49 (d, 1H, COCH = C**H**, *J* = 16.0 Hz), 7.57–7.61 (m, 3H, Arm. H), 7.71 (d, 1H, Arm. H, *J* = 8.0 Hz), 7.89 (d, 1H, Arm. H, *J* = 8.0 Hz), 8.40 (d, 1H, Arm. H, *J* = 8.0 Hz), 8.91 (s, 1H, 4-H of coumarin ring). ^13^C NMR (125 MHz, CDCl_3_-*d*) δ: 23.78, 116.23, 116.40, 116.43, 119.43, 120.67, 124.57, 124.72, 125.69, 129.10, 130.51, 130.57, 132.51, 133.45, 136.54, 143.38, 145.42, 151.81, 154.01, 156.80, 160.27, 163.33, 165.34, 194.44. Anal. calcd for C_24_H_16_FNO_3_: C, 74.80; H, 4.18; N, 3.63. Found: C, 74.97; H, 4.14; N, 3.59.

##### 4.1.3.3. 3-(5-[(2E)-3-(4-Chlorophenyl)prop-2-enoyl]-6-methylpyridin-2-yl)-2H-chromen-2-one (5c)

A yellow powder, yield: 75%. Mp: 223–225 °C. IR (*ν*_max_/cm^−1^): 3064 (CH-arom.), 2966, 2925 (CH-aliph.), 1712, 1660 (2 C = O). ^1^H NMR (500 MHz, CDCl_3_-*d*) δ: 2.74 (s, 3H, CH_3_), 7.13 (d, 1H, COC**H**=CH, *J* = 16.0 Hz), 7.35 (t, 1H, Arm. H, *J* = 8.0 Hz), 7.39–7.41 (m, 3H, Arm. H), 7.46 (d, 1H, COCH = C**H**, *J* = 16.0 Hz), 7.51 (d, 2H, Arm. H, *J* = 8.0 Hz), 7.60 (t, 1H, Arm. H, *J* = 8.0 Hz), 7.71 (d, 1H, Arm. H, *J* = 8.0 Hz), 7.90 (d, 1H, Arm. H, *J* = 8.0 Hz), 8.40 (d, 1H, Arm. H, *J* = 8.0 Hz), 8.92 (s, 1H, 4-H of coumarin ring). ^13^C NMR (125 MHz, CDCl_3_-*d*) δ: 23.82, 116.64, 119.42, 120.68, 124.54, 124.72, 126.29, 129.11, 129.39 (2 C), 129.69 (2 C), 132.53, 132.71, 133.33, 136.59, 137.03, 143.42, 145.16, 151.89, 154.02, 156.88, 160.27, 194.30. Anal. calcd for C_24_H_16_ClNO_3_: C, 71.73; H, 4.01; N, 3.49. Found: C, 71.95; H, 3.97; N, 3.52.

##### 4.1.3.4. 3-(6-Methyl-5-[(2E)-3-(4-methylphenyl)prop-2-enoyl]pyridin-2-yl)-2H-chromen-2-one (5d)

A yellow powder, yield: 71%. Mp: 206–208 °C. IR (*ν*_max_/cm^−1^): 3054 (CH-arom.), 2967, 2922 (CH-aliph.), 1727, 1661 (2 C = O). ^1^H NMR (400 MHz, CDCl_3_-*d*) δ: 2.42 (s, 3H, CH_3_), 2.78 (s, 3H, CH_3_), 7.13 (d, 1H, COC**H**=CH, *J* = 16.0 Hz), 7.25 (d, 1H, Arm. H, *J* = 8.0 Hz), 7.27 (d, 1H, Arm. H, *J* = 8.0 Hz), 7.37 (t, 1H, Arm. H, *J* = 8.0 Hz), 7.42 (d, 1H, Arm. H, *J* = 8.0 Hz), 7.51 (d, 1H, COCH = C**H**, *J* = 16.0 Hz), 7.52 (d, 2H, Arm. H, *J* = 8.0 Hz), 7.62 (t, 1H, Arm. H, *J* = 8.0 Hz), 7.73 (d, 1H, Arm. H, *J* = 8.0 Hz), 7.92 (d, 1H, Arm. H, *J* = 8.0 Hz), 8.42 (d, 1H, Arm. H, *J* = 8.0 Hz), 8.95 (s, 1H, 4-H of coumarin ring). ^13^C NMR (100 MHz, CDCl_3_-*d*) δ: 21.63, 116.50 (2 C), 119.15, 121.89, 124.72, 124.98 (2 C), 128.79 (2 C), 129.62, 129.93 (2 C), 131.28, 133.12, 134.59, 138.04, 142.17, 145.08, 147.84, 150.97, 154.22, 156.42, 159.94, 193.69. Anal. calcd for C_25_H_19_NO_3_: C, 78.72; H, 5.02; N, 3.67. Found: C, 79.02; H, 4.97; N, 3.65.

##### 4.1.3.5. 3-(6-Methyl-5-[(2E)-3-(4-nitrophenyl)prop-2-enoyl]pyridin-2-yl)-2H-chromen-2-one (5e)

A red powder, yield: 57%. Mp: 202–204 °C. ^1^H NMR (500 MHz, CDCl_3_-*d*) δ: 2.74 (s, 3H, CH_3_), 7.14 (d, 1H, COC**H**=CH, *J* = 16.0 Hz), 7.36 (t, 1H, Arm. H, *J* = 8.0 Hz), 7.41 (d, 3H, Arm. H, *J* = 8.0 Hz), 7.48 (d, 1H, COCH = C**H**, *J* = 16.0 Hz), 7.53 (d, 2H, Arm. H, *J* = 8.0 Hz), 7.61 (t, 1H, Arm. H, *J* = 8.0 Hz), 7.71 (d, 1H, Arm. H, *J* = 8.0 Hz), 7.90 (d, 1H, Arm. H, *J* = 8.0 Hz), 8.41 (d, 1H, Arm. H, *J* = 8.0 Hz), 8.91 (s, 1H, 4-H coumarin ring). ^13^C NMR (125 MHz, CDCl_3_-*d*) δ: 23.81, 116.43, 119.41, 120.67, 124.53, 124.72, 126.27, 129.10, 129.38 (2 C), 129.69 (2 C), 132.53, 132.69, 133.31, 136.59, 137.02, 143.43, 145.18, 151.88, 154.00, 156.87, 160.27, 194.31. Anal. calcd for C_24_H_16_N_2_O_5_: C, 69.90; H, 3.91; N, 6.79. Found: C, 70.11; H, 3.90; N, 6.83.

##### 4.1.3.6. 3-(5-[(2E)-3-[4-(Dimethylamino)phenyl]prop-2-enoyl]-6-methylpyridin-2-yl)-2H-chromen-2-one (5f)

An orange powder, yield: 73%. Mp: 196–198 °C. ^1^H NMR (500 MHz, CDCl_3_-*d*) δ: 2.71 (s, 3H, CH_3_), 3.05 (s, 6H, N(CH_3_)_2_), 6.68 (d, 2H, Arm. H, *J* = 8.0 Hz), 6.92 (d, 1H, COC**H**=CH, *J* = 16.0 Hz), 7.34 (t, 1H, Arm. H, *J* = 8.0 Hz), 7.40 (d, 1H, Arm. H, *J* = 8.0 Hz), 7.41 (d, 1H, COCH = C**H**, *J* = 16.0 Hz), 7.27 (d, 2H, Arm. H, *J* = 8.0 Hz), 7.58 (t, 1H, Arm. H, *J* = 8.0 Hz), 7.70 (d, 1H, Arm. H, *J* = 8.0 Hz), 7.83 (d, 1H, Arm. H, *J* = 8.0 Hz), 8.35 (d, 1H, Arm. H, *J* = 8.0 Hz), 8.87 (s, 1H, 4-H coumarin ring). ^13^C NMR (125 MHz, CDCl_3_-*d*) δ: 23.51, 40.07 (2 C), 111.79 (2 C), 116.37, 119.50, 120.62, 121.15, 121.75, 124.64, 124.87, 129.03, 130.65 (2 C), 132.29, 134.67, 136.22, 143.03, 148.28, 151.16, 152.34, 153.94, 156.30, 160.34, 195.20. Anal. calcd for C_26_H_22_N_2_O_3_: C, 76.08; H, 5.40; N, 6.82. Found: C, 75.83; H, 5.46; N, 6.84.

##### 4.1.3.7. 3-(6-Methyl-5-[(2E)-3-[4-(morpholin-4-yl)phenyl]prop-2-enoyl]pyridin-2-yl)-2H-chromen-2-one (5g)

A yellow powder, yield: 60%. Mp: 208–210 °C. IR (*ν*_max_/cm^−1^): 3065 (CH-arom.), 2958, 2918 (CH-aliph.), 1727, 1656 (2 C = O). ^1^H NMR (500 MHz, CDCl_3_-*d*) δ: 2.72 (s, 3H, CH_3_), 3.28 (t, 4H, morpholinyl ring, *J* = 5.0 Hz), 3.86 (t, 4H, morpholinyl ring, *J* = 5.0 Hz), 6.88 (d, 2H, Arom. H, *J* = 8.0 Hz), 6.99 (d, 1H, COC**H**=CH, *J* = 16.0 Hz), 7.34 (t, 1H, Arom. H, *J* = 8.0 Hz), 7.40 (d, 1H, Arom. H, *J* = 8.0 Hz), 7.42 (d, 1H, COCH = C**H**, *J* = 16.0 Hz), 7.50 (d, 2H, Arom. H, *J* = 8.0 Hz), 7.59 (t, 1H, Arom. H, *J* = 8.0 Hz), 7.70 (d, 1H, Arom. H, *J* = 8.0 Hz), 7.85 (d, 1H, Arom. H, *J* = 8.0 Hz), 8.37 (d, 1H, Arom. H, *J* = 4.0 Hz), 8.88 (s, 1H, 4-H of coumarin ring). ^13^C NMR (125 MHz, CDCl_3_-*d*) δ: 23.59, 47.74 (2 C), 66.56 (2 C), 114.48 (2 C), 116.39, 119.47, 120.63, 122.80, 124.67, 124.75, 124.87, 129.05, 130.35 (2 C), 132.38, 134.23, 136.32, 143.15, 147.29, 151.38, 153.04, 153.96, 156.45, 160.31, 195.05. Anal. calcd for C28H24N2O4: C, 74.32; H, 5.35; N, 6.19. Found: C, 74.20; H, 5.37; N, 6.24.

##### 4.1.3.8. 3-(5-[(2E)-3-(4-Methoxyphenyl)prop-2-enoyl]-6-methylpyridin-2-yl)-2H-chromen-2-one (5h)

A yellow powder, yield: 66%. Mp: 174–175 °C. IR (*ν*_max_/cm^−1^): 3058 (CH-arom.), 2965, 2931 (CH-aliph.), 1725, 1660 (2 C = O). ^1^H NMR (400 MHz, CDCl_3_-*d*) δ: 2.77 (s, 3H, CH_3_), 3.88 (s, 3H, O CH_3_), 6.96 (d, 2H, Arom. H, *J* = 8.0 Hz), 7.05 (d, 1H, COC**H**=CH, *J* = 16.0 Hz), 7.37 (t, 1H, Arom. H, *J* = 8.0 Hz), 7.41 (d, 1H, Arom. H, *J* = 8.0 Hz), 7.48 (d, 1H, COCH = C**H**, *J* = 16.0 Hz), 7.57 (d, 1H, Arom. H, *J* = 8.0 Hz), 7.62 (t, 1H, Arom. H, *J* = 8.0 Hz), 7.74 (d, 1H, Arom. H, *J* = 8.0 Hz), 7.90 (d, 2H, Arom. H, *J* = 8.0 Hz), 8.42 (d, 1H, Arom. H, *J* = 8.0 Hz), 8.94 (s, 1H, 4-H of coumarin ring). ^13^C NMR (125 MHz, CDCl_3_-*d*) δ: 23.65, 55.43, 114.53 (2 C), 116.39, 119.44, 120.63, 123.87, 124.68 (2 C), 126.85, 129.07, 130.43 (2 C), 132.41, 133.94, 136.40, 143.24, 146.90, 151.53, 153.90, 156.55, 160.20, 162.08, 194.95. Anal. calcd for C_25_H_19_NO_4_: C, 75.55; H, 4.82; N, 3.52. Found: C, 75.38; H, 4.83; N, 3.54.

##### 4.1.3.9. 3-(6-Methyl-5-[(2E)-3-(3,4,5-trimethoxyphenyl)prop-2-enoyl]pyridin-2-yl)-2H-chromen-2-one (5i)

A yellow powder, yield: 86%. Mp: 186–188 °C. IR (*ν*_max_/cm^−1^): 3062 (CH-arom.), 2999, 2934, 2839 (CH-aliph.), 1727, 1666 (2 C = O). ^1^H NMR (500 MHz, CDCl_3_-*d*) δ: 2.72 (s, 3H, CH_3_), 3.90 (s, 9H, 3 of O CH_3_), 6.80 (s, 2H, Arom. H), 7.02 (d, 1H, COC**H**=CH, *J* = 16.0 Hz), 7.35 (t, 1H, Arom. H, *J* = 8.0 Hz), 7.40 (d, 1H, COCH = C**H**, *J* = 16.0 Hz), 7.41 (d, 1H, Arom. H, *J* = 8.0 Hz), 7.60 (t, 1H, Arom. H, *J* = 8.0 Hz), 7.71 (d, 1H, Arom. H, *J* = 8.0 Hz), 7.87 (d, 1H, Arom. H, *J* = 8.0 Hz), 8.38 (d, 1H, Arom. H, *J* = 8.0 Hz), 8.89 (s, 1H, 4-H of coumarin ring). ^13^C NMR (125 MHz, CDCl_3_-*d*) δ: 23.60, 56.17 (2 C), 61.01, 105.65 (2 C), 116.42, 119.41, 120.63, 124.65, 124.73, 125.63, 129.07, 129.55, 132.50, 133.58, 136.43, 140.75, 143.34, 147.27, 151.69, 153.49 (2 C), 153.96, 156.59, 160.36, 195.03. Anal. calcd for C_27_H_23_NO_6_: C, 70.89; H, 5.07; N, 3.06. Found: C, 71.07; H, 5.02; N, 3.08.

##### 4.1.3.10. 3-(5-[(2E)-3-(2H-1,3-Benzodioxol-5-yl)prop-2-enoyl]-6-methylpyridin-2-yl)-2H-chromen-2-one (5j)

A green powder, yield: 84%. Mp: 178–180 °C. IR (*ν*_max_/cm^−1^): 3064 (CH-arm.), 2970, 2903 (CH-aliph.), 1723, 1657 (2 C = O). ^1^H NMR (500 MHz, CDCl_3_-*d*) δ: 2.72 (s, 3H, CH_3_), 6.03 (s, 2H, OCH2O), 6.84 (d, 1H, Arm. H, *J* = 8.0 Hz), 6.98 (d, 1H, COC**H**=CH, *J* = 16.0 Hz), 7.05 (d, 1H, Arm. H, *J* = 8.0 Hz), 7.10 (s, 1H, Arm. H), 7.33 (t, 1H, Arm. H, *J* = 8.0 Hz), 7.40 (d, 1H, Arm. H, *J* = 8.0 Hz), 7.42 (d, 1H, COCH = C**H**, *J* = 16.0 Hz), 7.60 (t, 1H, Arm. H, *J* = 8.0 Hz), 7.70 (d, 1H, Arm. H, *J* = 8.0 Hz), 7.86 (d, 1H, Arm. H, *J* = 8.0 Hz), 8.37 (d, 1H, Arm. H, *J* = 4.0 Hz), 8.89 (s, 1H, 4-H coumarin ring). ^13^C NMR (125 MHz, CDCl_3_-*d*) δ: 23.70, 101.74, 106.61, 108.73, 116.39, 119.42, 120.64, 124.10, 124.62, 124.68, 125.63, 128.62, 129.07, 132.43, 133.81, 136.43, 143.27, 146.73, 148.51, 150.36, 151.60, 153.96, 156.62, 160.27, 194.66. Anal. calcd for C_25_H_17_NO_5_: C, 72.99; H, 4.16; N, 3.40. Found: C, 73.23; H, 4.14; N, 3.42.

##### 4.1.3.11. 3-(6-Methyl-5-[(2E)-3-(naphthalen-1-yl)prop-2-enoyl]pyridin-2-yl)-2H-chromen-2-one (5k)

A yellow powder, yield: 72%. Mp: 204–206 °C. IR (*ν*_max_/cm^−1^): 3040 (CH-arom.), 2962, 2923 (CH-aliph.), 1721, 1660 (2 C = O). ^1^H NMR (500 MHz, CDCl_3_-*d*) δ: 2.82 (s, 3H, CH_3_), 7.29 (d, 1H, COC**H**=CH, *J* = 16.0 Hz), 7.35 (t, 1H, Arm. H, *J* = 8.0 Hz), 7.41 (d, 1H, Arm. H, *J* = 8.0 Hz), 7.53–7.60 (m, 4H, Arm. H), 7.72 (d, 1H, Arm. H, *J* = 8.0 Hz), 7.90 (d, 1H, Arm. H, *J* = 8.0 Hz), 7.95 (d, 2H, Arm. H, *J* = 8.0 Hz), 8.01 (d, 1H, Arm. H, *J* = 8.0 Hz), 8.12 (d, 1H, Arm. H, *J* = 8.0 Hz), 8.44 (d, 1H, COCH = C**H**, *J* = 16.0 Hz), 8.46 (d, 1H, Arm. H, *J* = 8.0 Hz), 8.94 (s, 1H, 4-H of coumarin ring). ^13^C NMR (125 MHz, CDCl_3_-*d*) δ: 24.00, 116.41, 119.42, 120.74, 123.08, 124.52, 124.70, 125.38, 125.46, 126.39, 127.20, 128.10, 128.86, 129.11, 131.35, 131.50, 131.55, 132.50, 133.58, 133.70, 136.74, 143.32, 143.42, 151.86, 154.00, 157.01, 160.26, 194.18. Anal. calcd for C_28_H_19_NO_3_: C, 80.56; H, 4.59; N, 3.36. Found: C, 80.70; H, 4.63; N, 3.33.

##### 4.1.3.12. 3-(6-Methyl-5-[(2E)-3-(pyridin-2-yl)prop-2-enoyl]pyridin-2-yl)-2H-chromen-2-one (5l)

A yellow powder, yield: 80%. Mp: 188–190 °C. IR (*ν*_max_/cm^−1^): 3067 (CH-arom.), 2978, 2921 (CH-aliph.), 1725, 1663 (2 C = O). ^1^H NMR (500 MHz, CDCl_3_-*d*) δ: 2.77 (s, 3H, CH_3_), 7.29 (t, 1H, Arom. H, *J* = 8.0 Hz), 7.32 (q, 1H, Arom. H, *J* = 8.0 Hz), 7.40 (d, 1H, Arom. H, *J* = 8.0 Hz), 7.49 (d, 1H, Arom. H, *J* = 8.0 Hz), 7.54 (d, 1H, COC**H**=CH, *J* = 16.0 Hz), 7.58 (d, 1H, Arom. H, *J* = 8.0 Hz), 7.69 (d, 1H, Arom. H, *J* = 8.0 Hz), 7.70 (d, 1H, COCH = C**H**, *J* = 16.0 Hz), 7.75 (t, 1H, Arom. H, *J* = 8.0 Hz), 8.01 (d, 1H, Arom. H, *J* = 8.0 Hz), 8.41 (d, 1H, Arom. H, *J* = 8.0 Hz), 8.68 (d, 1H, Arom. H, *J* = 4.0 Hz), 8.91 (s, 1H, 4-H coumarin ring). ^13^C NMR (125 MHz, CDCl_3_-*d*) δ: 24.02, 116.40, 119.42, 120.69, 124.69, 124.52, 124.68, 124.71, 125.15, 129.00, 129.11, 132.48, 133.14, 136.92, 136.96, 143.43, 144.54, 150.31, 151.94, 152.73, 154.01, 157.18, 194.19. Anal. calcd for C_23_H_16_N_2_O_3_: C, 74.99; H, 4.38; N, 7.60. Found: C, 75.21; H, 4.40; N, 7.54.

##### 4.1.3.13. 3-(6-Methyl-5-[(2E)-3-(thiophen-2-yl)prop-2-enoyl]pyridin-2-yl)-2H-chromen-2-one (5m)

A yellow powder, yield: 71%. Mp: 197–199 °C. IR (*ν*_max_/cm^−1^): 3055 (CH-arom.), 3001, 2930 (CH-aliph.), 1722, 1659 (2 C = O). ^1^H NMR (500 MHz, CDCl_3_-*d*) δ: 2.73 (s, 3H, CH_3_), 6.95 (d, 1H, COC**H**=CH, *J* = 16.0 Hz), 7.09 (t, 1H, Arm. H, *J* = 4.0 Hz), 7.33–7.40 (m, 3H, Arm. H), 7.47 (d, 1H, Arm. H, *J* = 4.0 Hz), 7.59 (t, 1H, Arm. H, *J* = 8.0 Hz), 7.65 (d, 1H, COCH = C**H**, *J* = 16.0 Hz), 7.69 (d, 1H, Arm. H, *J* = 8.0 Hz), 7.88 (d, 1H, Arm. H, *J* = 8.0 Hz), 8.39 (d, 1H, Arm. H, *J* = 8.0 Hz), 8.89 (s, 1H, 4-H of coumarin ring). ^13^C NMR (125 MHz, CDCl_3_-*d*) δ: 23.72, 116.37, 119.39, 120.64, 124.56, 124.66, 128.50, 129.06, 129.83, 132.43, 132.55, 133.48, 136.45, 137.85, 138.99, 139.62, 143.29, 151.70, 153.95, 156.75, 160.23, 193.96. Anal. calcd for C_22_H_15_NO_3_S: C, 70.76; H, 4.05; N, 3.75. Found: C, 70.89; H, 4.02; N, 3.76.

##### 4.1.3.14. 3-(6-Methyl-5-[(2E)-3–(5-methylfuran-2-yl)prop-2-enoyl]pyridin-2-yl)-2H-chromen-2-one (5n)

A yellow powder, yield: 58%. Mp: 188–190 °C. ^1^H NMR (500 MHz, CDCl_3_-*d*) δ: 2.38 (s, 3H, CH_3_), 2.74 (s, 3H, CH_3_), 6.14 (s, 1H, Arm. H), 6.64 (s, 1H, Arm. H), 6.96 (d, 1H, COC**H**=CH, *J* = 16.0 Hz), 7.26 (d, 1H, COCH = C**H**, *J* = 16.0 Hz), 7.34 (t, 1H, Arom. H, *J* = 8.0 Hz), 7.40 (d, 1H, Arom. H, *J* = 8.0 Hz), 7.59 (t, 1H, Arom. H, *J* = 8.0 Hz), 7.70 (d, 1H, Arom. H, *J* = 8.0 Hz), 7.90 (d, 1H, Arom. H, *J* = 8.0 Hz), 8.37 (d, 1H, Arom. H, *J* = 8.0 Hz), 8.89 (s, 1H, 4-H of coumarin ring). ^13^C NMR (125 MHz, CDCl_3_-*d*) δ: 14.04, 23.63, 109.67, 116.40, 119.28, 119.41, 120.80, 121.32, 124.70, 129.11, 129.24, 132.49, 133.97, 136.58, 137.74, 143.39, 149.55, 151.47, 153.97, 156.71, 156.83, 160.27, 193.92. Anal. calcd for C_23_H_17_NO_4_: C, 74.38; H, 4.61; N, 3.77. Found: C, 74.51; H, 4.61; N, 3.80.

##### 4.1.3.15. 3-(6-Methyl-5-[(2E)-3–(3-methyl-1-phenyl-1H-pyrazol-4-yl)prop-2-enoyl]pyridin-2-yl)-2H-chromen-2-one (5o)

A yellow powder, yield: 61%. Mp: 204–206 °C. ^1^H NMR (500 MHz, CDCl_3_-*d*) δ: 2.48 (s, 3H, CH_3_), 2.75 (s, 3H, CH_3_), 6.94 (d, 1H, COC**H**=CH, *J* = 16.0 Hz), 7.30–7.35 (m, 2H, Arm. H), 7.40 (d, 1H, Arm. H, *J* = 8.0 Hz), 7.46 (t, 2H, Arm. H, *J* = 8.0 Hz), 7.52 (d, 1H, COCH = C**H**, *J* = 16.0 Hz), 7.59 (t, 1H, Arm. H, *J* = 8.0 Hz), 7.67 (d, 2H, Arm. H, *J* = 8.0 Hz), 7.70 (d, 1H, Arm. H, *J* = 8.0 Hz), 7.88 (d, 1H, Arm. H, *J* = 8.0 Hz), 8.14 (s, 1H, Arm. H), 8.39 (d, 1H, Arm. H, *J* = 8.0 Hz), 8.90 (s, 1H, 4-H of coumarin ring). ^13^C NMR (125 MHz, CDCl_3_-*d*) δ: 13.31, 23.76, 116.40, 118.12, 119.11 (2 C), 119.43, 120.67, 124.15, 124.62, 124.69, 127.08, 127.79, 129.07, 129.56 (2 C), 132.44, 133.80, 136.40, 137.20, 139.20, 143.27, 150.91, 151.61, 153.98, 156.69, 160.28, 194.39. Anal. calcd for C_28_H_21_N_3_O_3_: C, 75.15; H, 4.73; N, 9.39. Found: C, 75.32; H, 4.75; N, 9.44.

### 4.2. Biological evaluation

#### 4.2.1. Carbonic anhydrase isoforms inhibition assay

The CA inhibition activity for the herein reported **MPC** derivatives was evaluated against the *h*CA isoforms I, II, IX, and XII using stopped-flow CO_2_ hydrase test^7^^,^^33–36^ (see the Supplementary Material).

#### 4.2.2. *In vitro* antitumor screening against 59 cancer cell lines

The anticancer test was conducted using the methods of the Drug Evaluation Branch, National Cancer Institute, Bethesda, MD, using 59 human tumour cell lines derived from nine human tissues. The GI_50_, TGI, and LC_50_ dose-response parameters were calculated for each medication^37–39^.

## Supplementary Material

Supplemental Material
