# Supplementary material for: Novel 3-(6-methylpyridin-2-yl)coumarin-based chalcones as selective inhibitors of cancer-related carbonic anhydrases IX and XII endowed with anti-proliferative activity
Source: J Enzyme Inhib Med Chem. 2022 Apr 19;37(1):1043–52. doi: 10.1080/14756366.2022.2056734 (PMC9037210; doi:10.1080/14756366.2022.2056734)
Supplement: Supplemental Material [file IENZ_A_2056734_SM7917.pdf]

Supplementary Material

Novel 3-(6-methylpyridin-2-yl)coumarin-based Chalcones as Selective Inhibitors of Cancer-related Carbonic Anhydrases IX and XII endowed with Anti-Proliferative Activity

Haytham O. Tawfik, Moataz A. Shaldam, Alessio Nocentini, Rofaida Salem, Hadia Almahli, Sara T. Al-Rashood, Claudiu T. Supuran*, Wagdy M. Eldehna*

Spectral data section ……………………………..…. P.02-P.27

Biological section ………………………………..…. P.28-P.47

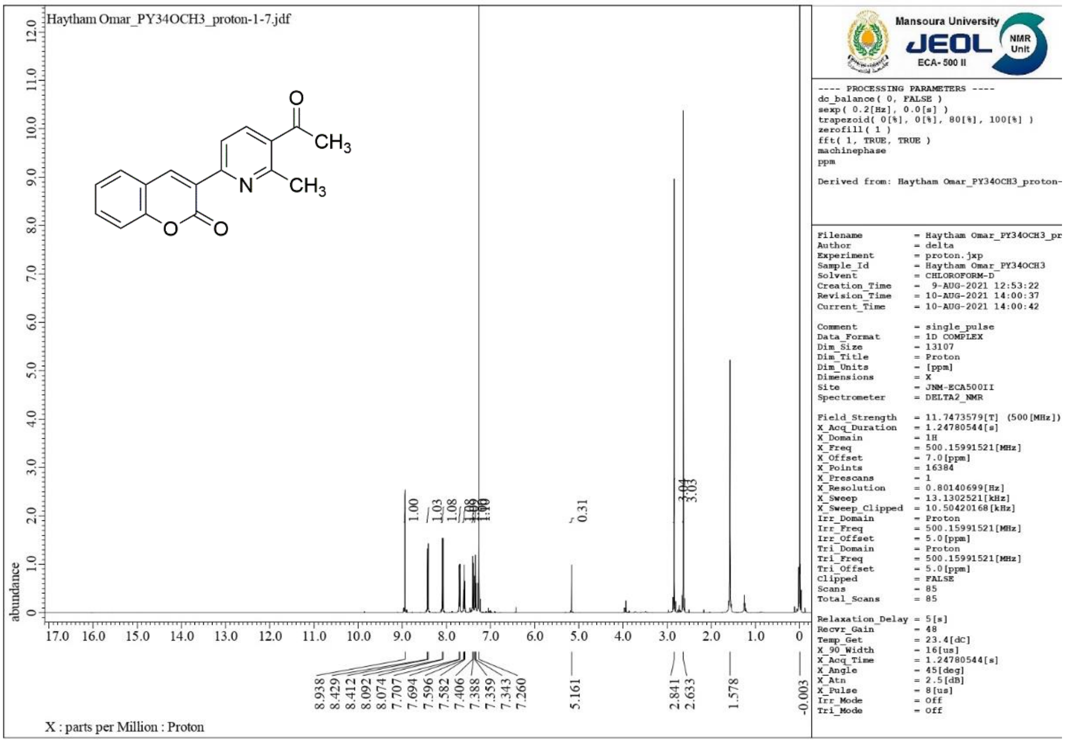

Figure S1. ^{1}H NMR (500 MHz, $CDCl_3$-*d*) spectrum of compound **3**

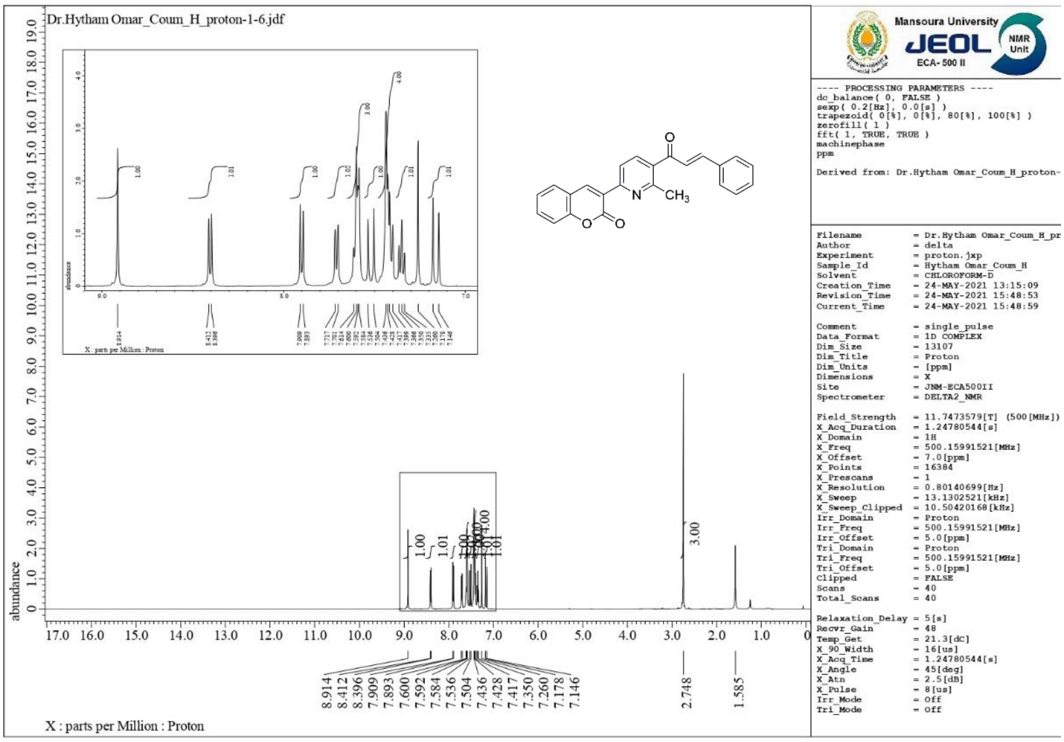

Figure S2. ^{1}H NMR (500 MHz, $CDCl_3$-*d*) spectrum of compound **5a**

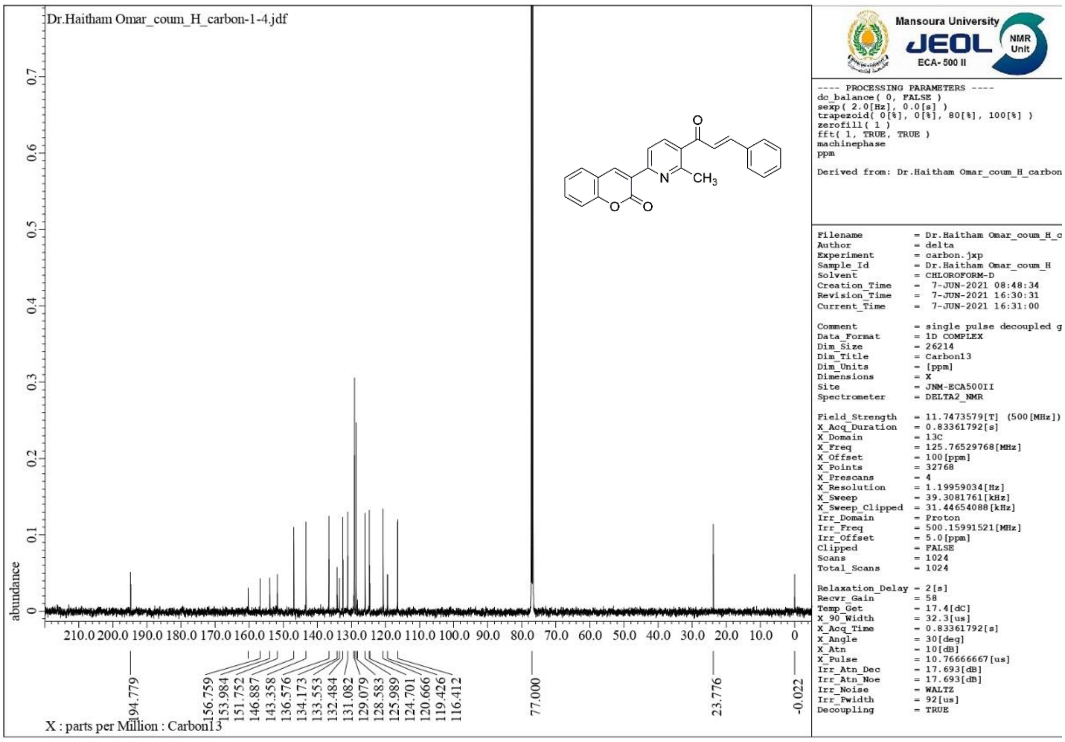

Figure S3. ^{13}C NMR (125 MHz, $CDCl_3$-*d*) spectrum of compound **5a**

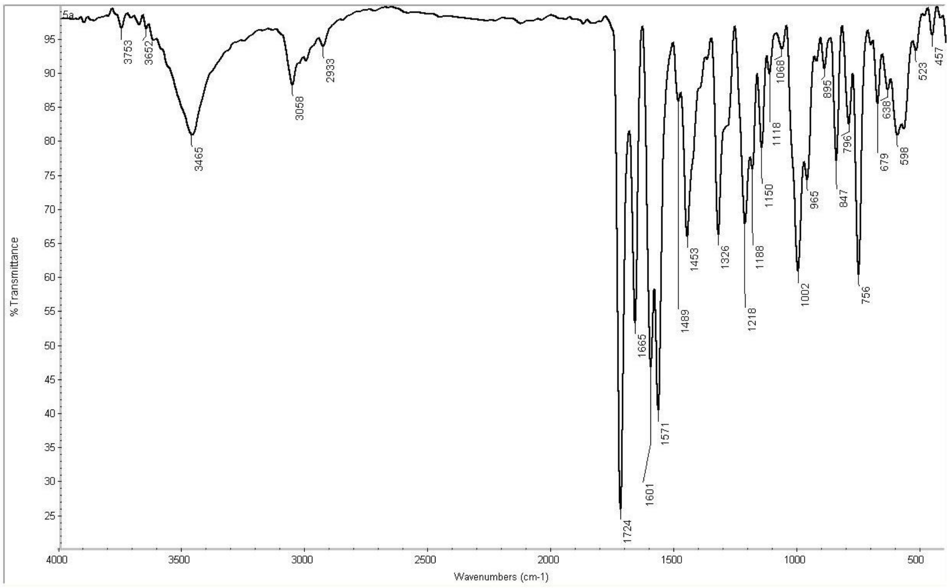

Figure S4. IR spectrum of compound **5a**

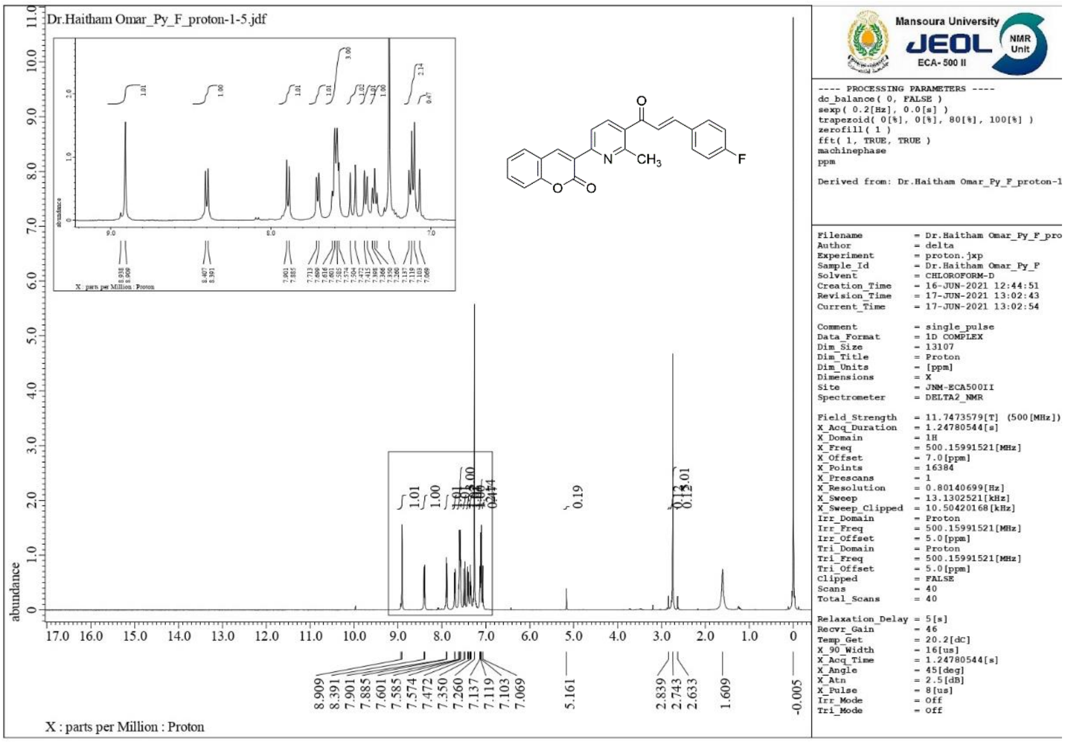

Figure S5. ^{1}H NMR (500 MHz, $CDCl_3$-*d*) spectrum of compound **5b**

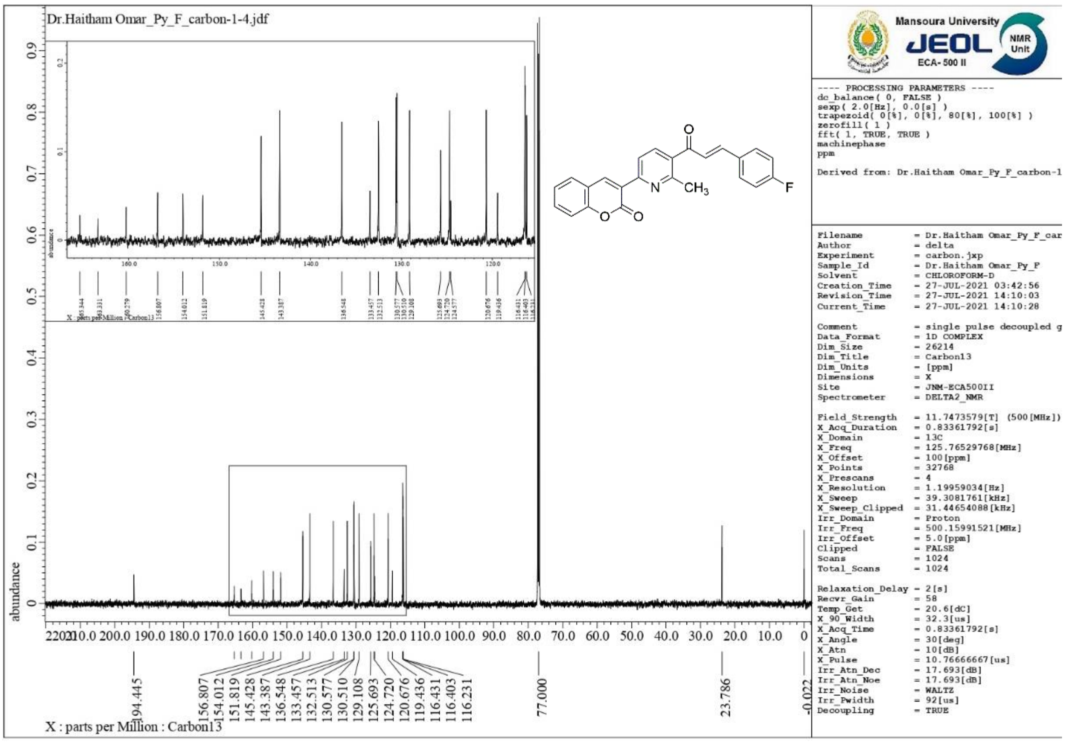

Figure S6. ^{13}C NMR (125 MHz, $CDCl_3$-*d*) spectrum of compound **5b**

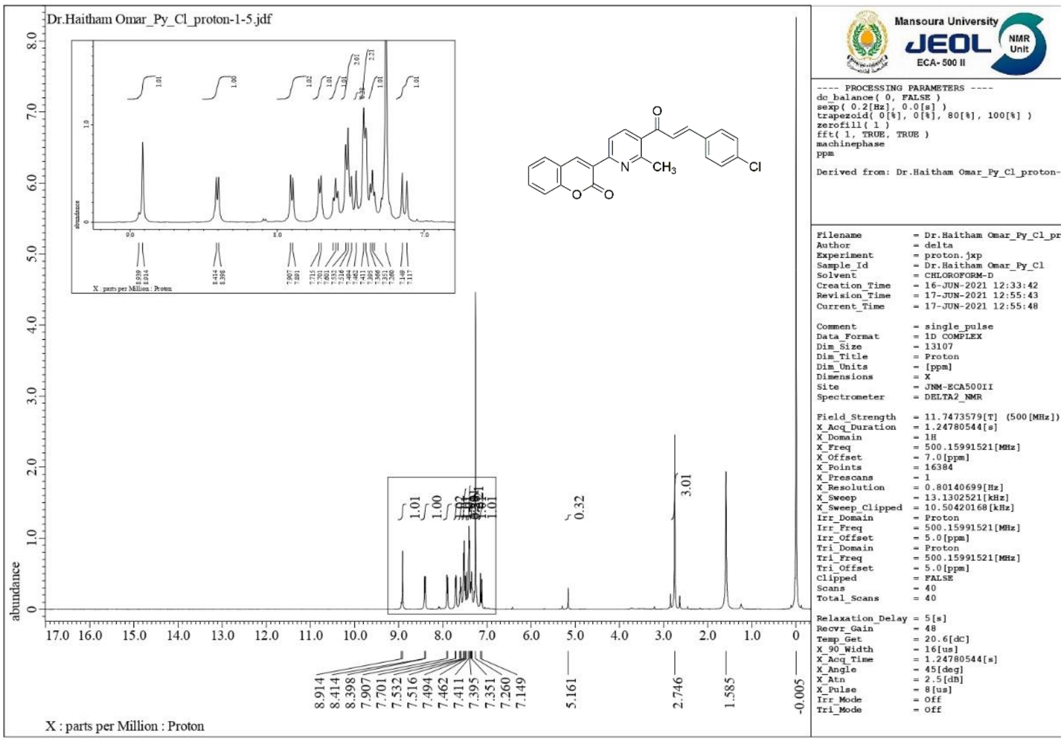

Figure S7. ^{1}H NMR (500 MHz, $CDCl_3$-*d*) spectrum of compound **5c**

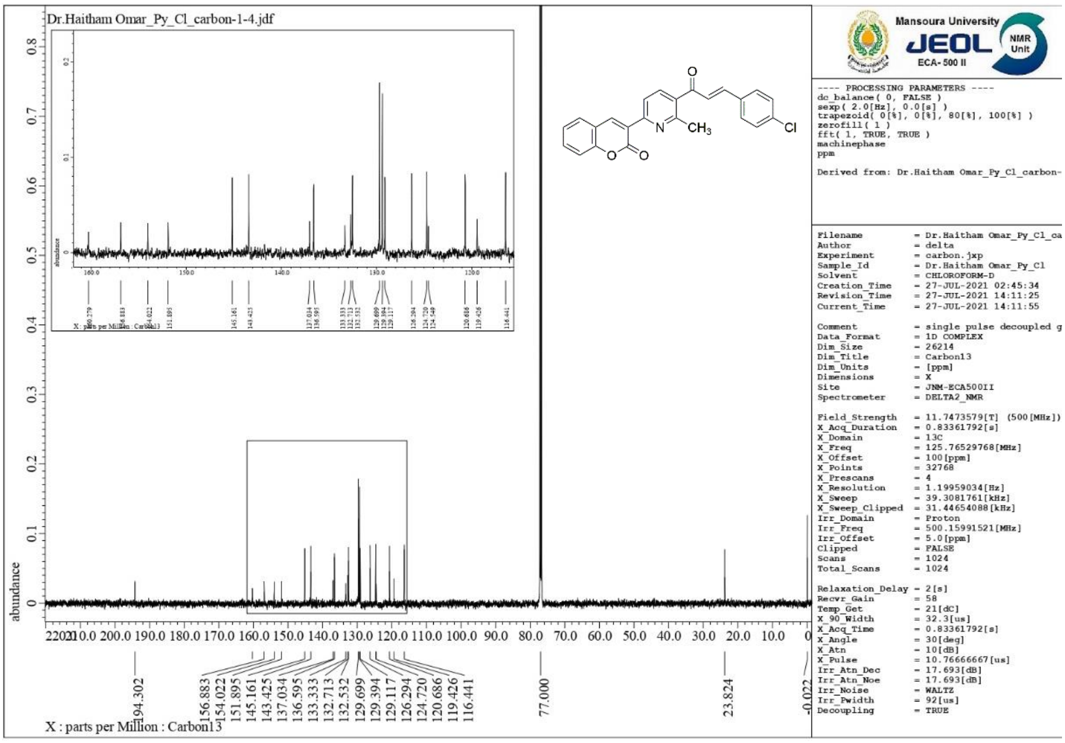

Figure S8. ^{13}C NMR (125 MHz, $CDCl_3$-*d*) spectrum of compound **5c**

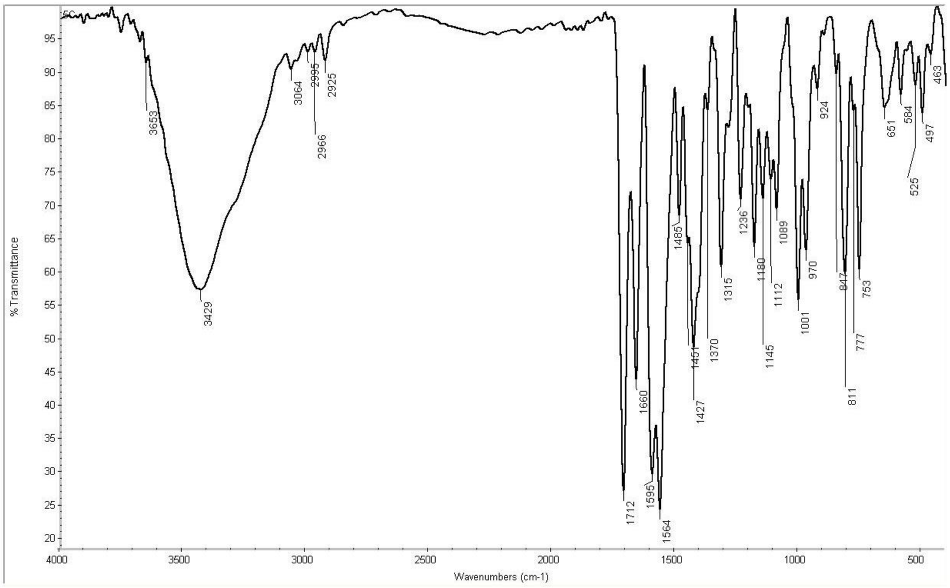

Figure S9. IR spectrum of compound **5c**

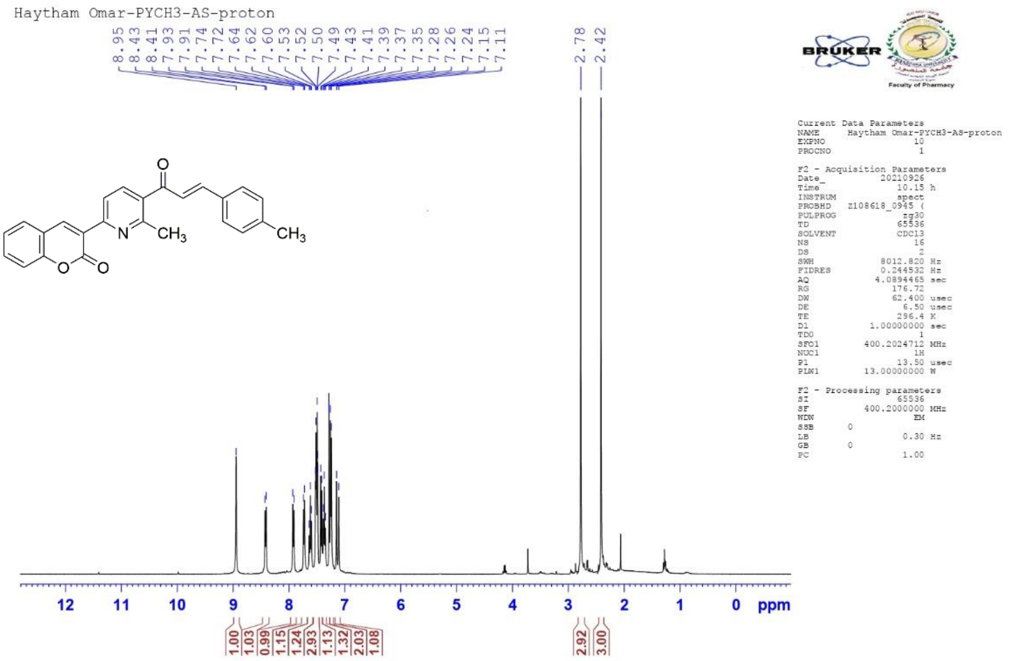

Figure S10. ^{1}H NMR (400 MHz, $CDCl_3$-*d*) spectrum of compound **5d**

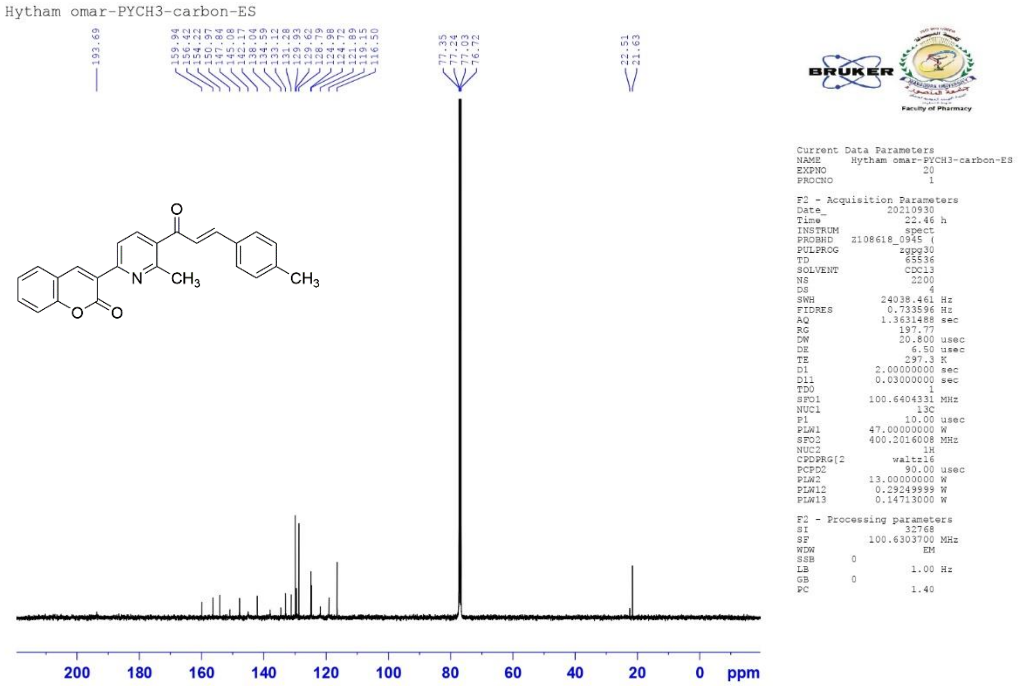

Figure S11. ^{13}C NMR (100 MHz, $CDCl_3$-*d*) spectrum of compound **5d**

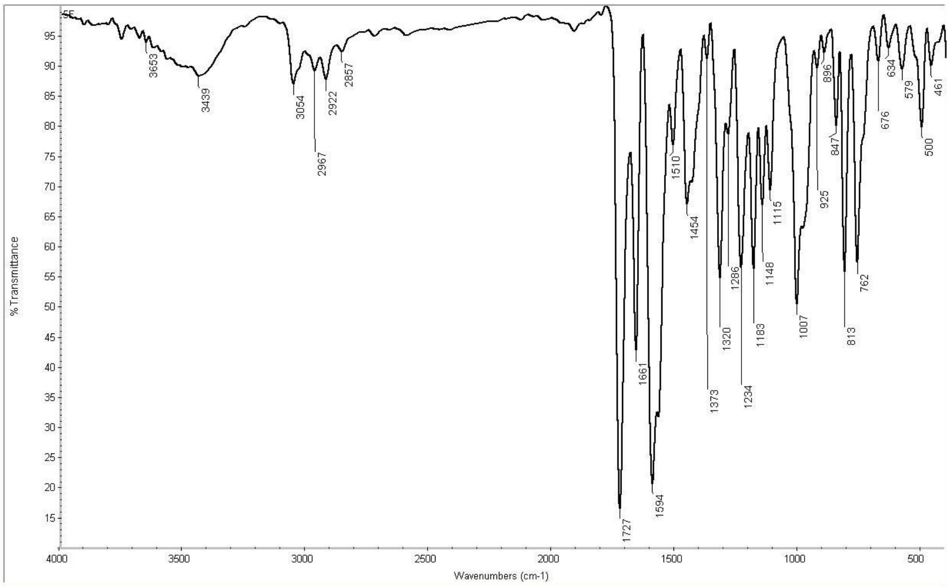

Figure S12. IR spectrum of compound **5d**

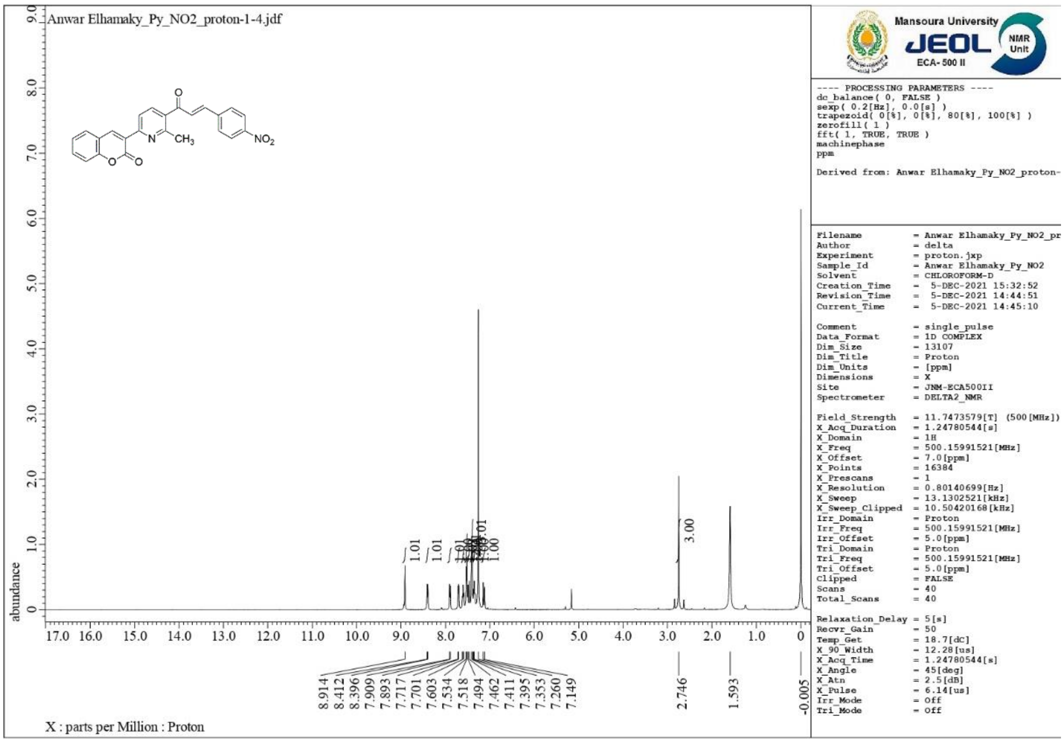

Figure S13. ^{1}H NMR (500 MHz, $CDCl_3$-*d*) spectrum of compound **5e**

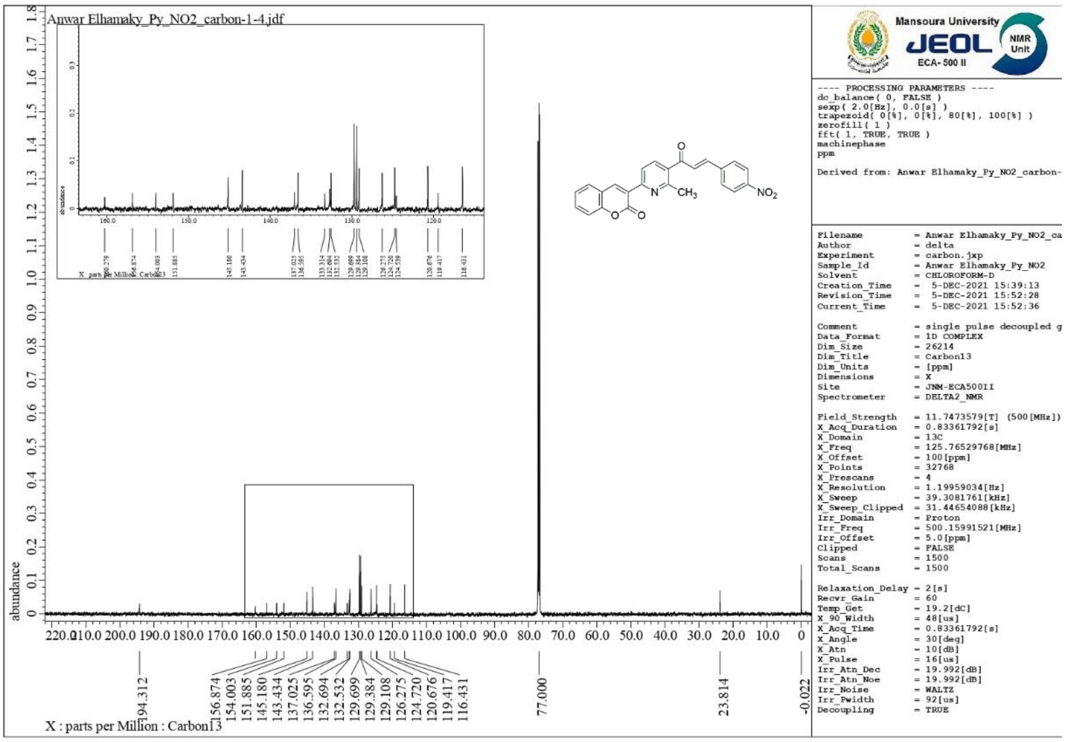

Figure S14. ^{13}C NMR (125 MHz, $CDCl_3$-*d*) spectrum of compound **5e**

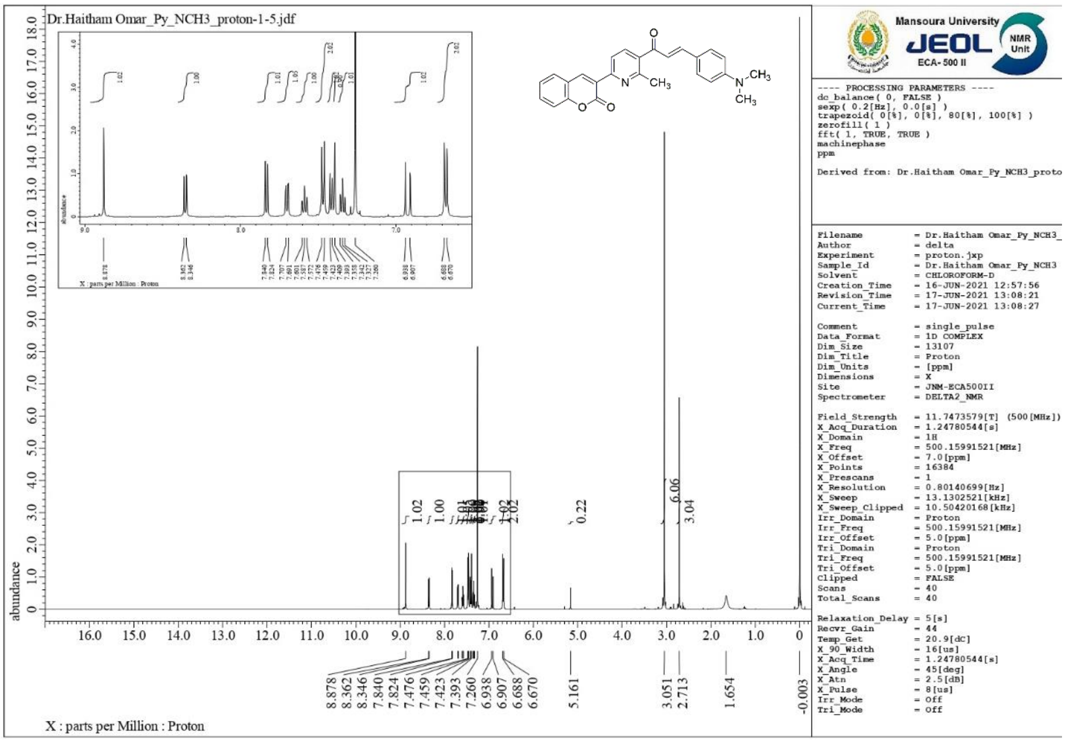

Figure S15. ^{1}H NMR (500 MHz, $CDCl_3$-*d*) spectrum of compound **5f**

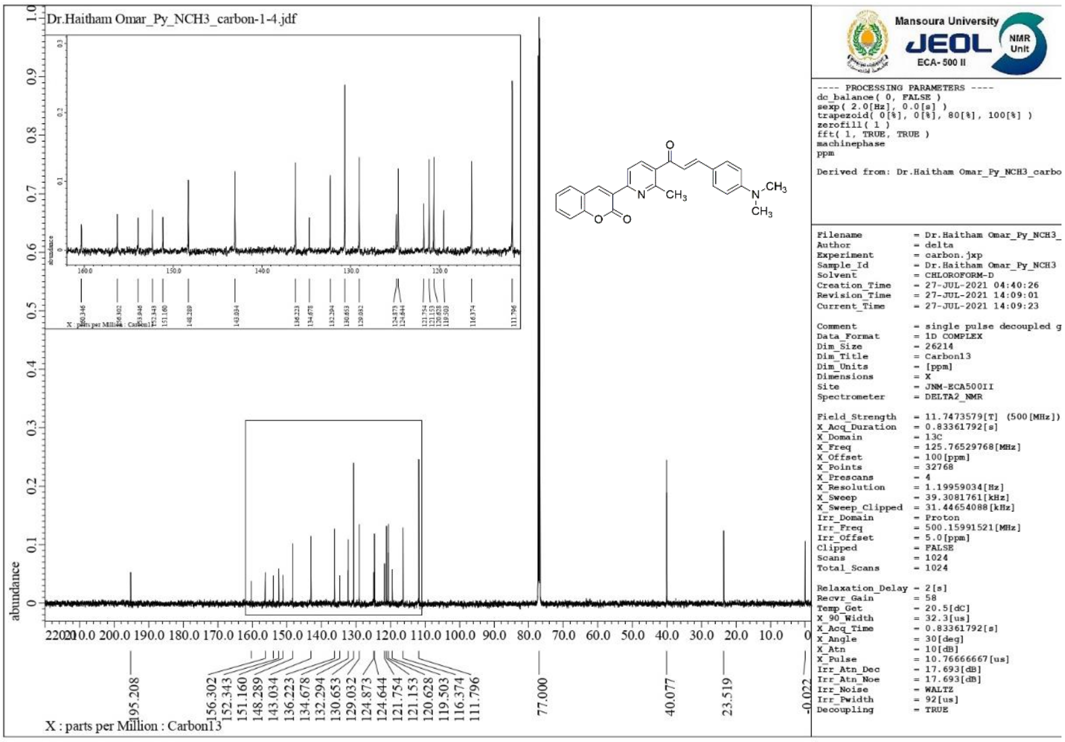

Figure S16. ^{13}C NMR (125 MHz, $CDCl_3$-*d*) spectrum of compound **5f**

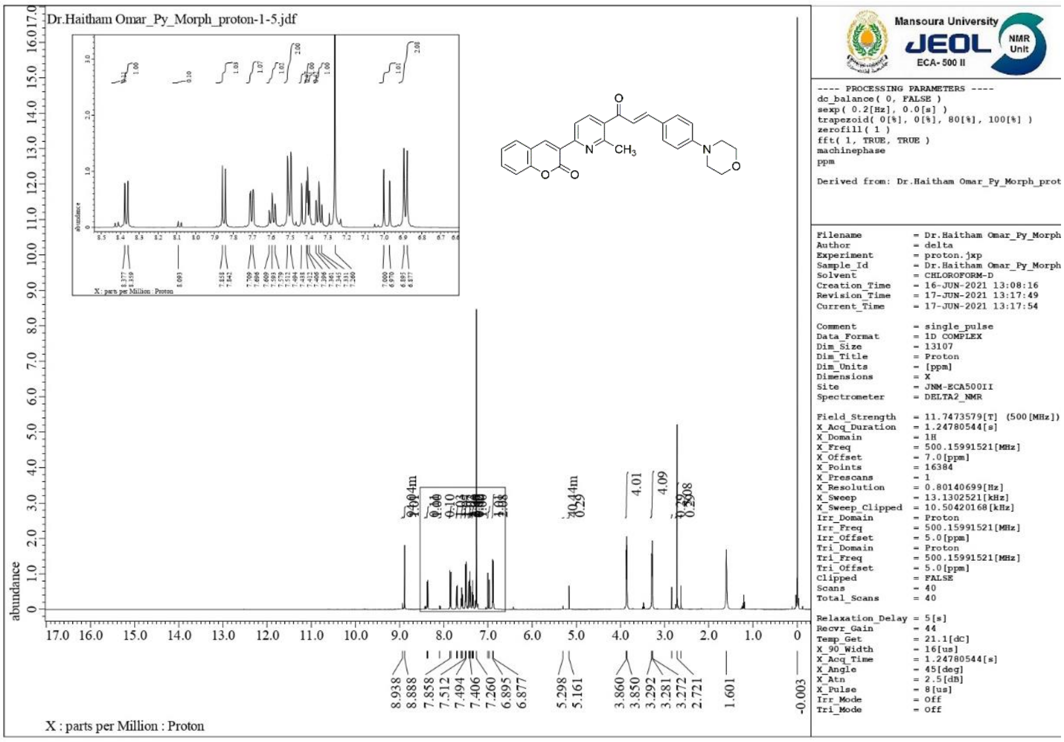

Figure S17. ^{1}H NMR (500 MHz, $CDCl_3$-*d*) spectrum of compound **5g**

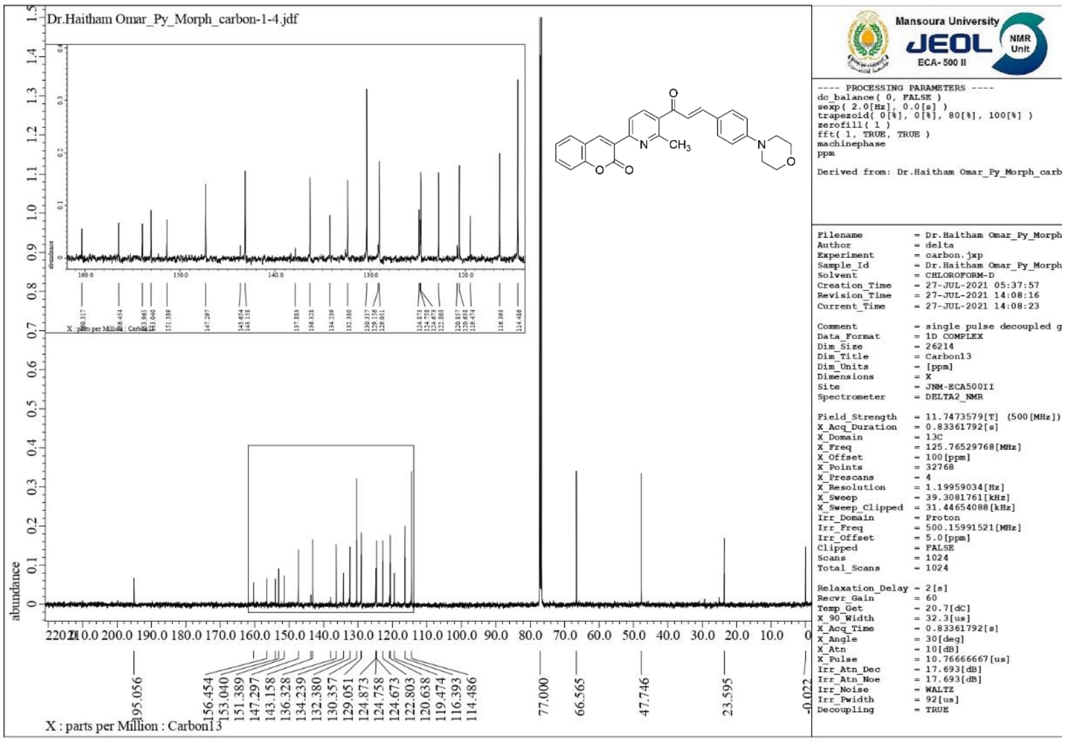

Figure S18. ^{13}C NMR (125 MHz, $CDCl_3$-*d*) spectrum of compound **5g**

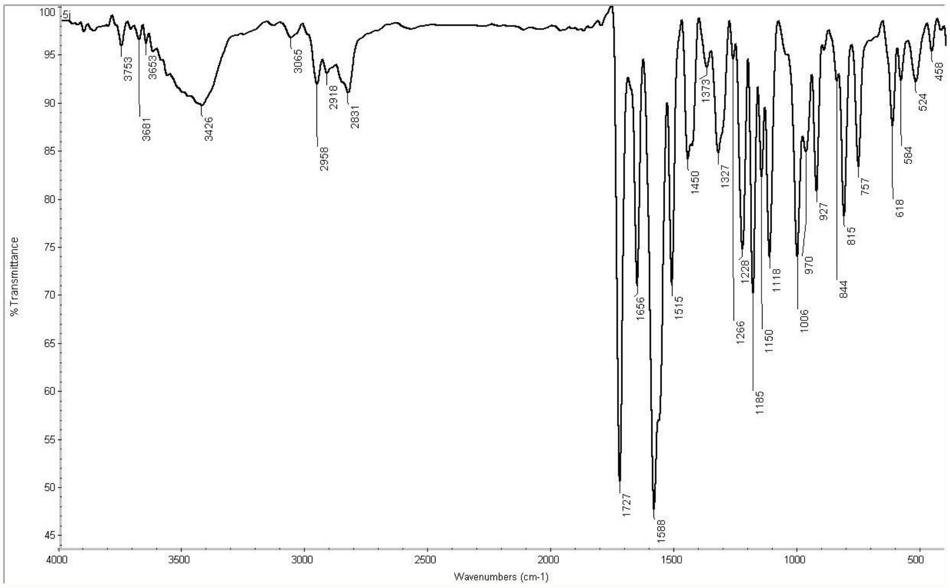

Figure S19. IR spectrum of compound **5g**

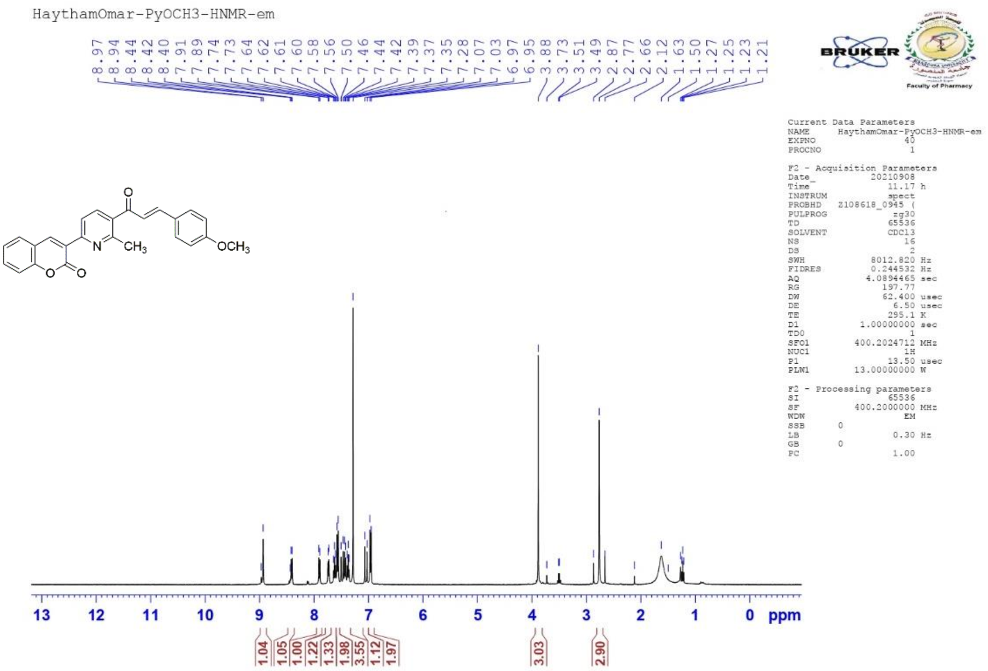

Figure S20. ^{1}H NMR (400 MHz, $CDCl_3$-*d*) spectrum of compound **5h**

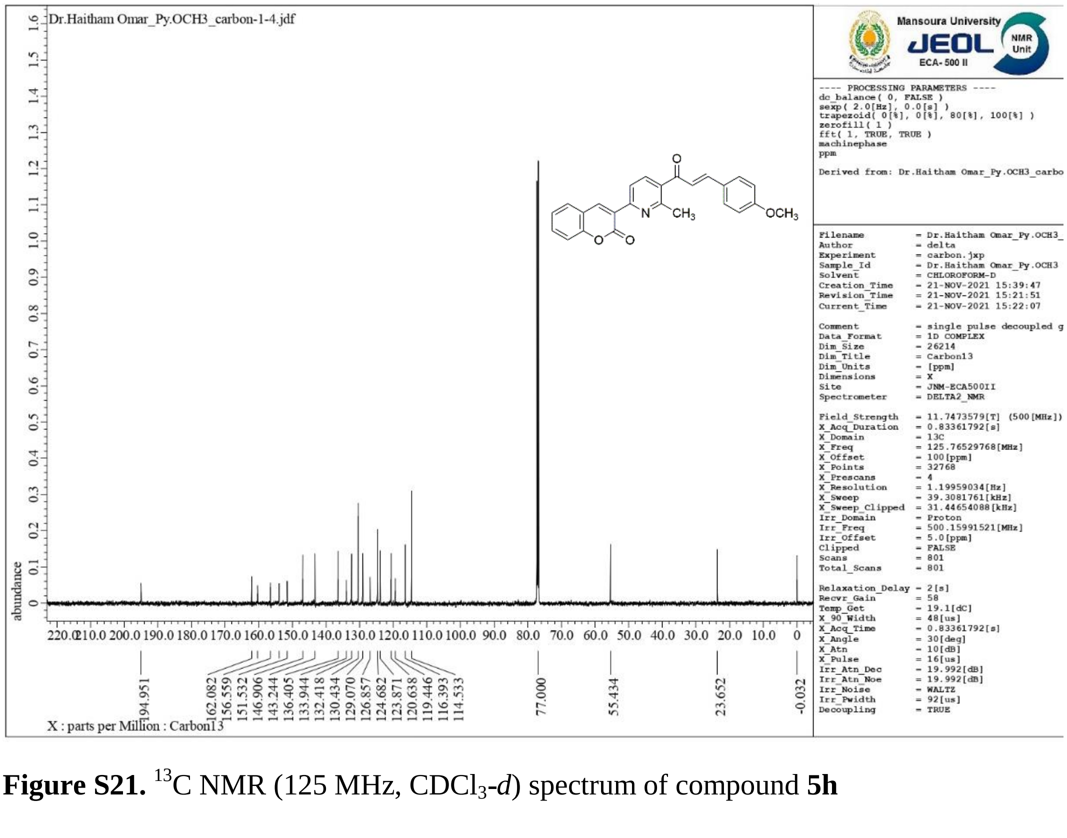

Figure S21. ^{13}C NMR (125 MHz, $CDCl_3$-*d*) spectrum of compound **5h**

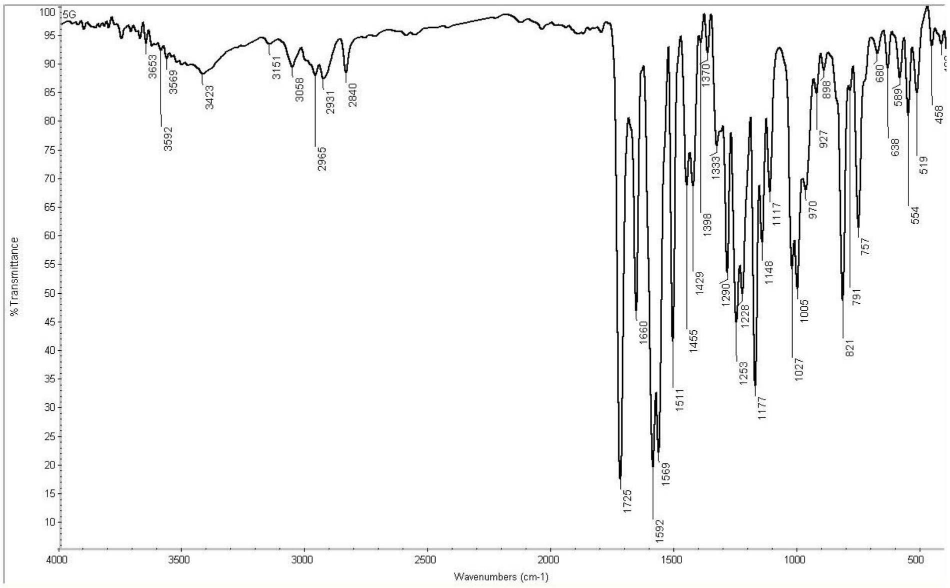

Figure S22. IR spectrum of compound **5h**

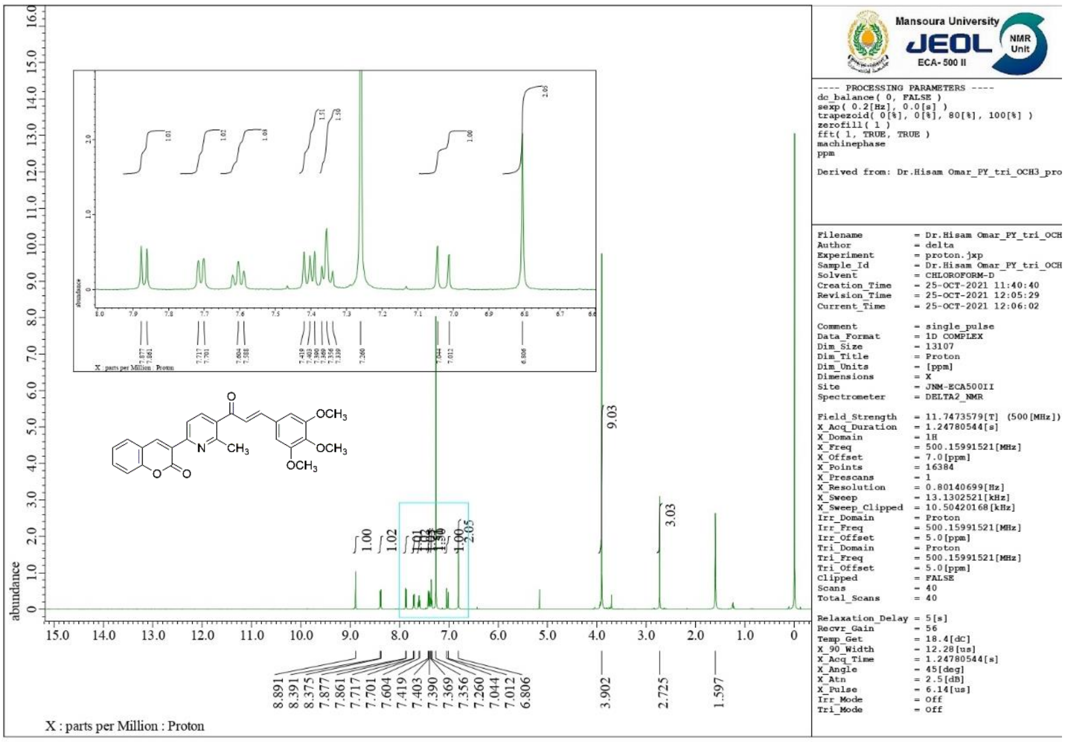

Figure S23. ^{1}H NMR (500 MHz, $CDCl_3$-*d*) spectrum of compound **5i**

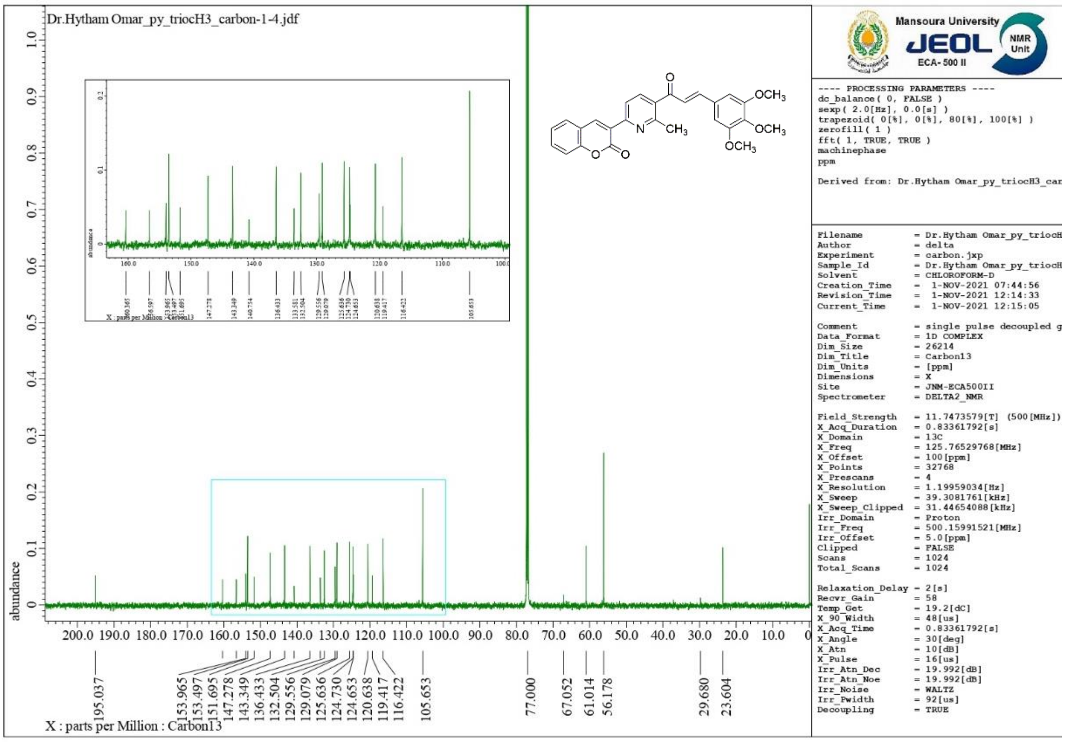

Figure S24. ^{13}C NMR (125 MHz, $CDCl_3$-*d*) spectrum of compound **5i**

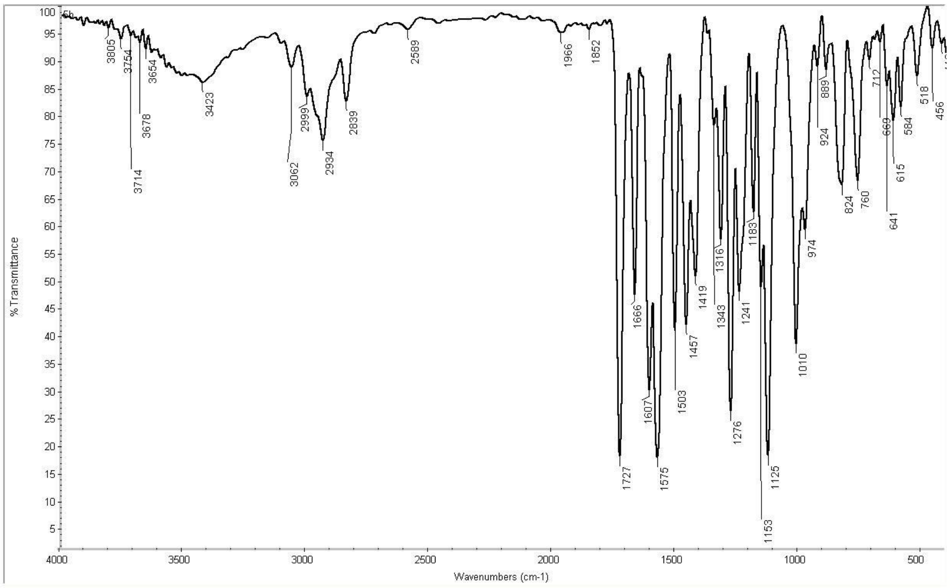

Figure S25. IR spectrum of compound **5i**

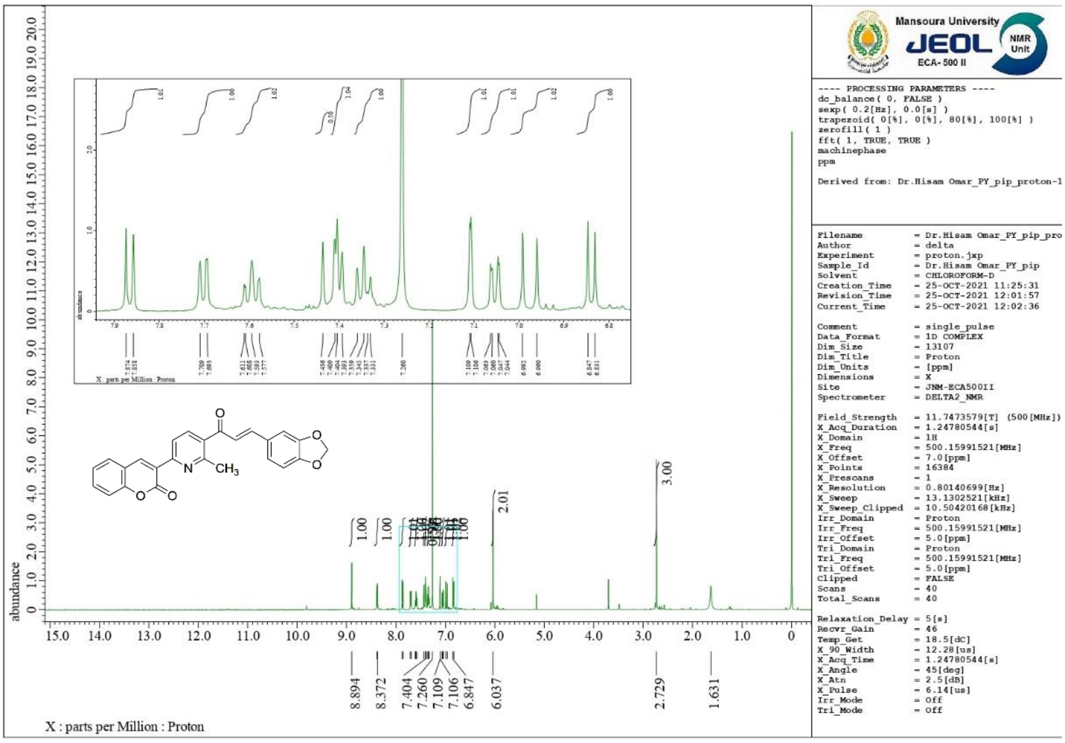

Figure S26. ^{1}H NMR (500 MHz, $CDCl_3$-*d*) spectrum of compound **5j**

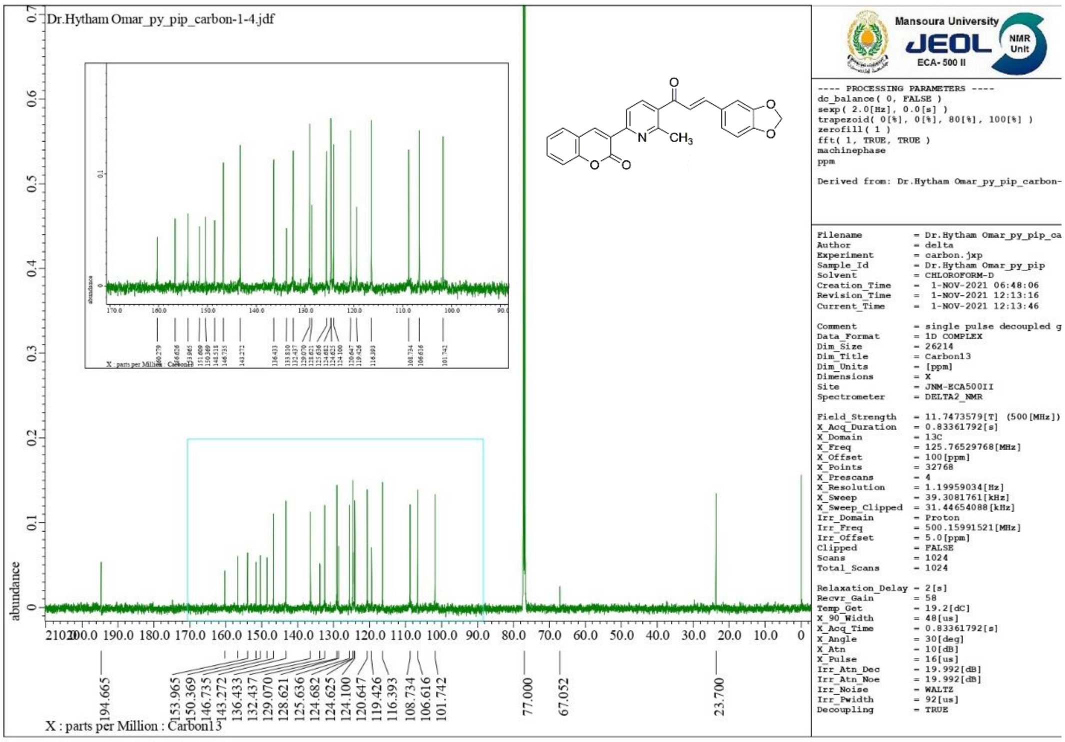

Figure S27. ^{13}C NMR (125 MHz, $CDCl_3$-*d*) spectrum of compound **5j**

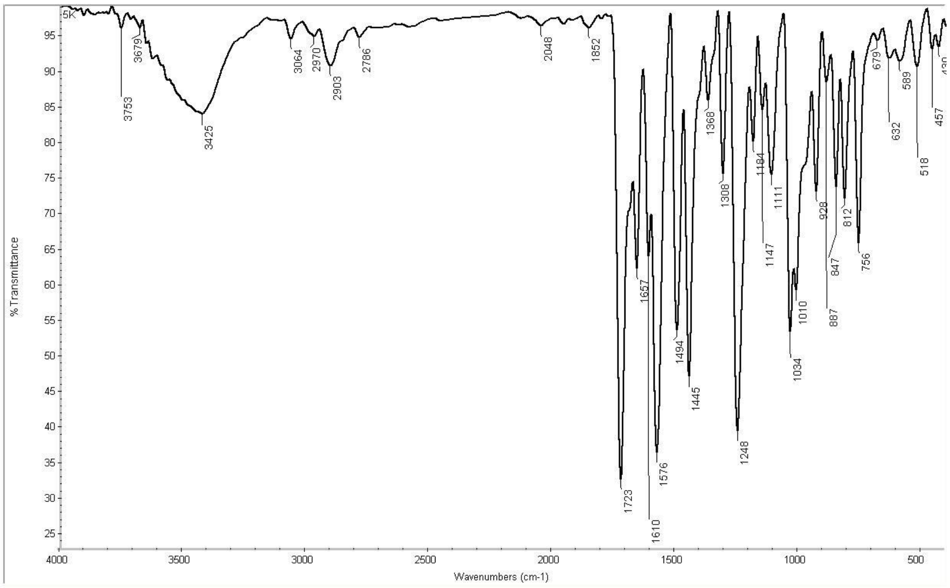

Figure S28. IR spectrum of compound **5j**

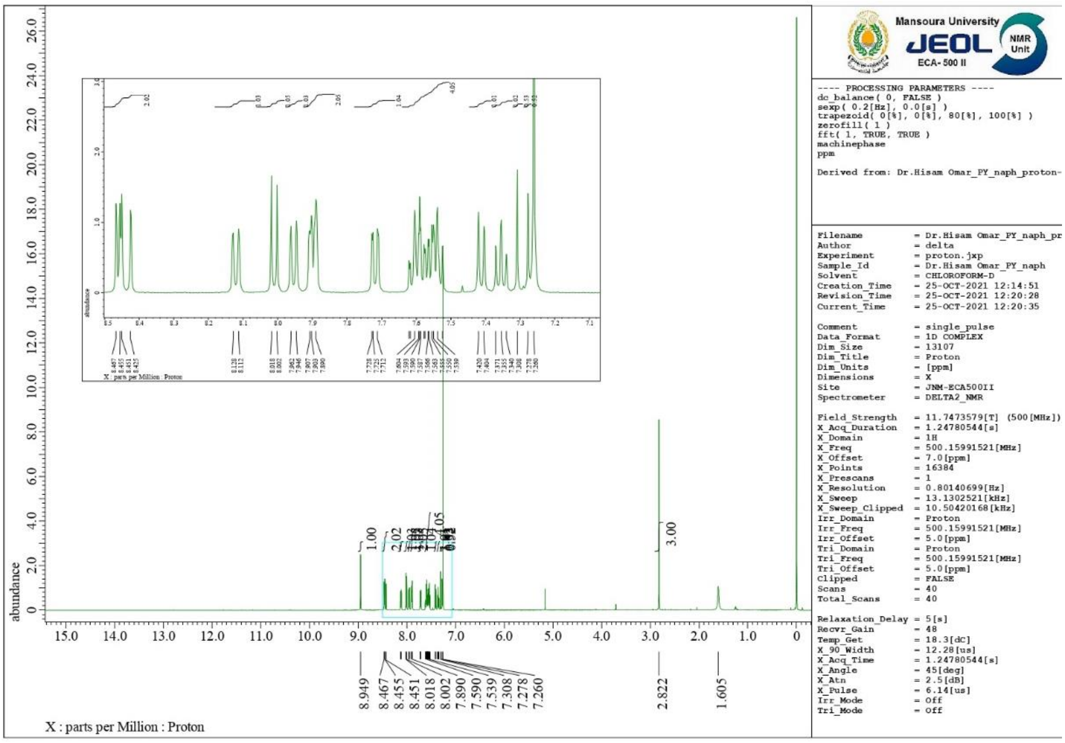

Figure S29. ^{1}H NMR (500 MHz, $CDCl_3$-*d*) spectrum of compound **5k**

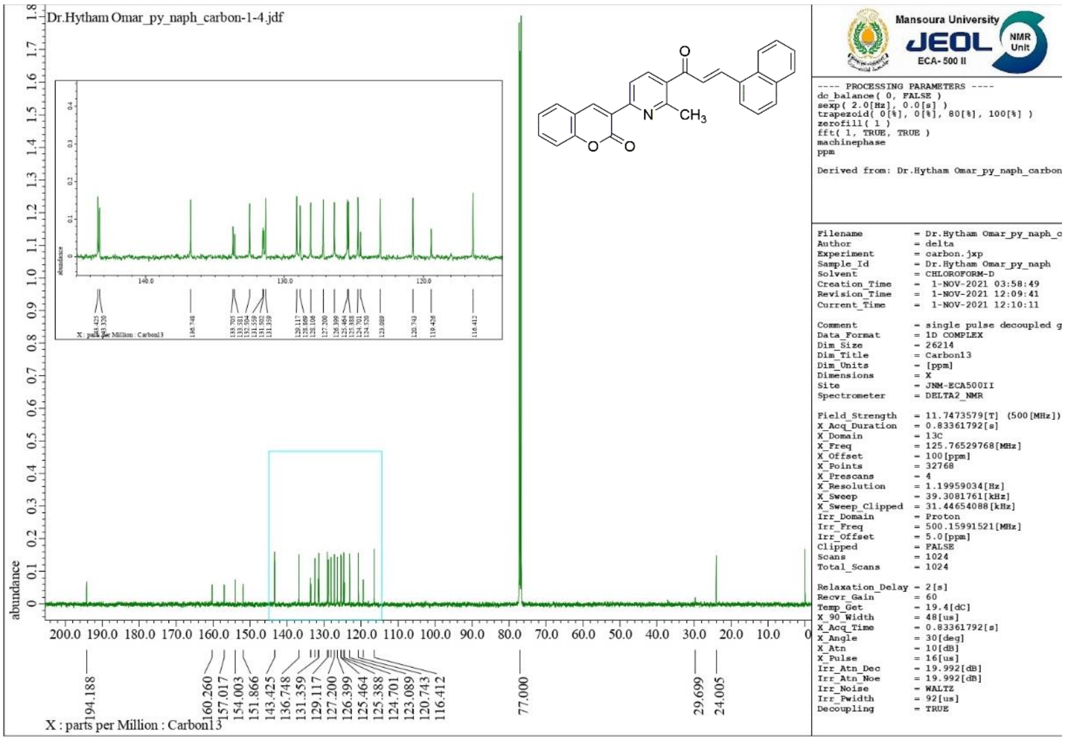

Figure S30. ^{13}C NMR (125 MHz, $CDCl_3$-*d*) spectrum of compound **5k**

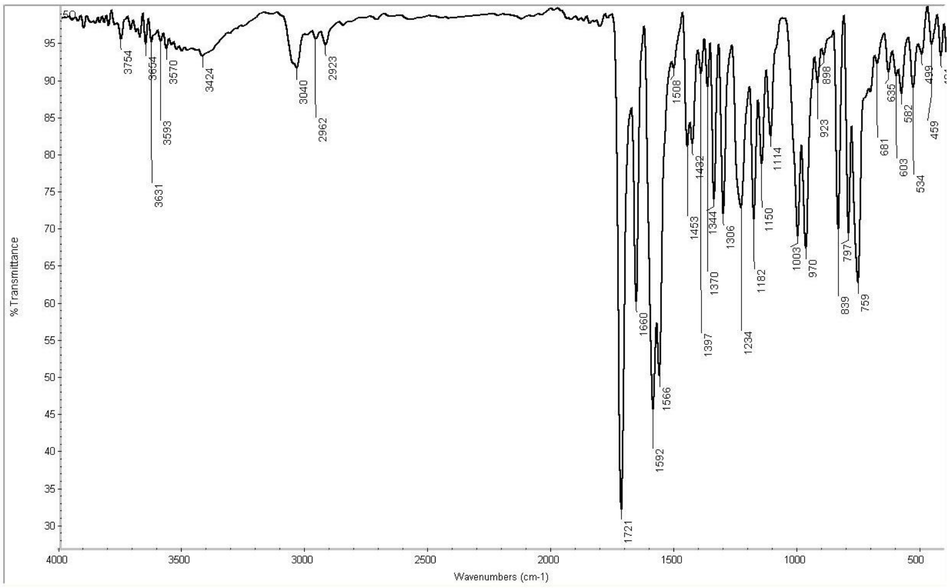

Figure S31. IR spectrum of compound **5k**

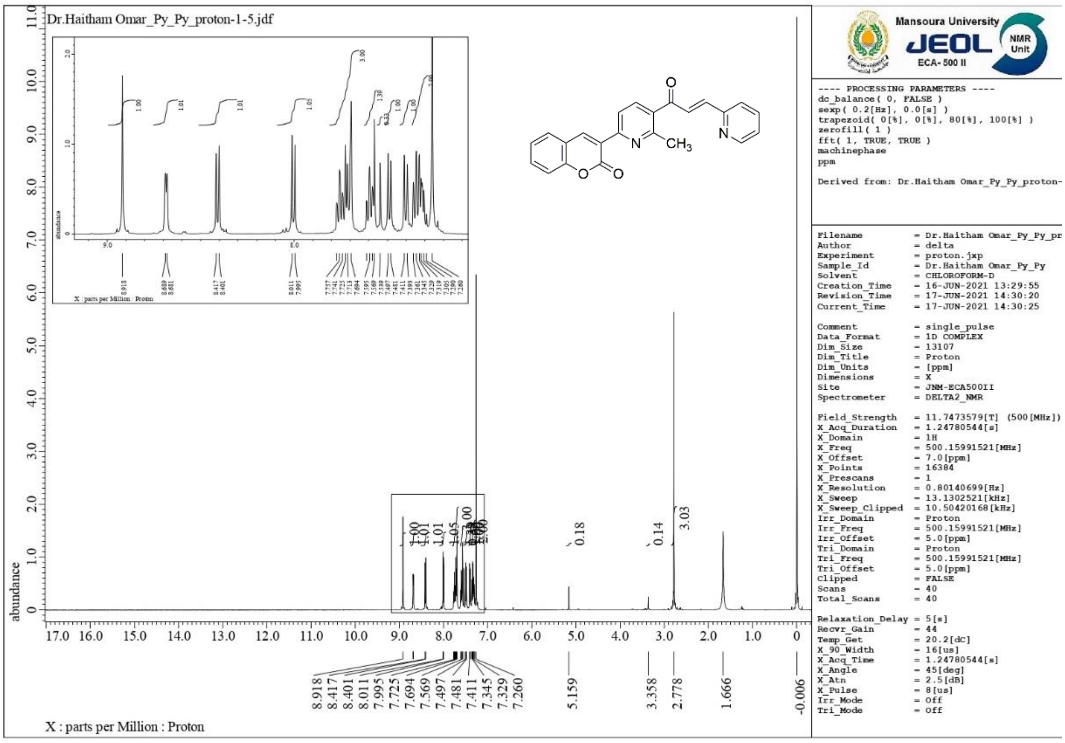

Figure S32. ^{1}H NMR (500 MHz, $CDCl_3$-*d*) spectrum of compound **5l**

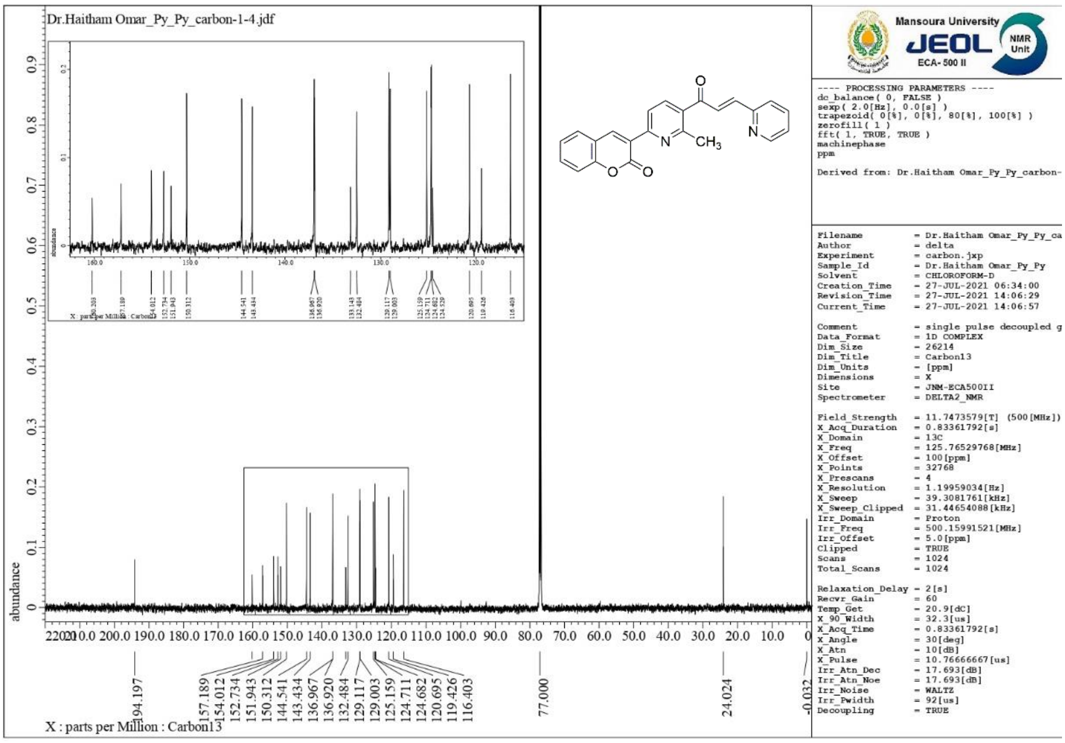

Figure S33. ^{13}C NMR (125 MHz, $CDCl_3$-*d*) spectrum of compound **5l**

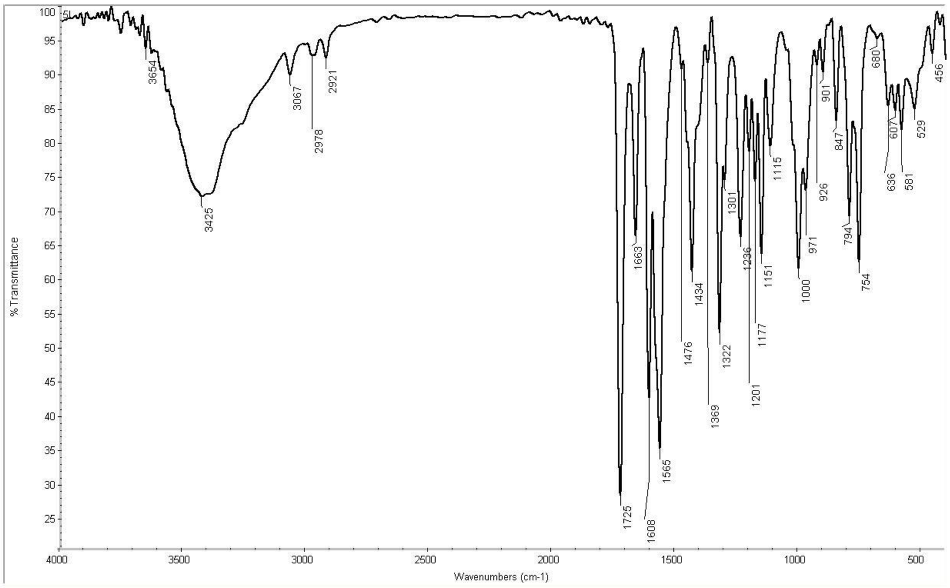

Figure S34. IR spectrum of compound **5l**

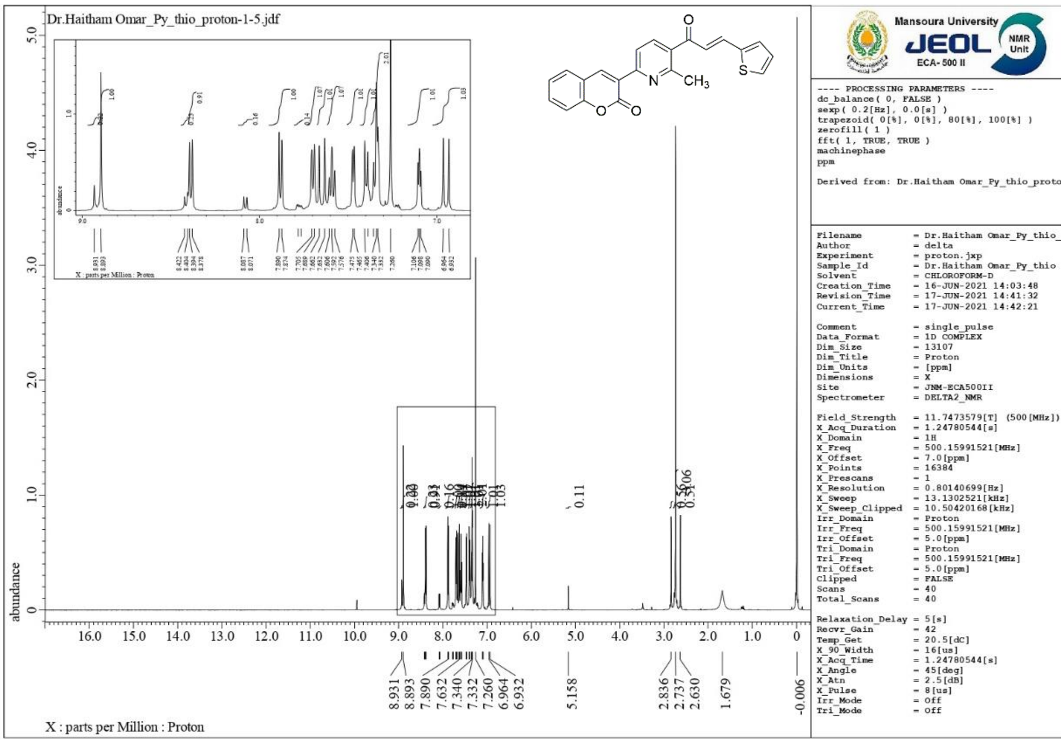

Figure S35. ^{1}H NMR (500 MHz, $CDCl_3$-*d*) spectrum of compound **5m**

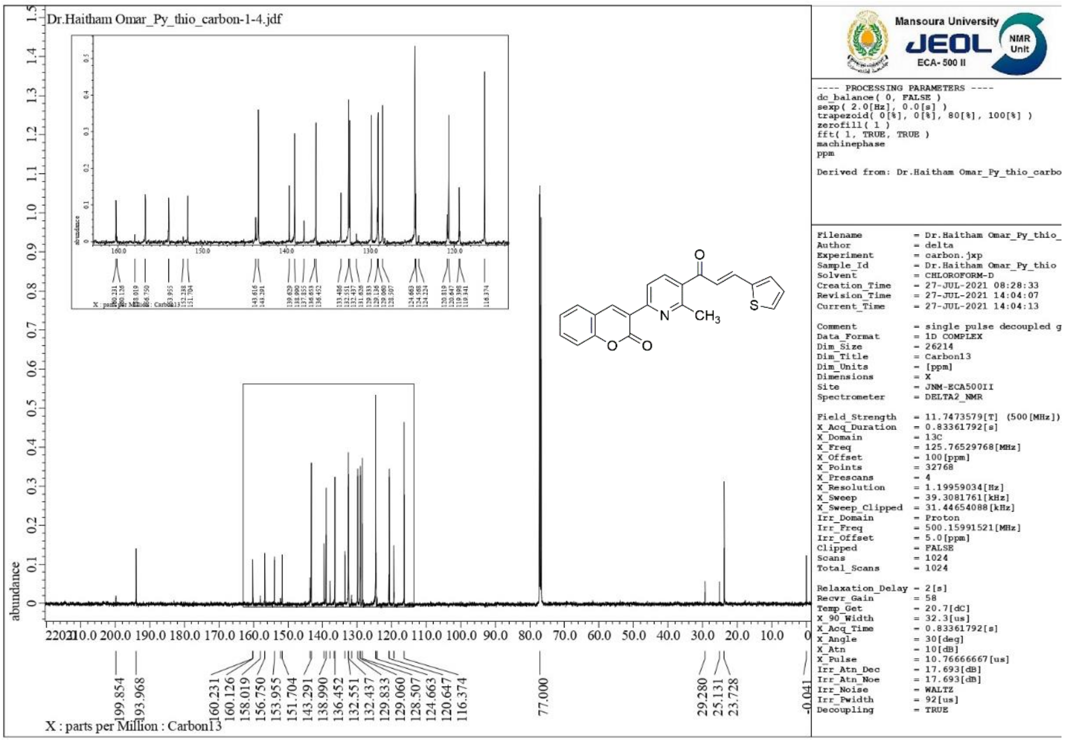

Figure S36. ^{13}C NMR (125 MHz, $CDCl_3$-*d*) spectrum of compound **5m**

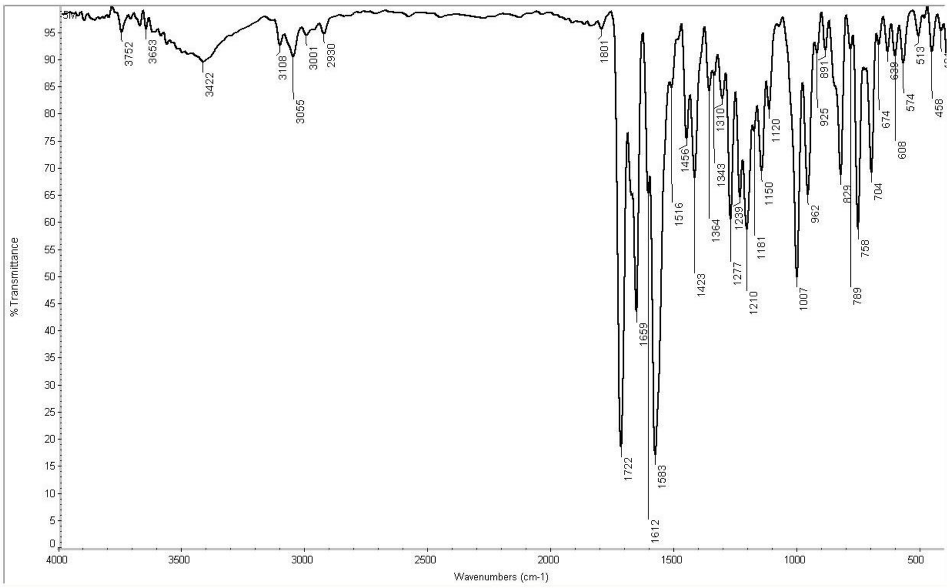

Figure S37. IR spectrum of compound **5m**

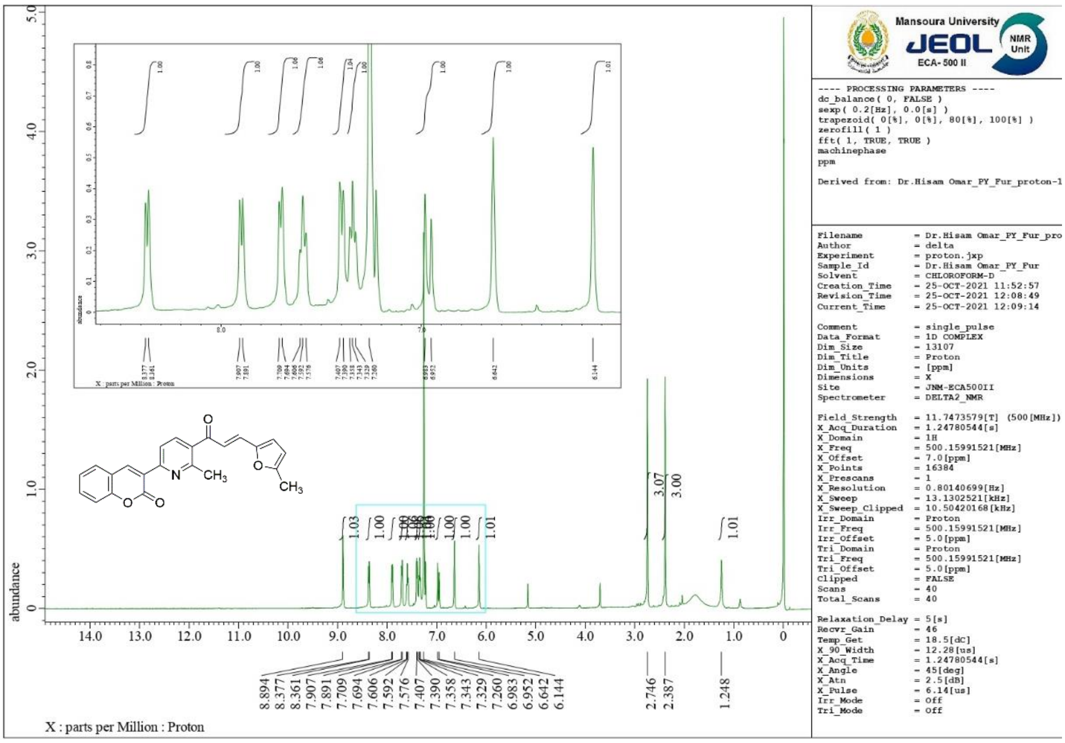

Figure S38. ^{1}H NMR (500 MHz, $CDCl_3$-*d*) spectrum of compound **5n**

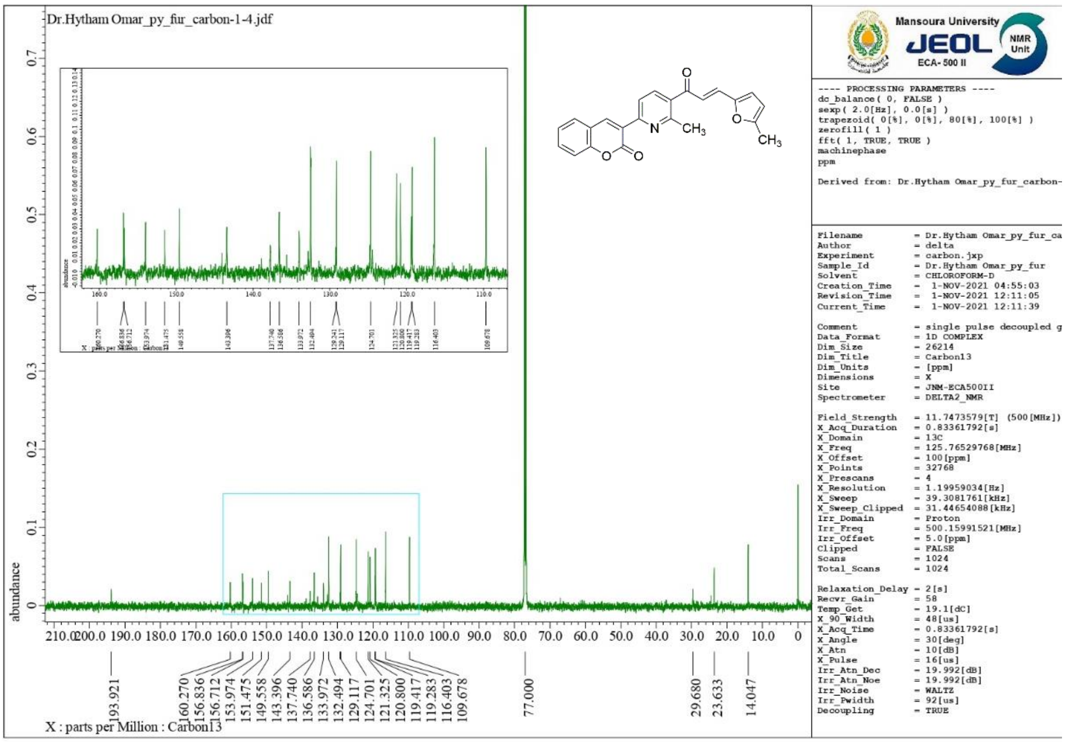

Figure S39. ^{13}C NMR (125 MHz, $CDCl_3$-*d*) spectrum of compound **5n**

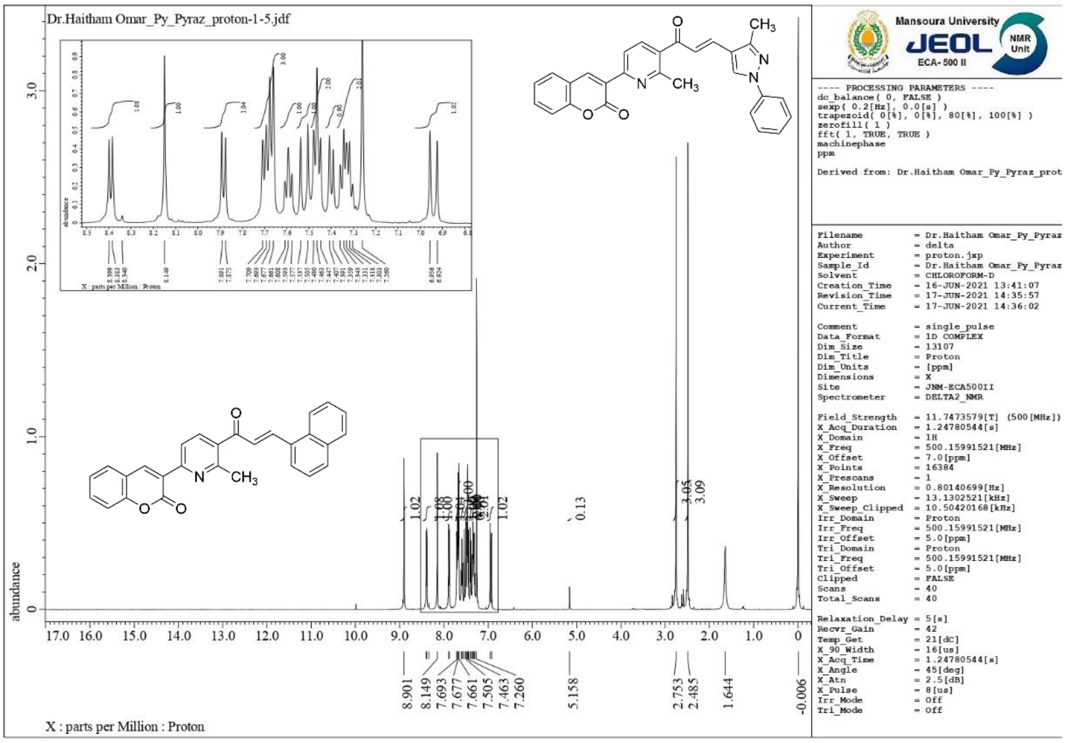

Figure S40. ^{1}H NMR (500 MHz, $CDCl_3$-*d*) spectrum of compound **5o**

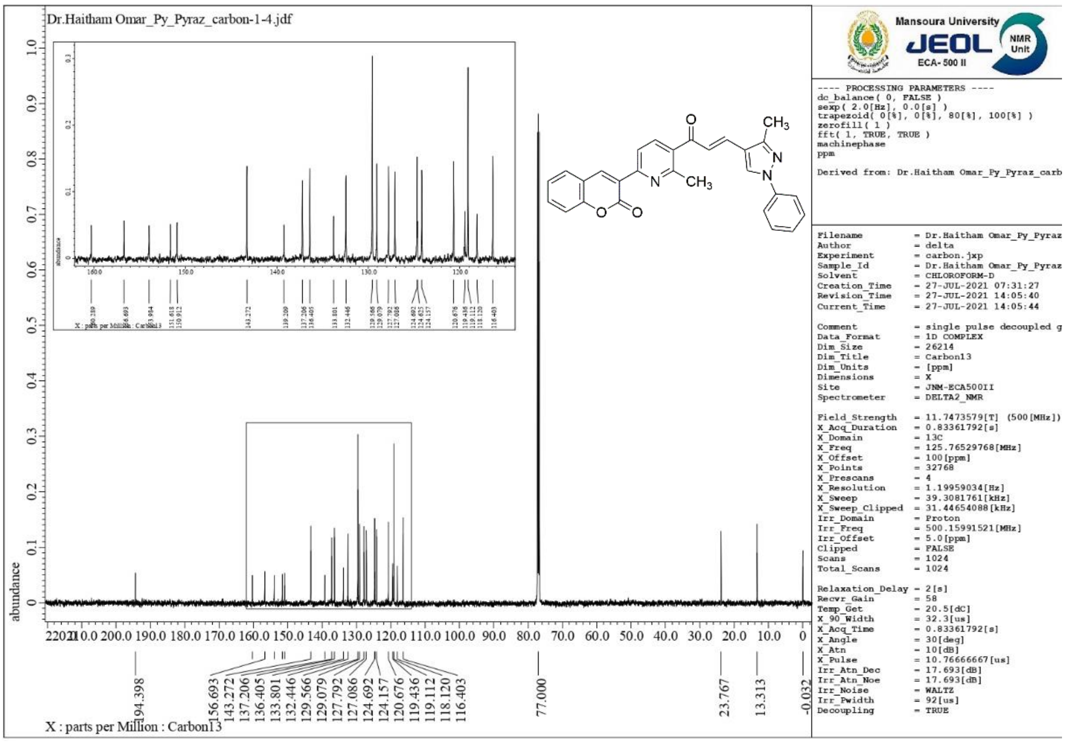

Figure S41. ^{13}C NMR (125 MHz, $CDCl_3$-*d*) spectrum of compound **5o**

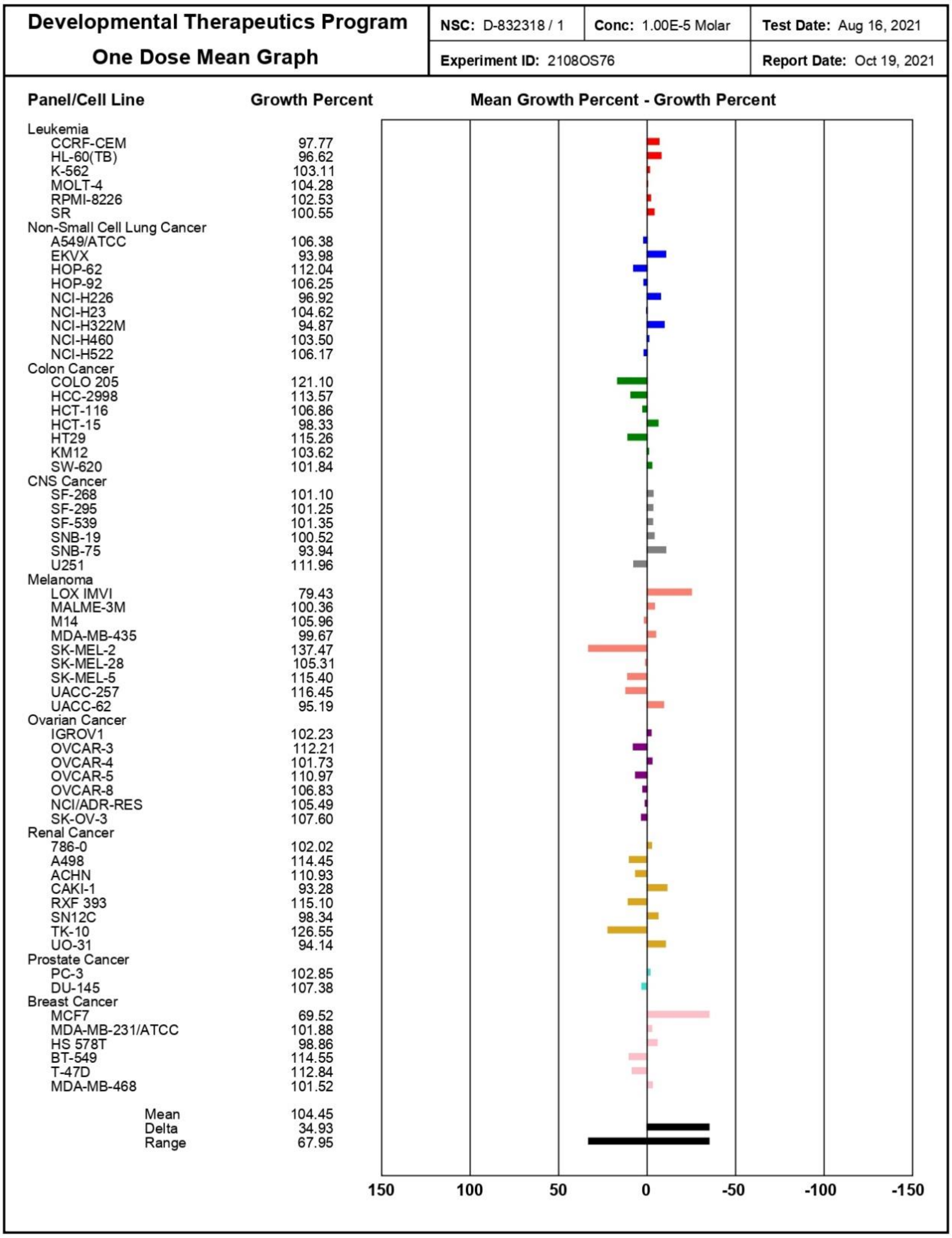

Figure S42. One dose mean graph for compound **3** (NSC 832318) at 10 μM

**Developmental Therapeutics Program**	**NSC:** D-831969 / 1	**Conc:** 1.00E-5 Molar	**Test Date:** Aug 09, 2021
**One Dose Mean Graph**	**Experiment ID:** 2108OS72		**Report Date:** Sep 15, 2021

Panel/Cell Line	Growth Percent
Leukemia	
CCRF-CEM	101.61
HL-60(TB)	103.70
K-562	95.11
MOLT-4	103.38
RPMI-8226	101.84
SR	103.55
Non-Small Cell Lung Cancer	
A549/ATCC	111.97
EKVX	98.84
HOP-62	103.08
HOP-92	94.82
NCI-H226	105.61
NCI-H23	100.13
NCI-H322M	100.87
NCI-H460	107.03
NCI-H522	95.48
Colon Cancer	
COLO 205	120.46
HCC-2998	101.61
HCT-116	97.94
HCT-15	98.17
HT29	109.98
KM12	103.50
SW-620	99.11
CNS Cancer	
SF-268	100.44
SF-295	101.21
SF-539	95.97
SNB-19	93.14
SNB-75	91.29
U251	97.60
Melanoma	
LOX IMVI	81.17
MALME-3M	103.07
M14	100.31
MDA-MB-435	96.04
SK-MEL-2	108.88
SK-MEL-28	109.19
SK-MEL-5	100.12
UACC-257	107.35
UACC-62	90.64
Ovarian Cancer	
IGROV1	108.40
OVCAR-3	107.77
OVCAR-4	100.98
OVCAR-5	103.30
OVCAR-8	103.18
NCI/ADR-RES	99.31
SK-OV-3	115.71
Renal Cancer	
786-0	98.64
A498	93.93
ACHN	107.90
CAKI-1	92.25
RXF 393	102.46
SN12C	94.63
UO-31	88.02
Prostate Cancer	
PC-3	107.68
DU-145	107.70
Breast Cancer	
MCF7	73.57
MDA-MB-231/ATCC	93.57
HS 578T	89.78
BT-549	104.37
T-47D	84.47
MDA-MB-468	96.84
Mean	100.15
Delta	26.58
Range	46.89

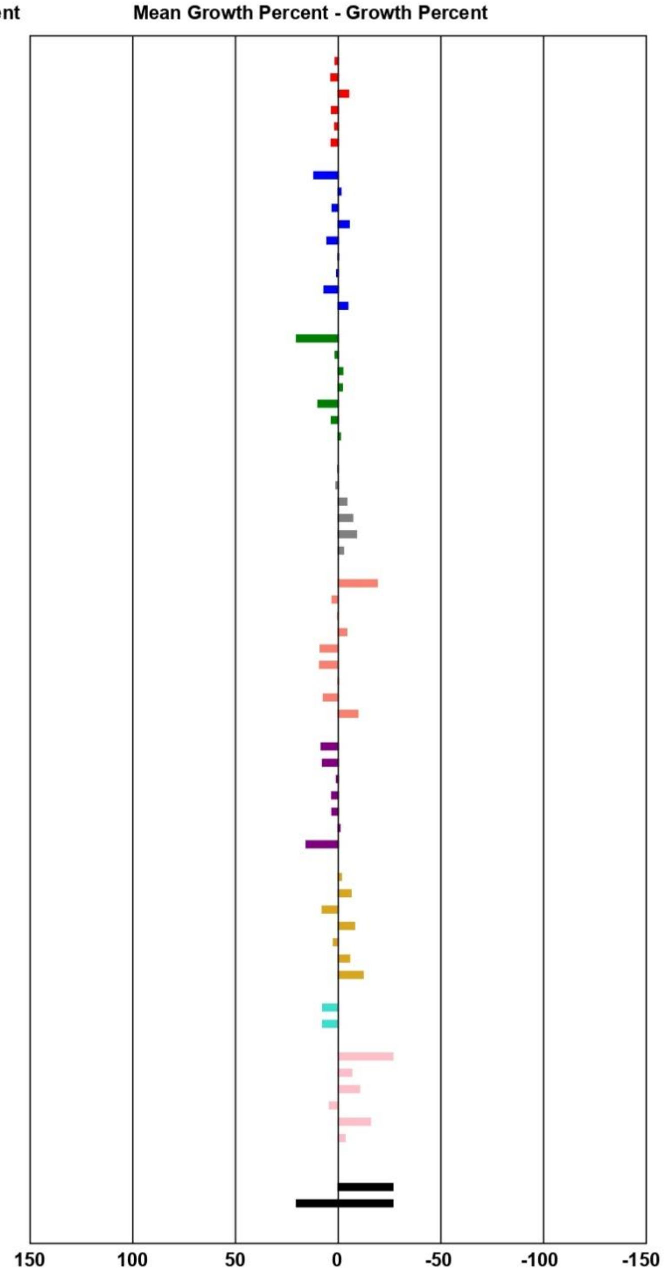

Figure S43. One dose mean graph for compound **5a** (NSC 831969) at 10 μM

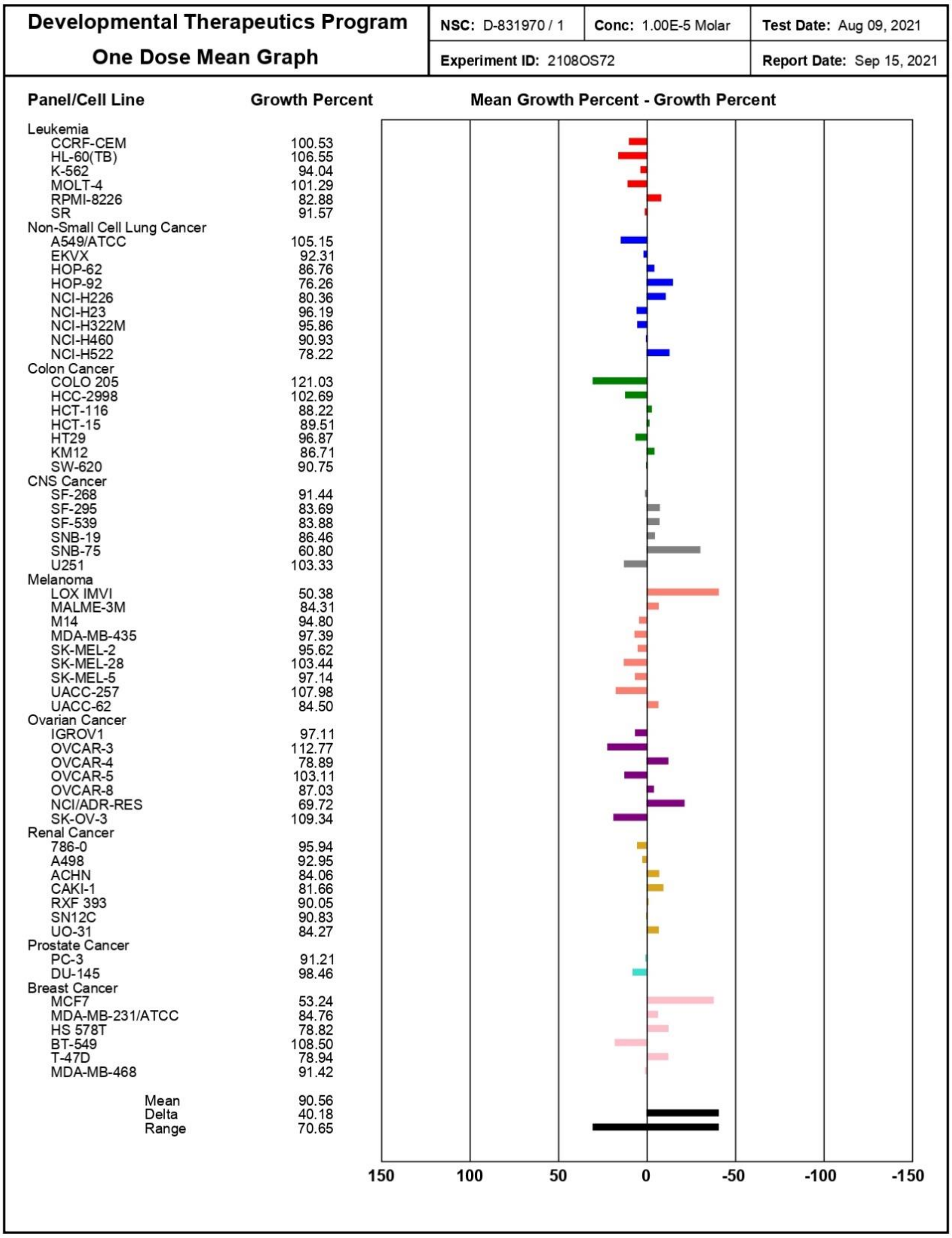

Figure S44. One dose mean graph for compound **5b** (NSC 831970) at 10 μM

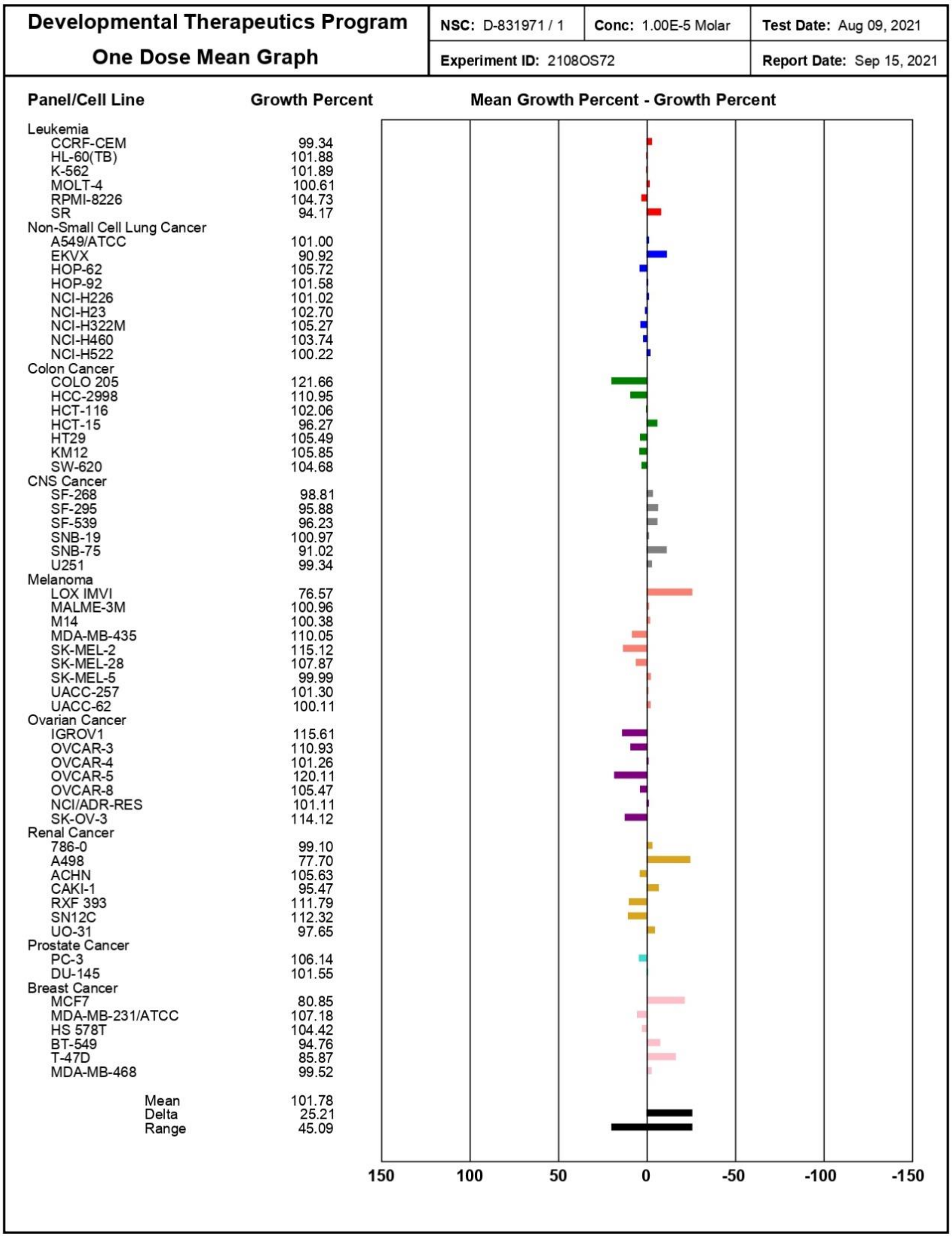

Figure S45. One dose mean graph for compound **5c** (NSC 831971) at 10 μM

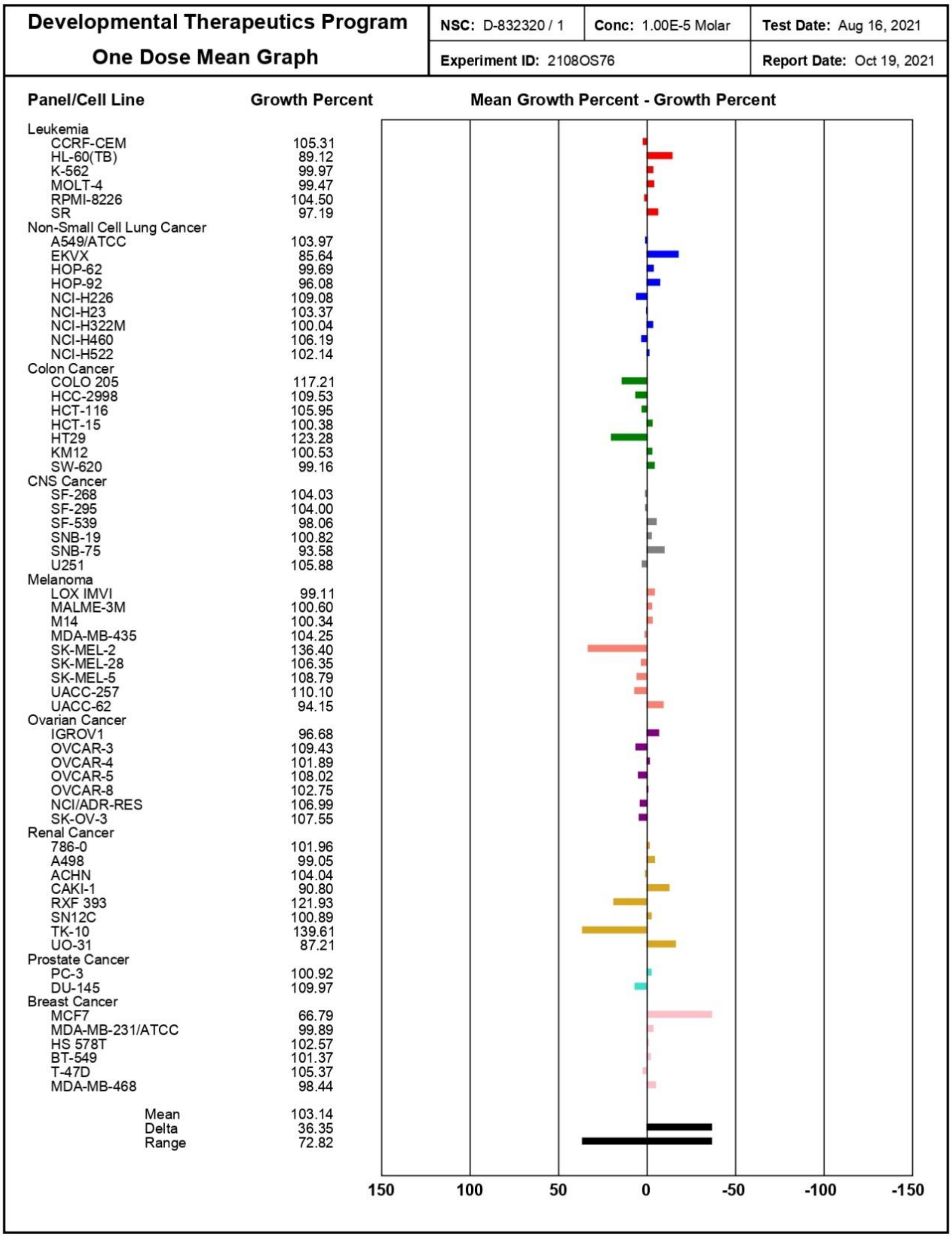

Figure S46. One dose mean graph for compound **5d** (NSC 832320) at 10 μM

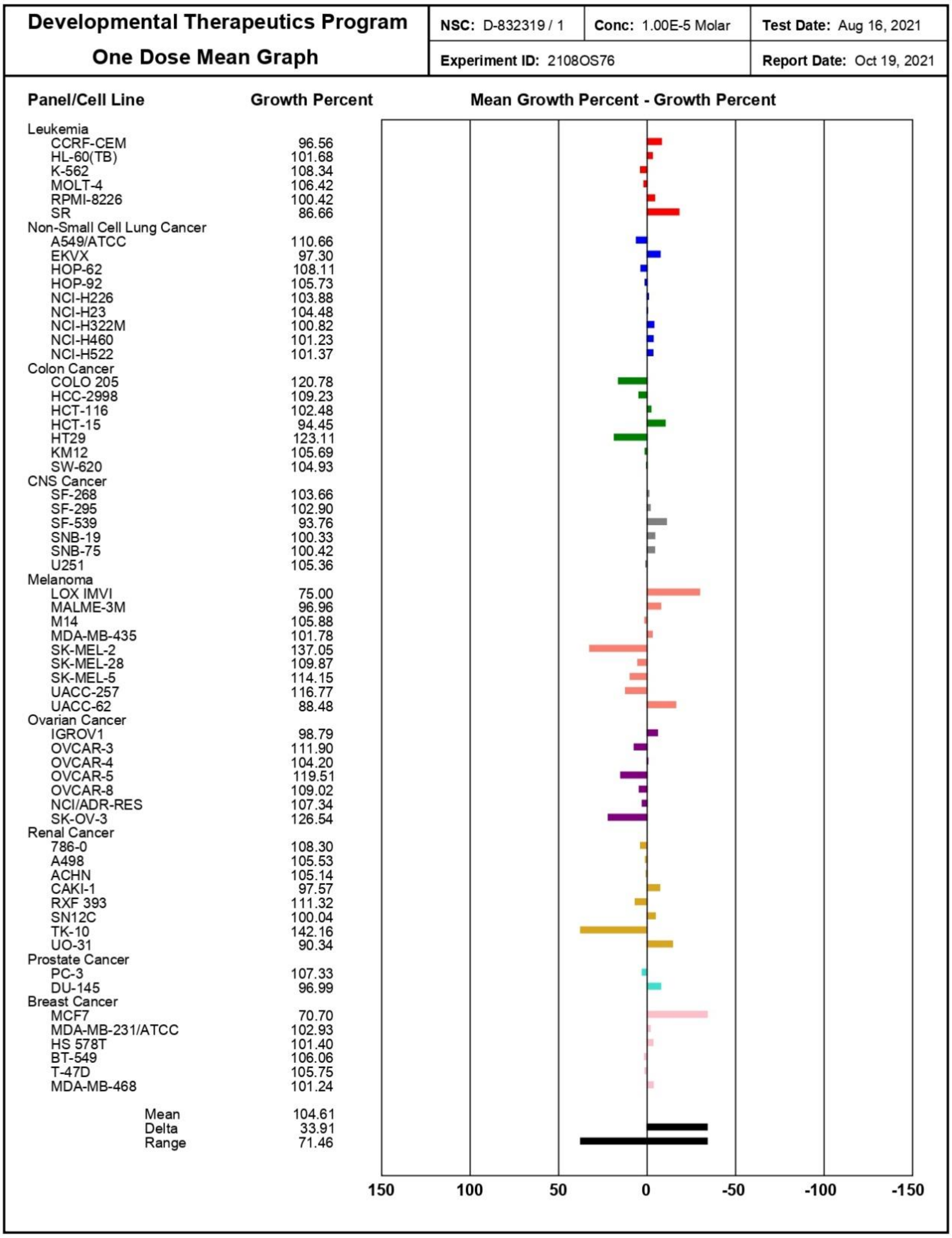

Figure S47. One dose mean graph for compound **5e** (NSC 832319) at 10 μM

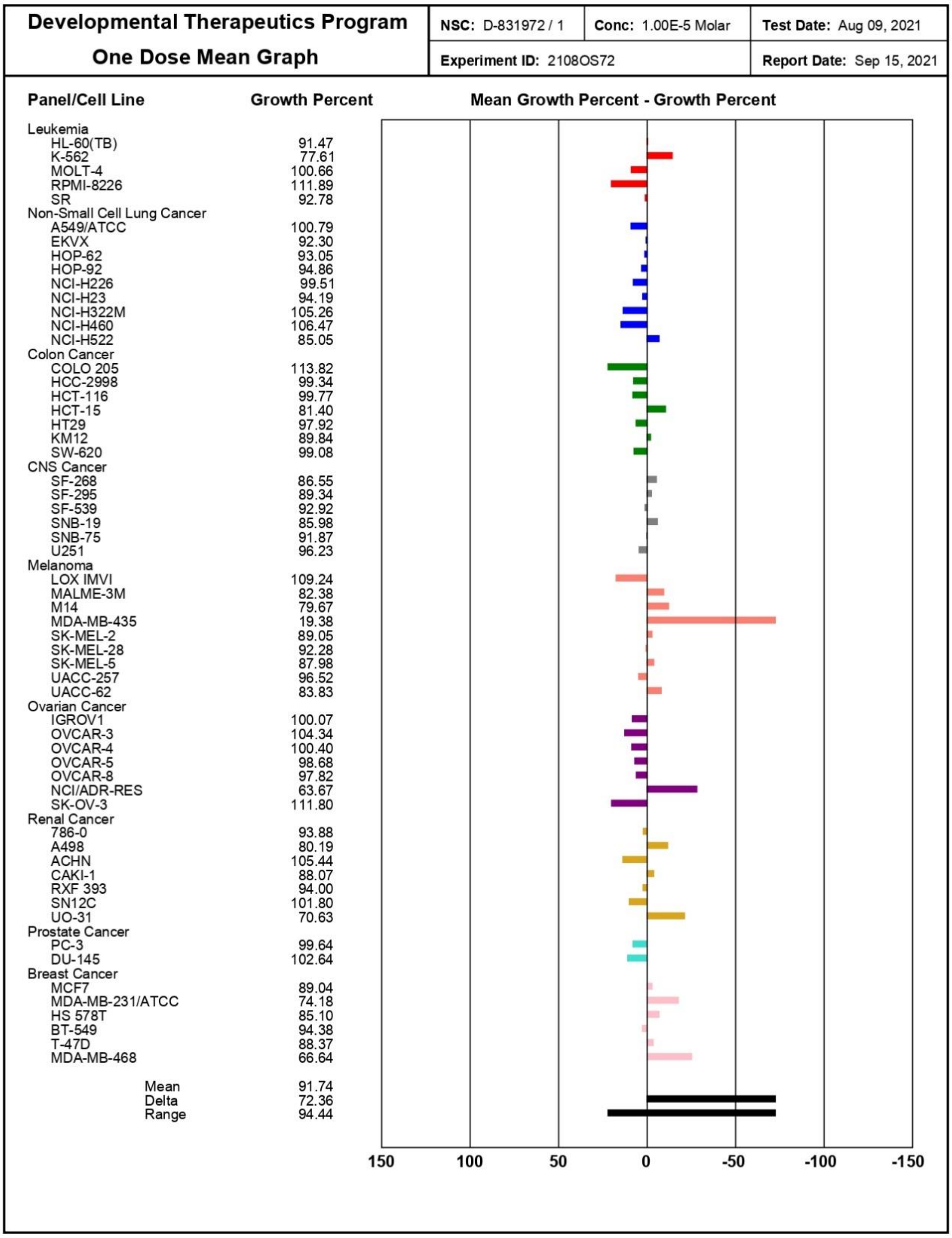

Figure S48. One dose mean graph for compound **5f** (NSC 831972) at 10 μM

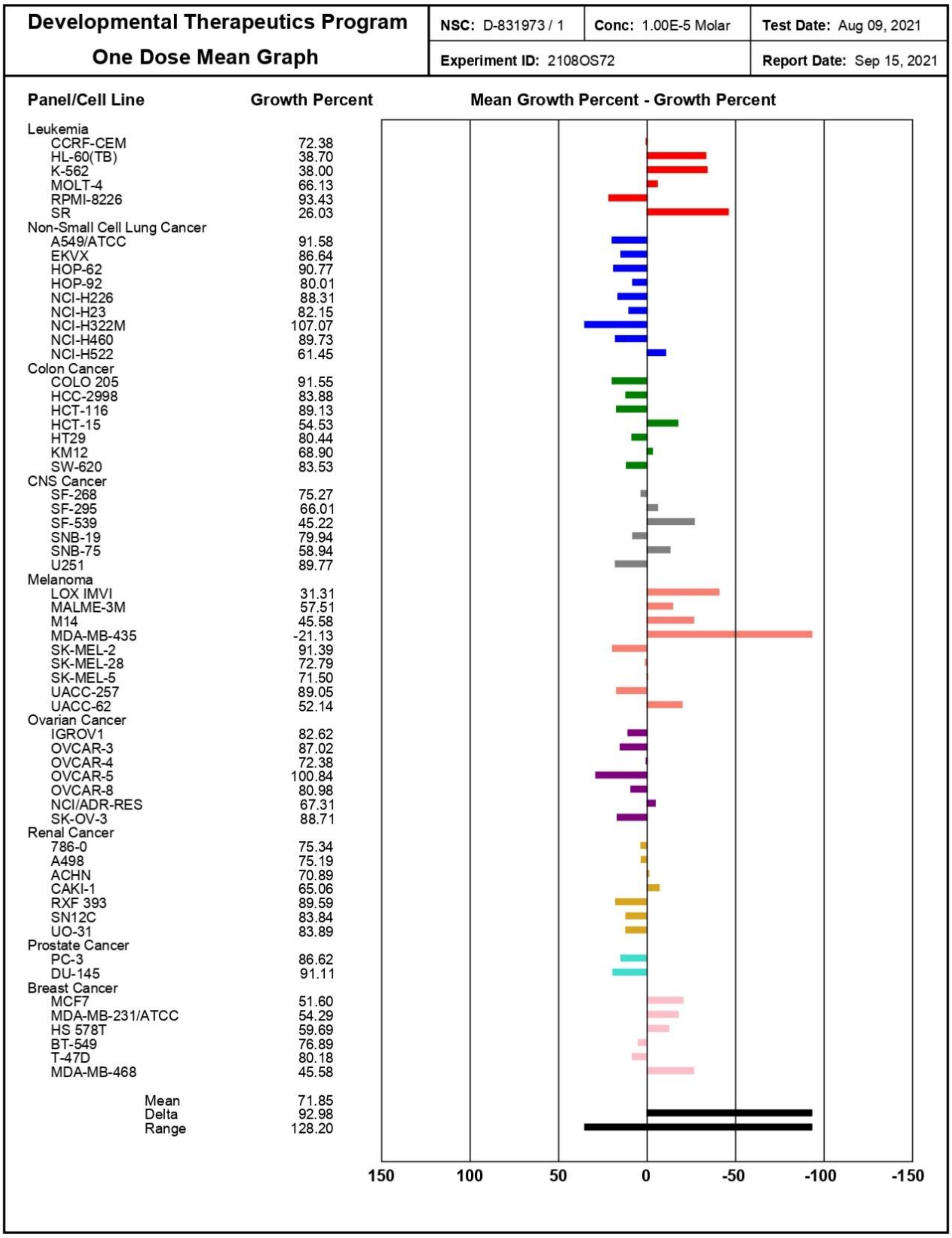

Figure S49. One dose mean graph for compound **5g** (NSC 831973) at 10 μM

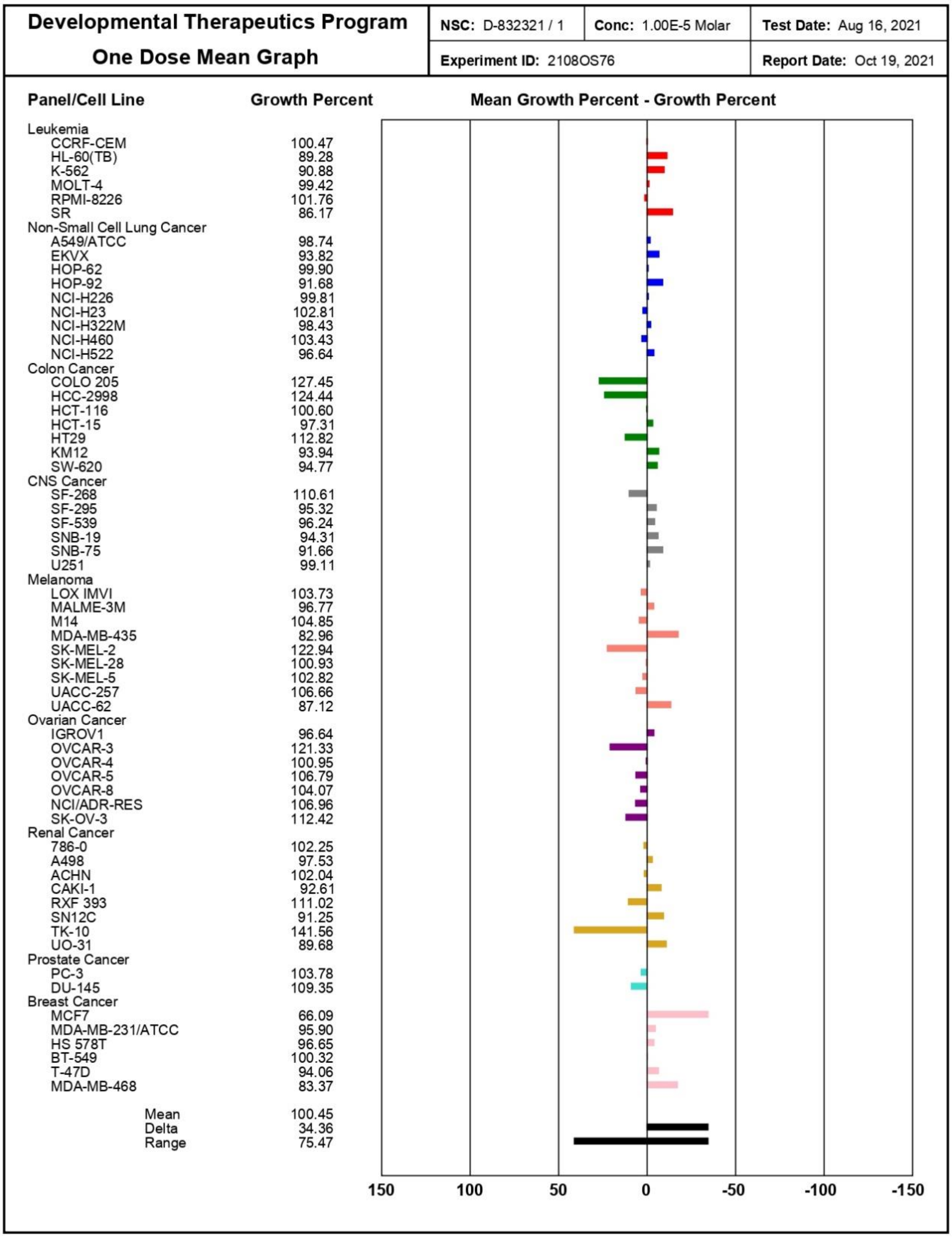

Figure S50. One dose mean graph for compound **5h** (NSC 832321) at 10 μM

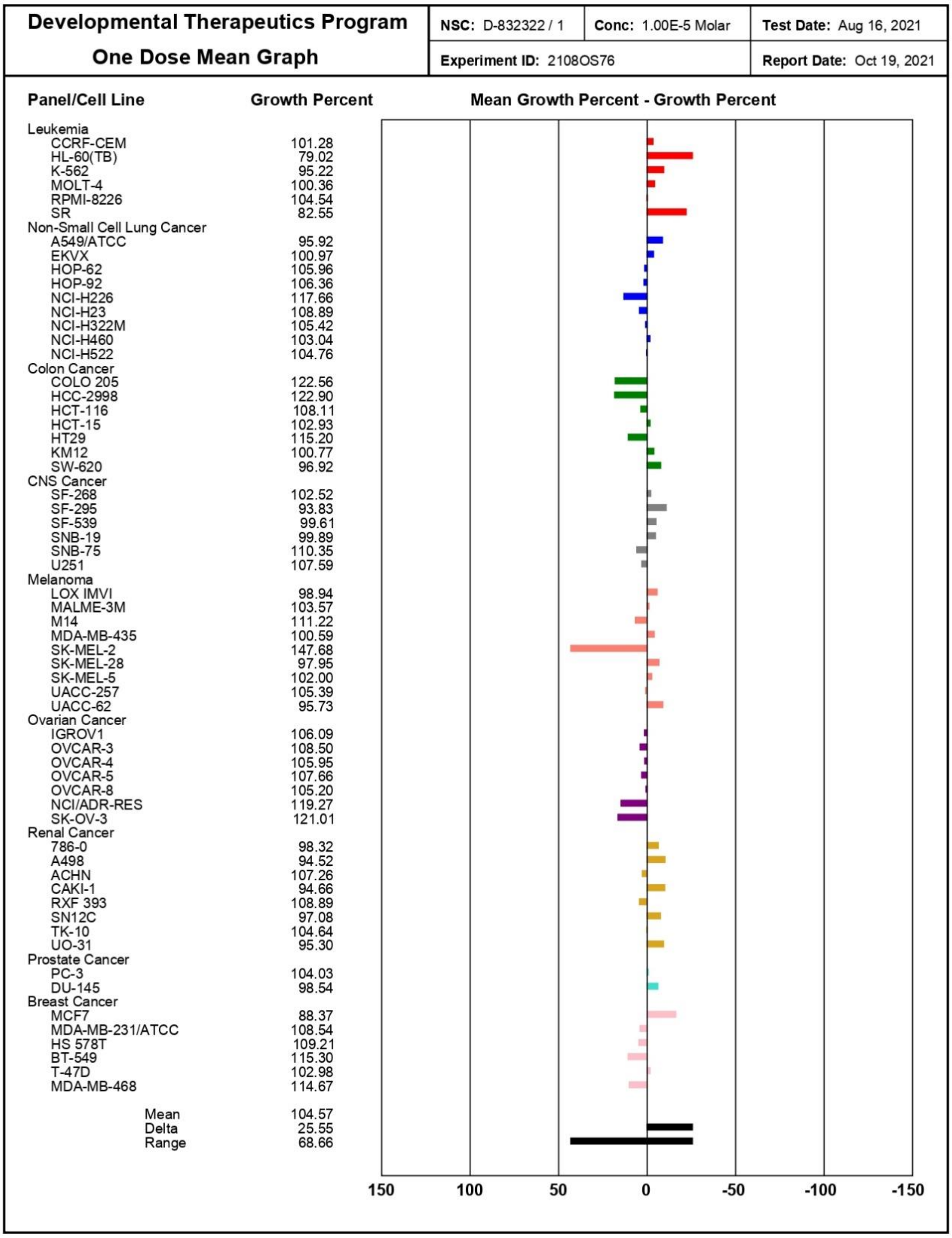

Figure S51. One dose mean graph for compound **5i** (NSC 832322) at 10 μM

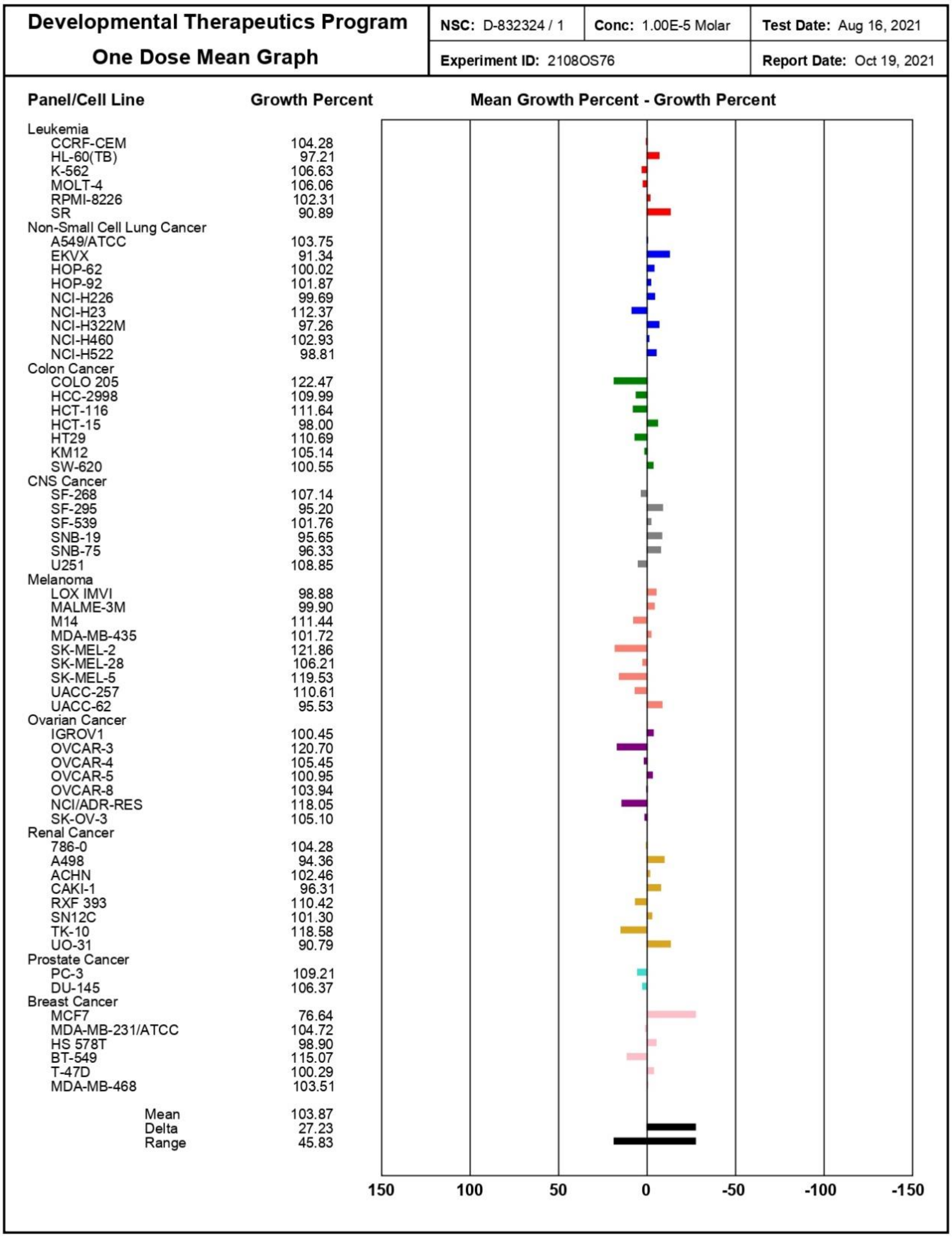

Figure S52. One dose mean graph for compound **5j** (NSC 832324) at 10 μM

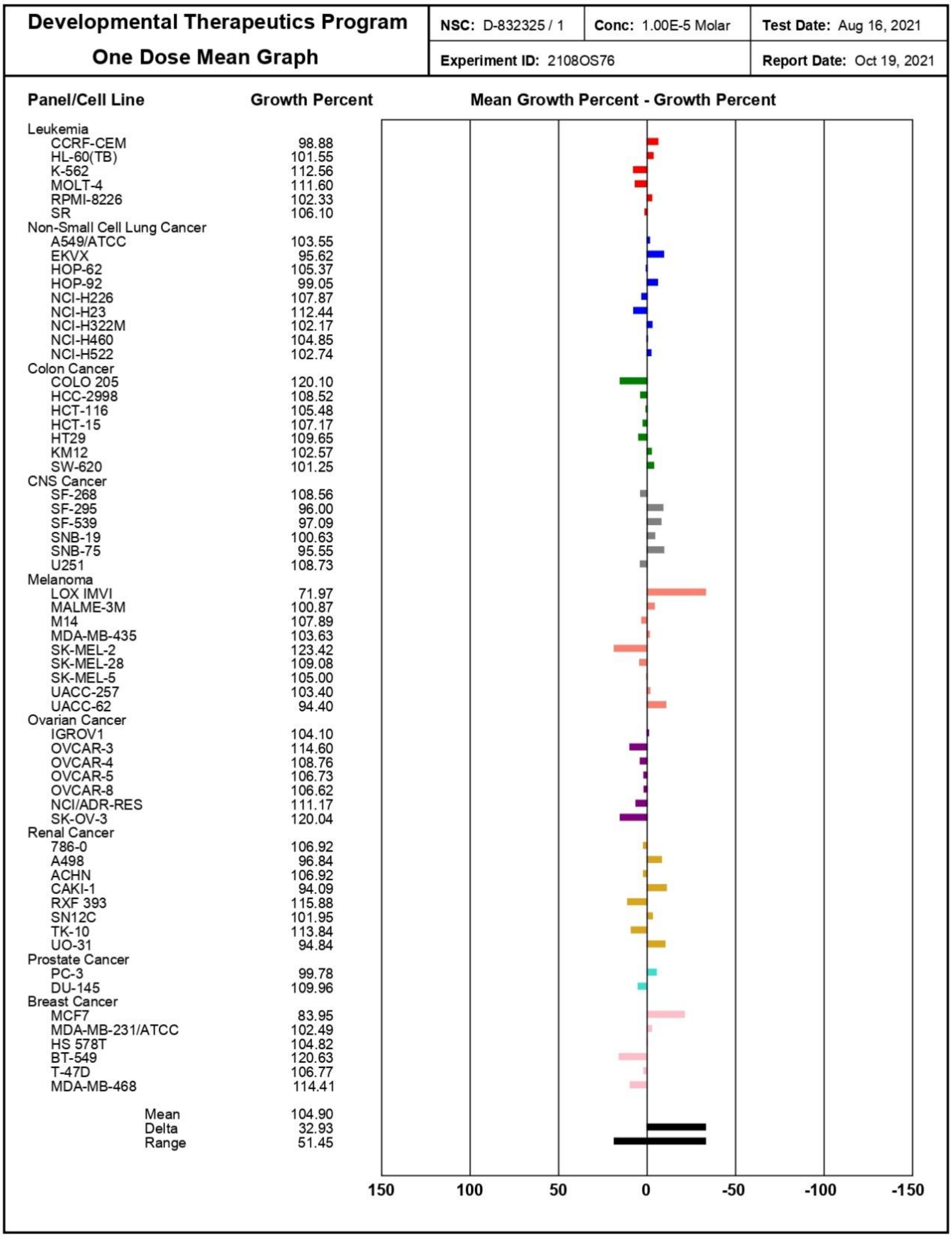

Figure S53. One dose mean graph for compound **5k** (NSC 832325) at 10 μM

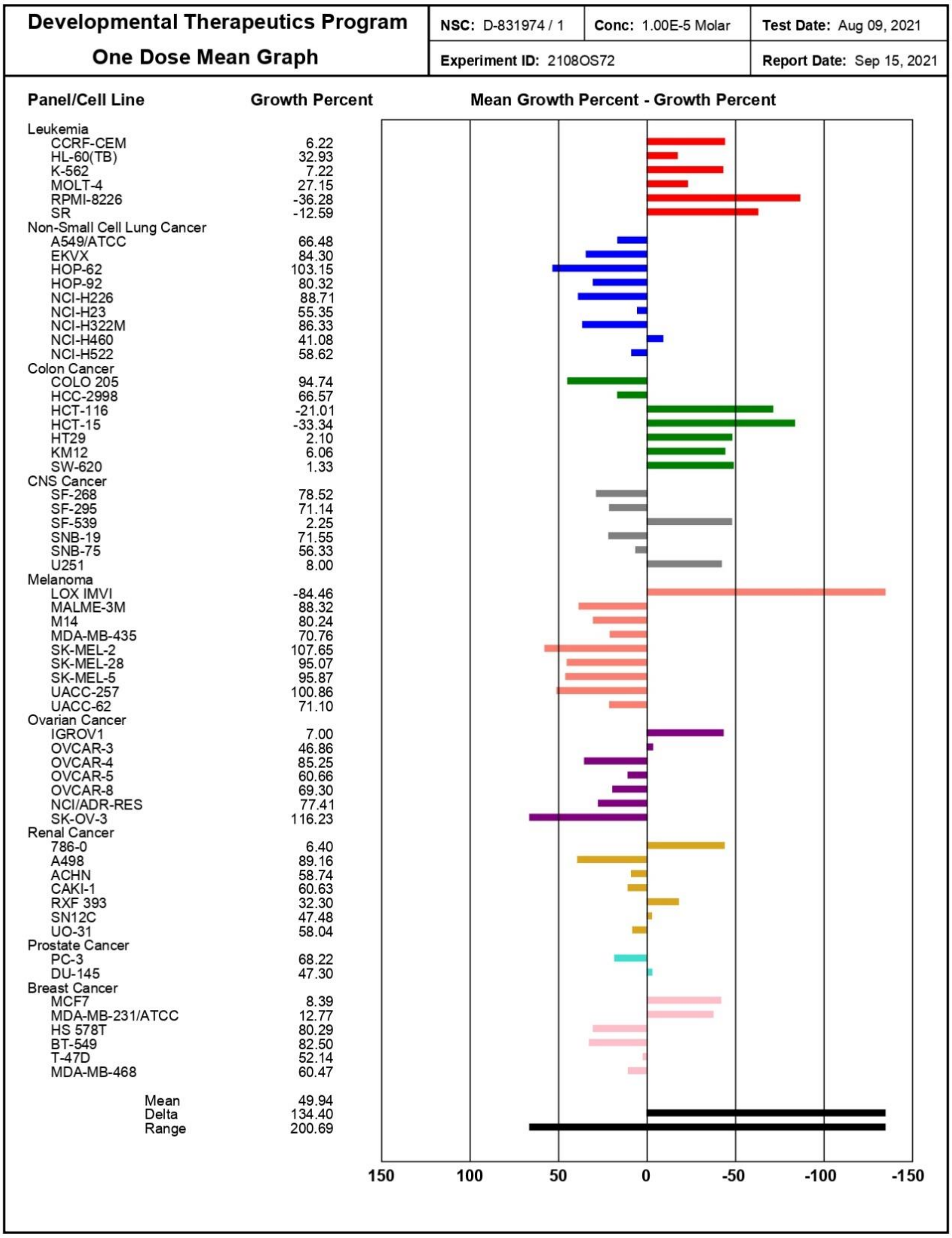

Figure S54. One dose mean graph for compound **5l** (NSC 831974) at 10 μM

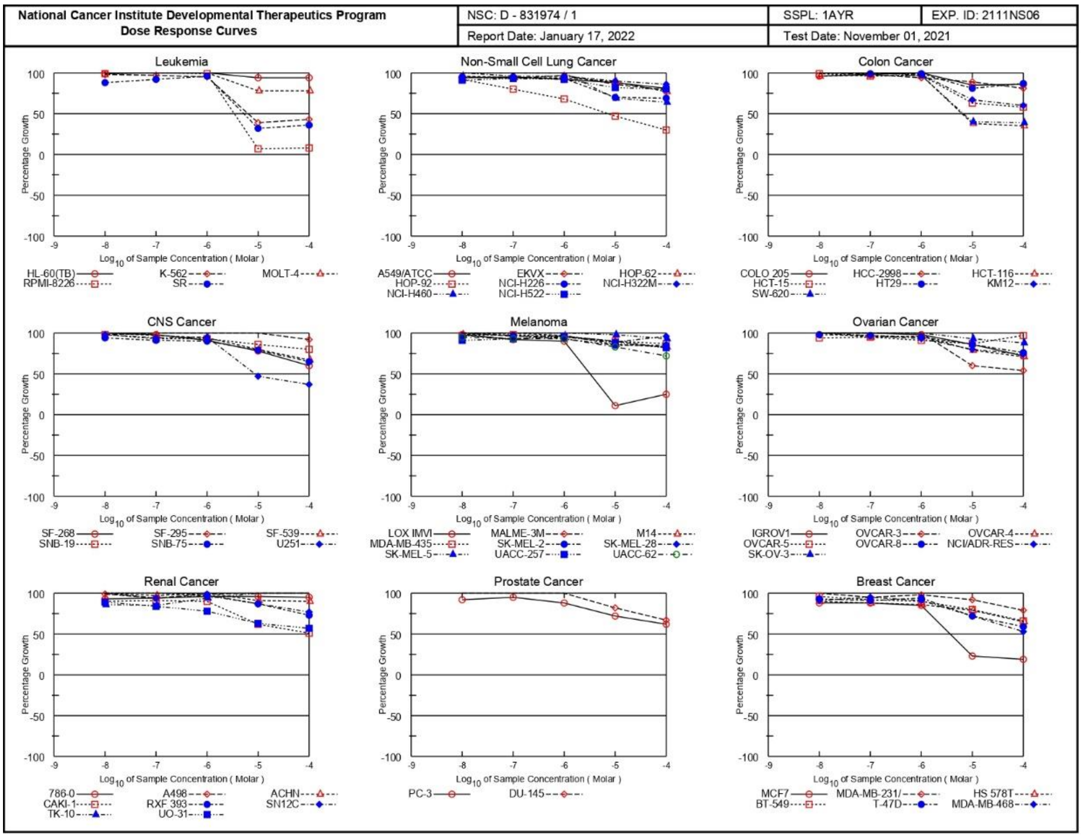

Figure S55. Dose-response curves (% growth versus sample concentration) for all cell lines with different subpanel obtained from the NCI's in vitro disease-oriented human cancer cells line for compound **5l** on nine types of cancer

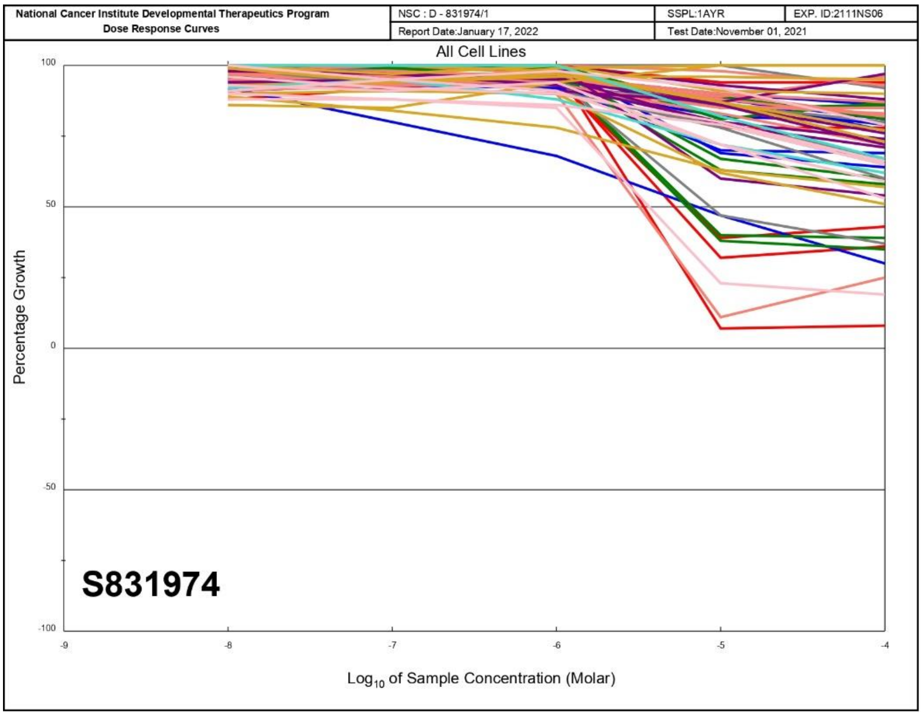

Figure S56. Dose-response curves for all cell lines in the NCI59 panel exposed compound **5l** with tissue originated colors and shapes.

National Cancer Institute Developmental Therapeutics Program
In-Vitro Testing Results

NSC : D - 831974 / 1	Experiment ID : 2111NS06	Test Type : 08	Units : Molar
Report Date : January 17, 2022	Test Date : November 01, 2021	QNS :	MC :
COMI : Py 5l	Stain Reagent : SRB Dual-Pass Related	SSPL : 1AYR	

	Time		Log10 Concentration												
			Mean Optical Densities					Percent Growth							
Panel/Cell Line	Zero	Ctrl	-8.0	-7.0	-6.0	-5.0	-4.0	-8.0	-7.0	-6.0	-5.0	-4.0	GI50	TGI	LC50
**Leukemia**															
HL-60(TB)	0.532	2.502	2.474	2.504	2.549	2.384	2.390	99	100	102	94	94	> 1.00E-4	> 1.00E-4	> 1.00E-4
K-562	0.114	1.200	1.183	1.165	1.148	0.543	0.578	98	97	95	39	43	6.47E-6	> 1.00E-4	> 1.00E-4
MOLT-4	0.419	2.105	2.097	2.178	2.214	1.734	1.730	100	104	106	78	78	> 1.00E-4	> 1.00E-4	> 1.00E-4
RPMI-8226	0.690	2.733	2.722	2.790	2.715	0.831	0.861	99	103	99	7	8	3.41E-6	> 1.00E-4	> 1.00E-4
SR	0.486	2.555	2.306	2.391	2.481	1.155	1.240	88	92	96	32	36	5.30E-6	> 1.00E-4	> 1.00E-4
**Non-Small Cell Lung Cancer**															
A549/ATCC	0.419	2.297	2.183	2.201	2.139	2.078	1.948	94	95	92	88	81	> 1.00E-4	> 1.00E-4	> 1.00E-4
EKVX	0.857	2.422	2.347	2.310	2.373	2.199	2.096	95	93	97	86	79	> 1.00E-4	> 1.00E-4	> 1.00E-4
HOP-62	0.847	2.467	2.397	2.371	2.371	2.289	2.100	96	94	94	89	77	> 1.00E-4	> 1.00E-4	> 1.00E-4
HOP-92	1.473	1.785	1.761	1.724	1.684	1.620	1.568	92	80	68	47	30	7.25E-6	> 1.00E-4	> 1.00E-4
NCI-H226	0.993	2.088	2.038	2.023	2.008	1.760	1.746	95	94	93	70	69	> 1.00E-4	> 1.00E-4	> 1.00E-4
NCI-H322M	0.704	2.050	2.049	1.996	1.998	1.916	1.866	100	96	96	90	86	> 1.00E-4	> 1.00E-4	> 1.00E-4
NCI-H460	0.167	1.805	1.868	1.831	1.891	1.295	1.216	104	102	105	69	64	> 1.00E-4	> 1.00E-4	> 1.00E-4
NCI-H522	1.018	2.870	2.701	2.740	2.720	2.535	2.491	91	93	92	82	80	> 1.00E-4	> 1.00E-4	> 1.00E-4
**Colon Cancer**															
COLO 205	0.400	1.604	1.560	1.581	1.633	1.426	1.438	96	98	102	85	86	> 1.00E-4	> 1.00E-4	> 1.00E-4
HCC-2998	0.490	1.785	1.799	1.756	1.708	1.643	1.540	101	98	94	89	81	> 1.00E-4	> 1.00E-4	> 1.00E-4
HCT-116	0.175	1.703	1.639	1.656	1.664	0.760	0.703	96	97	97	38	35	6.34E-6	> 1.00E-4	> 1.00E-4
HCT-15	0.339	2.475	2.464	2.397	2.415	1.693	1.573	99	96	97	63	58	> 1.00E-4	> 1.00E-4	> 1.00E-4
HT29	0.265	1.797	1.825	1.777	1.789	1.512	1.602	102	99	99	81	87	> 1.00E-4	> 1.00E-4	> 1.00E-4
KM12	0.172	1.832	1.903	1.847	1.797	1.284	1.169	104	101	98	67	60	> 1.00E-4	> 1.00E-4	> 1.00E-4
SW-620	0.201	1.525	1.521	1.537	1.489	0.735	0.711	100	101	97	40	39	6.77E-6	> 1.00E-4	> 1.00E-4
**CNS Cancer**															
SF-268	0.273	1.693	1.704	1.669	1.573	1.379	1.119	101	98	91	78	60	> 1.00E-4	> 1.00E-4	> 1.00E-4
SF-295	0.694	2.439	2.399	2.458	2.441	2.445	2.301	98	101	100	100	92	> 1.00E-4	> 1.00E-4	> 1.00E-4
SF-539	0.593	2.019	2.041	1.974	1.936	1.732	1.543	102	97	94	80	67	> 1.00E-4	> 1.00E-4	> 1.00E-4
SNB-19	0.677	2.154	2.131	2.054	2.044	1.949	1.856	98	93	93	86	80	> 1.00E-4	> 1.00E-4	> 1.00E-4
SNB-75	0.780	1.767	1.705	1.680	1.669	1.560	1.419	94	91	90	79	65	> 1.00E-4	> 1.00E-4	> 1.00E-4
U251	0.319	1.835	1.801	1.743	1.757	1.025	0.887	98	94	95	47	37	8.49E-6	> 1.00E-4	> 1.00E-4
**Melanoma**															
LOX IMVI	0.237	1.599	1.569	1.486	1.456	0.390	0.574	98	92	90	11	25	3.20E-6	> 1.00E-4	> 1.00E-4
MALME-3M	0.581	1.132	1.129	1.110	1.112	1.077	1.034	100	96	96	90	82	> 1.00E-4	> 1.00E-4	> 1.00E-4
M14	0.398	1.486	1.469	1.491	1.453	1.335	1.300	98	100	97	86	83	> 1.00E-4	> 1.00E-4	> 1.00E-4
MDA-MB-435	0.447	1.997	1.958	1.959	1.939	1.850	1.788	97	98	96	90	87	> 1.00E-4	> 1.00E-4	> 1.00E-4
SK-MEL-2	1.681	2.833	2.853	2.795	2.755	2.659	2.665	102	97	93	85	85	> 1.00E-4	> 1.00E-4	> 1.00E-4
SK-MEL-28	0.651	2.020	1.983	1.920	1.955	1.876	1.971	97	93	95	89	96	> 1.00E-4	> 1.00E-4	> 1.00E-4
SK-MEL-5	0.994	3.330	3.328	3.325	3.331	3.280	3.169	100	100	100	98	93	> 1.00E-4	> 1.00E-4	> 1.00E-4
UACC-257	0.996	2.463	2.329	2.362	2.397	2.291	2.198	91	93	96	88	82	> 1.00E-4	> 1.00E-4	> 1.00E-4
UACC-62	0.795	2.915	2.814	2.761	2.762	2.545	2.330	95	93	93	83	72	> 1.00E-4	> 1.00E-4	> 1.00E-4
**Ovarian Cancer**															
IGROV1	0.564	2.320	2.344	2.347	2.279	2.076	1.827	101	102	98	86	72	> 1.00E-4	> 1.00E-4	> 1.00E-4
OVCAR-3	0.211	1.418	1.458	1.438	1.421	0.935	0.866	103	102	100	60	54	> 1.00E-4	> 1.00E-4	> 1.00E-4
OVCAR-4	0.561	1.442	1.462	1.399	1.407	1.258	1.190	102	95	96	79	71	> 1.00E-4	> 1.00E-4	> 1.00E-4
OVCAR-5	0.617	1.780	1.708	1.721	1.671	1.632	1.742	94	95	91	87	97	> 1.00E-4	> 1.00E-4	> 1.00E-4
OVCAR-8	0.608	2.695	2.661	2.641	2.582	2.396	2.188	98	97	95	86	76	> 1.00E-4	> 1.00E-4	> 1.00E-4
NCI/ADR-RES	0.422	1.381	1.396	1.352	1.316	1.187	1.129	102	97	93	80	74	> 1.00E-4	> 1.00E-4	> 1.00E-4
SK-OV-3	0.925	1.922	1.936	1.951	1.919	1.847	1.806	101	103	100	93	88	> 1.00E-4	> 1.00E-4	> 1.00E-4
**Renal Cancer**															
786-0	0.618	2.335	2.215	2.231	2.262	2.265	2.248	93	94	96	96	95	> 1.00E-4	> 1.00E-4	> 1.00E-4
A498	1.997	2.844	2.835	2.796	2.834	2.864	2.941	99	94	99	102	111	> 1.00E-4	> 1.00E-4	> 1.00E-4
ACHN	0.344	1.764	1.771	1.719	1.790	1.638	1.622	100	97	102	91	90	> 1.00E-4	> 1.00E-4	> 1.00E-4
CAKI-1	0.393	1.564	1.447	1.463	1.451	1.119	0.992	90	91	90	62	51	> 1.00E-4	> 1.00E-4	> 1.00E-4
RXF 393	0.770	1.393	1.441	1.414	1.378	1.314	1.225	108	103	98	87	73	> 1.00E-4	> 1.00E-4	> 1.00E-4
SN12C	0.517	2.097	2.125	2.000	2.054	1.888	1.727	102	94	97	87	77	> 1.00E-4	> 1.00E-4	> 1.00E-4
TK-10	1.028	2.151	1.994	1.981	2.085	2.255	2.520	86	85	94	109	133	> 1.00E-4	> 1.00E-4	> 1.00E-4
UO-31	0.735	2.268	2.098	2.024	1.928	1.708	1.611	89	84	78	63	57	> 1.00E-4	> 1.00E-4	> 1.00E-4
**Prostate Cancer**															
PC-3	0.474	1.893	1.780	1.824	1.719	1.499	1.354	92	95	88	72	62	> 1.00E-4	> 1.00E-4	> 1.00E-4
DU-145	0.118	1.391	1.477	1.451	1.448	1.158	0.974	107	105	104	82	67	> 1.00E-4	> 1.00E-4	> 1.00E-4
**Breast Cancer**															
MCF7	0.290	1.722	1.555	1.544	1.512	0.626	0.560	88	88	85	23	19	3.72E-6	> 1.00E-4	> 1.00E-4
MDA-MB-231/ATCC	0.538	1.511	1.516	1.461	1.491	1.432	1.309	101	95	98	92	79	> 1.00E-4	> 1.00E-4	> 1.00E-4
HS 578T	0.863	2.008	1.897	1.873	1.849	1.769	1.607	90	88	86	79	65	> 1.00E-4	> 1.00E-4	> 1.00E-4
BT-549	1.069	2.209	2.158	2.123	2.095	1.977	1.826	96	92	90	80	66	> 1.00E-4	> 1.00E-4	> 1.00E-4
T-47D	0.989	2.365	2.265	2.236	2.285	1.975	1.797	93	91	94	72	59	> 1.00E-4	> 1.00E-4	> 1.00E-4
MDA-MB-468	0.936	1.517	1.467	1.489	1.466	1.355	1.242	91	95	91	72	53	> 1.00E-4	> 1.00E-4	> 1.00E-4

Figure S57. Values of log molar concentration of response parameters ($\log_{10}$ GI_{50}, $\log_{10}$ TGI & $\log_{10}$ LC_{50}) for compound **5l**

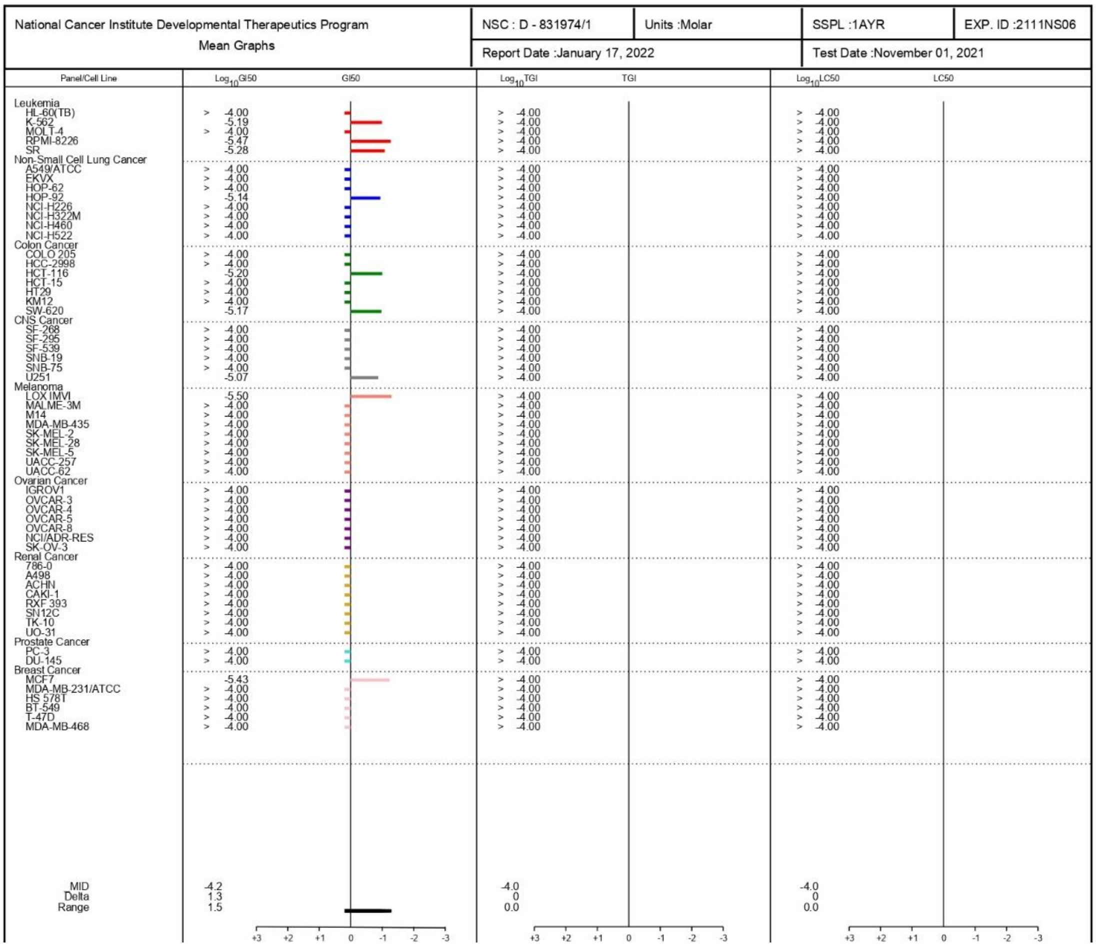

Figure S58. Mean Graphs of the $\log_{10}$ values (Molar) of GI_{50}, TGI, and LC_{50} obtained from the NCI 59 cell line experiments for compound **5l**

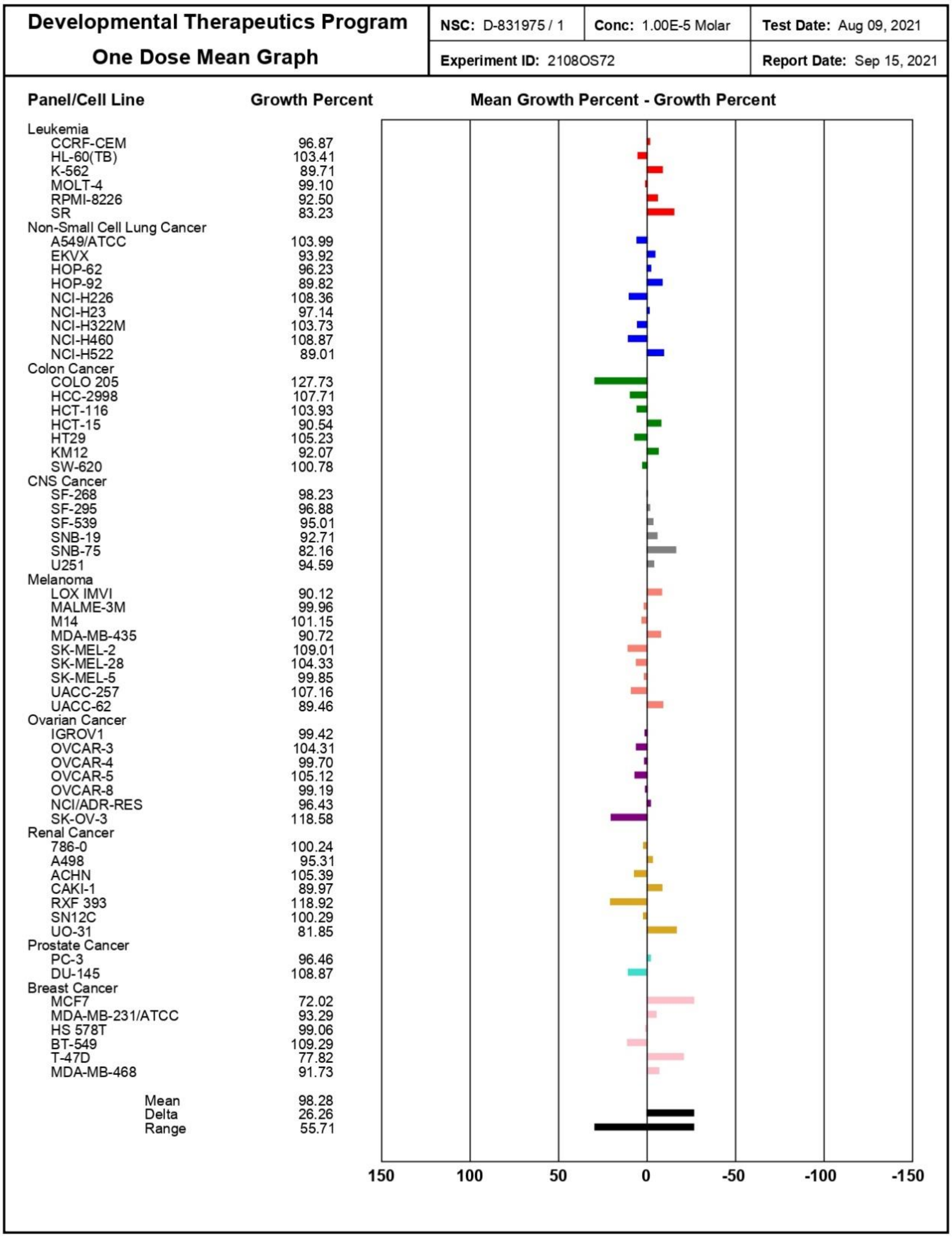

Figure S59. One dose mean graph for compound **5m** (NSC 831975) at 10 μM

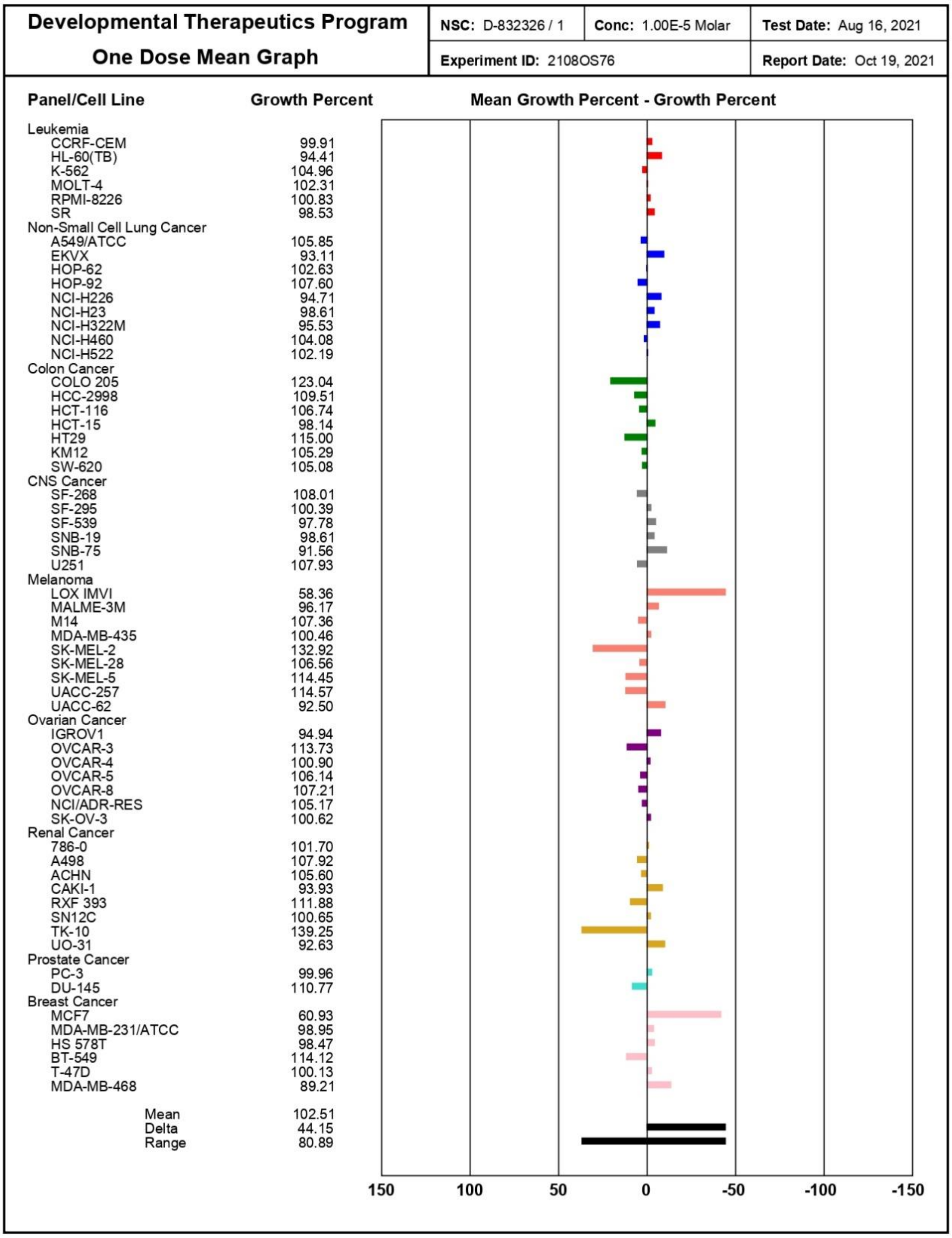

Figure S60. One dose mean graph for compound **5n** (NSC 832326) at 10 μM

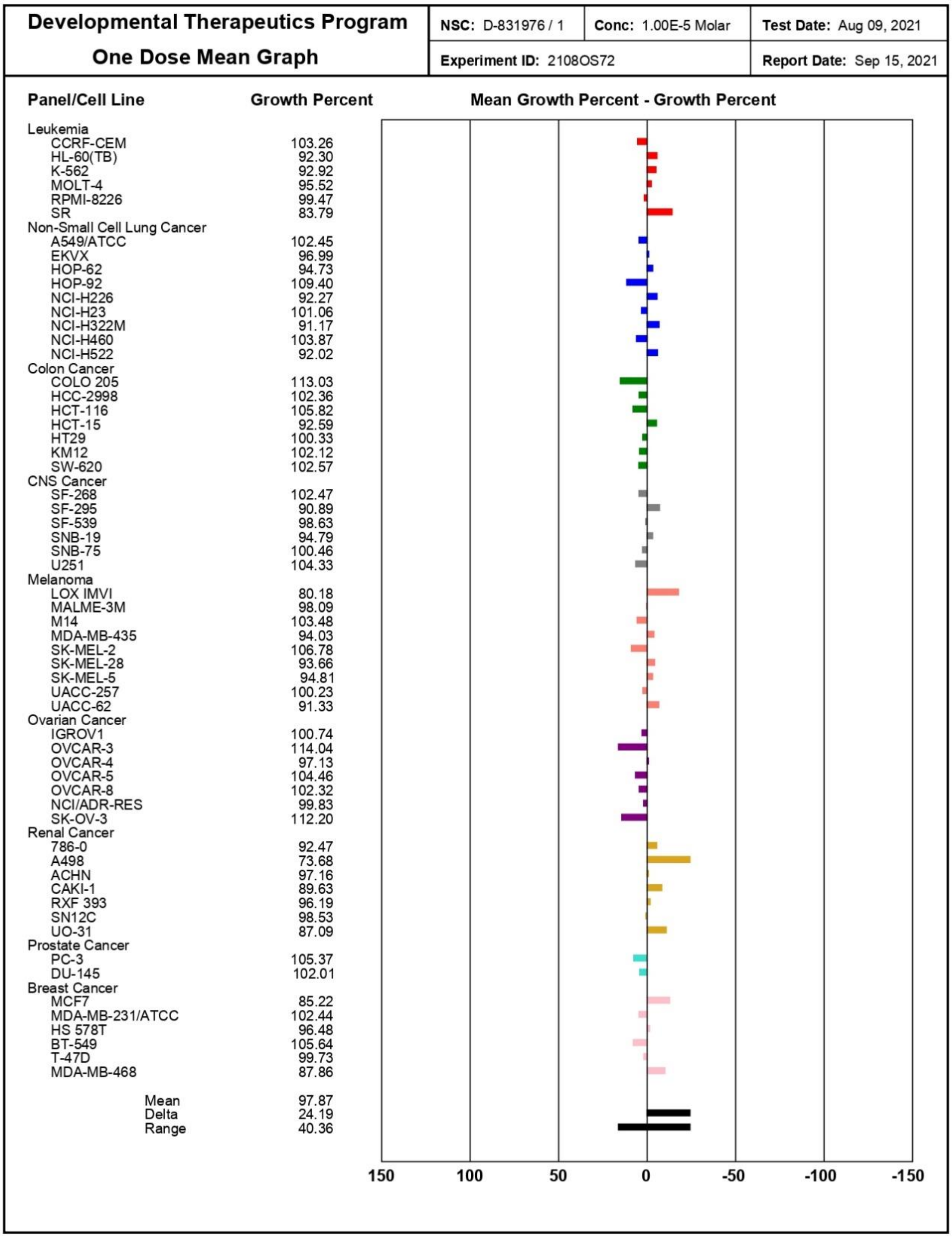

Figure S61. One dose mean graph for compound **5o** (NSC 831976) at 10 μM

Carbonic anhydrase inhibition assay

The carbonic anhydrase catalyzed CO_2 hydration actions for all coumarin-based derivatives reported in this study have assayed utilizing an instrument of Applied Photophysics stopped-flow. The enzymes are recombinant proteins prepared in our lab. Phenol red (at a concentration of 0.2 mM) has been used as indicator, working at the absorbance maximum of 557 nm, with 20 mM Hepes (pH 7.5) as buffer, and 20 mM Na_2SO_4 (for maintaining constant the ionic strength), following the initial rates of the CA-catalyzed CO_2 hydration reaction for a period of 10-100 s. The CO_2 concentrations ranged from 1.7 to 17 mM for the determination of the kinetic parameters and inhibition constants. For each inhibitor at least six traces of the initial 5-10% of the reaction have been used for determining the initial velocity. The uncatalyzed rates were determined in the same manner and subtracted from the total observed rates. Stock solutions of inhibitor (0.1 mM) were prepared in distilled-deionized water and dilutions up to 0.01 nM were done thereafter with the assay buffer. Inhibitor and enzyme solutions were preincubated together for 6 hrs. at room temperature prior to assay, in order to allow for the formation of the E-I complex. The inhibition constants were obtained by non-linear least-squares methods using PRISM 3 and the Cheng-Prusoff equation, and represent the mean from at least three different determinations.
